# Effects of Experimental Sleep Deprivation on Peripheral Inflammation: An Updated Meta‐Analysis of Human Studies

**DOI:** 10.1111/jsr.70099

**Published:** 2025-06-05

**Authors:** Andrea Ballesio, Valeria Fiori, Caterina Lombardo

**Affiliations:** ^1^ Department of Psychology Sapienza University of Rome Rome Italy

**Keywords:** C‐reactive protein, cytokines, immune system, interleukin‐6, sleep, tumour necrosis factor

## Abstract

A precise understanding of the effects of experimental sleep deprivation on inflammation is necessary to refine theoretical perspectives on sleep‐related immunopathological processes and implement robust empirical procedures. Here, we report an updated preferred reporting items for systematic reviews and meta‐analysis systematic review and meta‐analysis testing the effects of experimental total and partial sleep deprivation on circulating inflammatory markers in healthy adult individuals. PubMed, Scopus, PsycINFO, and CINAHL were searched up to March 2025. Data were analysed using the DerSimonian and Laird random effects approach. Of the 2264 articles retrieved, we included 35 studies reporting on 887 participants. Compared to normal sleep, multiple nights of experimental partial sleep deprivation (sleep duration reduced to ~4.30 h for 3+ nights) were associated with a significant increase of interleukin‐6 [IL‐6, *k* = 5, *d* = 0.42, [95% CI = 0.11 to 0.73], *p* < 0.01] and C‐reactive protein [CRP, *k* = 5, *d* = 0.76, [95% CI = 0.09 to 1.43], *p* = 0.03] in blood. A single night of total or partial sleep deprivation was not associated with changes in inflammation. Results suggest that the upregulation of inflammatory proteins in blood may only manifest following persistent periods of partial sleep deprivation. Further research will be needed to determine whether sleep recovery strategies (e.g., naps, sleep extension) may restore immune homeostasis. We suggest that experimental partial sleep deprivation for at least 3 nights may elicit peripheral IL‐6 and CRP and could therefore serve as a valid procedure to study sleep‐related immunopathological processes.

## 1 Introduction

Sleep deprivation is a recognised health problem of the contemporary era. Habitual short sleep duration (defined as ≤ 6 h) is reported by up to 33% of adults in the USA (Wang et al. 2023), with possible negative health outcomes, including hypertension, diabetes mellitus, cardiovascular and coronary heart diseases, obesity (Itani et al. 2017), major depression (Zhai et al. 2015), and mortality (He et al. 2020).

Pathophysiological pathways linking sleep deprivation to morbidity may include proinflammatory immune responses (Motivala 2011; Irwin 2015; Ballesio 2023a, 2023b). Inflammation is considered a primary biological response to physical (e.g., tissue damage, pathogens) and psychosocial (e.g., stressors) threats, which is orchestrated by the innate component of the immune system (Yin et al. 2015). Briefly, innate immune cells involved in the inflammatory response such as macrophages activate soluble mediators including cytokines (e.g., interleukins [IL], interferons) which increase the production of complement components and trigger systemic acute‐phase proteins such as C‐reactive protein (CRP, Patel et al. 2007) to coordinate an effective immune response. Notably, while temporally restricted activation of the inflammatory response is aimed at tissue regeneration and system homeostasis, chronic low‐grade inflammation may become a pathogenetic factor for physical and mental illness (Furman et al. 2019).

Lifestyle factors such as sedentary behaviour, smoking, high‐fat diet are known inflammatory drivers (Baechle et al. 2023). Also, the role of sleep loss as a potential challenge to the innate immune system has long been proposed (see Besedovsky et al. 2019 for a review). Experimental sleep deprivation and fragmentation may upregulate Toll‐like receptor‐4 stimulated monocyte intracellular proinflammatory cytokine production (Irwin et al. 2023), increase inflammatory signal transduction pathways (Mahalakshmi et al. 2022), and downregulate inflammatory resolution pathways (Engert et al. 2023) in randomised controlled trials. Notwithstanding, the role of sleep deprivation in upregulating the peripheral inflammatory response as measured using cytokines and acute phase proteins remains controversial. Only one meta‐analysis was conducted on the effects of experimental sleep deprivation on inflammatory measures (Irwin et al. 2016); in this meta‐analysis of seventeen studies, neither total nor partial sleep deprivation of one or multiple nights reliably increased peripheral proinflammatory markers. Crucially, the lack of robustness of experimental sleep deprivation in eliciting proinflammatory responses precludes a standardisation of empirical procedure and ultimately hinders the experimental study of sleep‐immune cross‐talks in the aetiology of diseases. Moreover, a clearer understanding of the effects of sleep deprivation on inflammation is needed to refine theoretical perspectives on sleep‐related immunopathological processes. To progress the field, we aimed to conduct an updated systematic review and meta‐analysis of human studies reporting on the effects of experimental total and partial sleep deprivation on peripheral measures of inflammation.

## 2 Methods

This study followed the Preferred Reporting Items for Systematic Reviews and Meta‐Analysis (PRISMA) guidelines (Moher et al. 2009, see checklist in Supporting Information) and was registered with the PROSPERO international database of the University of York Centre for Reviews (ID: CRD42024569254) (https://www.crd.york.ac.uk/prospero/).

### 2.1 Search Strategy

Searches were conducted on PubMed, Scopus, PsycINFO, and CINAHL from inception to 12th March 2025. Search terms were “sleep deprivation” OR “sleep restriction” OR “sleep disruption” OR “sleep curtailment” OR “sleep loss” AND cytokine* OR chemokine* OR interleukin* OR IL‐1 OR IL‐2 OR IL‐6 OR IL‐10 OR interferon* OR IFN OR “tumour necrosis factor*” OR “tumor necrosis factor*” OR TNF OR “C‐reactive protein” OR “C reactive protein” OR CRP OR hs‐CRP. Detailed search string is reported in Supporting Information. Additionally, the reference list of a previous meta‐analysis on the relationship between sleep and inflammation was screened for potential records (Irwin et al. 2016). The first author performed the literature search. The first and the second author independently screened titles and abstracts as well as full texts' reference list against eligibility criteria, and disagreement was resolved by discussion. In a similar manner, full‐texts' screening was performed independently by the two reviewers and disagreement in the evaluation was resolved by discussion.

### 2.2 Inclusion and Exclusion Criteria

Study eligibility was assessed using the PICOS approach (Richardson et al. 1995). To be included, studies had to fulfil the following inclusion criteria: (1) Population: healthy human participants (≥ 18 years) without current psychiatric or medical comorbidities; (2) Intervention: sleep deprivation performed by an experimental manipulation of sleep duration over one or several nights; (3) Comparison: control night(s) of normal sleep duration; (4) Outcomes: assessment of inflammation as an outcome by levels of circulating markers of inflammation (i.e., cytokines, chemokines, acute‐phase proteins). We focused on such proteins as the most commonly assessed with respect to sleep (Zhang et al. 2023); (5) Study design: both between‐ and within‐subjects studies were included; both randomised and non‐randomised studies were considered. Moreover, studies were included only if reporting data to compute pooled effect size of group differences. Samples with clinical sleep disorders were excluded. Studies on circadian misalignment (e.g., circadian rhythm disorders or habitual shift‐workers) were excluded. Grey literature was not considered.

### 2.3 Data Extraction

The first and the second authors extracted and double checked the following information from each included study using a standardised spreadsheet: authors; sample size; age; female percentages; body mass index (BMI); ethnicity; design (between‐ or within‐subjects); randomisation (yes/no); type of inflammatory outcome; biosample (serum, plasma, saliva); type of sleep deprivation (one/multiple nights, total, partial). Data needed for the effect size computations (i.e., means and standard deviations for both experimental and control groups in the morning after sleep manipulation on the inflammatory markers) were also extracted. When original articles reported data only in figures and/or graphs, data were converted to numerical values using Plot Digitizer software (http://plotdigitizer.sourceforge.net/). When data were not reported, authors of original papers were asked to provide them.

### 2.4 Quality Assessment

To evaluate the quality of the studies included in the meta‐analysis, we applied the Downs and Black Quality Index scoring system (Downs and Black 1998), a validated checklist designed to assess the quality of both randomised and non‐randomised studies. The tool encompasses five subscales—reporting, external validity, bias, confounding, and power—allowing for a maximum score of twenty‐five for non‐randomised, non‐prospective studies and a maximum score of twenty‐eight for randomised studies. In the current version of the checklist, we adjusted the scoring for item 27, which pertains to the study's power analysis according to the work of Korakakis et al. (2018). Rather than assigning scores based on a range of power values, we simply rated whether or not a power calculation was performed. As a result, the maximum score for item 27 became 1 (if a power analysis was conducted or the effect size was adequate) instead of 5, reducing the total possible score for the checklist to 28 (from 32).

### 2.5 Statistical Analysis

Data analysis was performed using the MAJOR module for Jamovi (Version 2.3, 2022, https://www.jamovi.org), based on R package “Metafor” (Viechtbauer 2010). For each study, we calculated the effect size (Cohen's d) indicating the standardised mean difference between sleep deprivation and control conditions at morning post sleep manipulation using a random‐effects model. Effect sizes were interpreted as small, moderate, and large using Cohen's (1992) definition. DerSimonian and Laird (1986) approach was implemented to take into account the true variation in effects occurring from study to study and the random errors within a single study. If a study reported multiple data in the same sample, we calculated the mean effect size using pooled weighted SD which was calculated as follows:

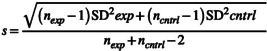

where $n_{\exp}$ and $n_{\text{cntrl}}$ indicate the number of participants and ${\mathrm{SD}}^{2}\exp$ and ${\mathrm{SD}}^{2}\text{cntrl}$ the SD points for the experimental (sleep deprivation) and control (normal sleep) group, respectively. Moreover, since some studies reported standard errors instead of SD as measures of dispersion, the latter was calculated as follows:

$$\mathrm{SD}=\mathrm{SE}*\sqrt{n}$$

To maximise the precision of estimates, analyses were run independently for each inflammatory marker under study, and separately for sleep manipulation procedure, that is, total/partial sleep deprivation for one/multiple nights (Irwin et al. 2016). The number of studies included in each analysis is reported with the letter *k*. Setting to three the number of three studies necessary to perform meta‐analyses (Crocetti 2016), the following effects could not be estimated due to lack of sufficient observations: (1) effects of one night of total (*k* = 0) and partial (*k* = 2) sleep deprivation on TNF‐α, (2) effect of one night of partial sleep deprivation on IL‐1β (*k* = 0), (3) effect of one night of partial sleep deprivation (*k* = 1) and one night of total sleep deprivation on IL‐8 (*k* = 0).

We checked for outliers by visually inspecting forest plots; outliers were defined as studies in which the 95% confidence interval around the effect size did not show overlap with the 95% confidence interval of the pooled effect size (Ballesio et al. 2021). To test heterogeneity, Cochran's *Q*, and Higgins's *I* ^2^ and *τ* ^2^ were calculated. Cochran's Q is computed as a weighted sum of squared differences between single study effects and the pooled effect across studies. Higgins's *I* ^2^ assesses the variability in effect estimates that is due to between‐study heterogeneity rather than due to chance, with higher levels of *I* ^2^ indicating higher heterogeneity. *τ* ^2^ represents a point estimate of the among‐study variance of true effects (Higgins 2008). When substantial amount of heterogeneity was detected in significant pooled effects, sensitivity analysis was conducted considering study characteristics.

## 3 Results

### 3.1 Study Selection

**FIGURE 1:**
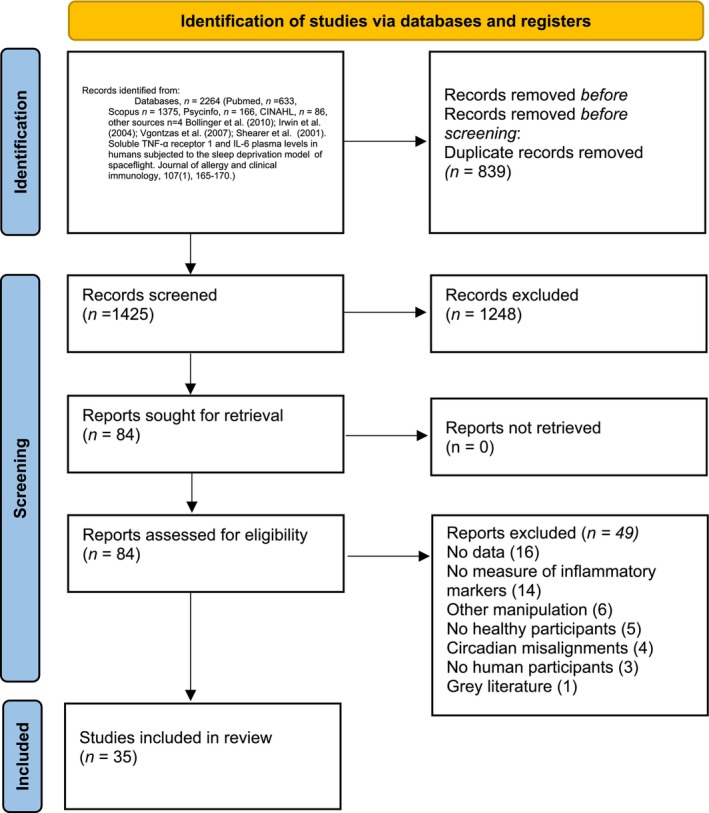
PRISMA 2020 flow diagram for new systematic reviews which included searches of databases and registers only. *Consider, if feasible to do so, reporting the number of records identified from each database or register searched (rather than the total number across all databases/registers). **If automation tools were used, indicate how many records were excluded by a human and how many were excluded by automation tools.

Of the 2264 records initially identified (PubMed, *n* = 633, Scopus *n* = 1375, PsycINFO, *n* = 166, CINAHL, *n* = 86, other sources *n* = 4), 35 (1.17%) met the inclusion criteria. Detailed search flow is reported in Figure 1. Information on excluded records is reported in Supporting Information.

### 3.2 Study Characteristics

Detailed information of included studies are reported in Table 1. The mean age of the participants was 26.93 ± 3.8 years. The overall percentage of females was 26.88%. For those reporting information on BMI, the mean value was 24.18 ± 2.8, reflecting normal weight (World Health Organization 2021). With respect to inflammatory markers, twenty‐two studies examined IL‐6 (Abedelmalek, Chtourou, et al. 2013; Abedelmalek, Souissi, et al. 2013; Barragán et al. 2023; Chennaoui et al. 2011; Cullen et al. 2020; Dáttilo et al. 2020; Frey et al. 2007; Haack et al. 2007; Matsubara et al. 2023; Matzner et al. 2013; Pejovic et al. 2013; Redwine et al. 2000; Said et al. 2019; Sauvet et al. 2010, 2015; Schmid et al. 2011; Simpson et al. 2016; Thompson et al. 2022; Vgontzas et al. 1999, 2004, 2007; Yang et al. 2021), fourteen studies examined CRP (Barragán et al. 2023; Baek et al. 2020; Boudjeltia et al. 2008; Chennaoui et al. 2011; Faraut et al. 2011; Frey et al. 2007; Haack et al. 2007; John‐Henderson et al. 2022; Meier‐Ewert et al. 2004; Mejri et al. 2017; Sauvet et al. 2010; Thompson et al. 2022; van Leeuwen et al. 2009; Yamazaki et al. 2021), 9 studies TNF‐α (Abedelmalek, Souissi, et al. 2013; Axelsson et al. 2013; Barragán et al. 2023; Chennaoui et al. 2011; Dáttilo et al. 2020; Said et al. 2019; Sauvet et al. 2010; Sauvet et al. 2015; Vgontzas et al. 2004 pooled), six studies examined IL‐1β (Axelsson et al. 2013; Dáttilo et al. 2020; Frey et al. 2007; Heiser et al. 1997, 2001; Said et al. 2019) and five studies examined IL‐8 (Faraut et al. 2011; Said et al. 2019; Sauvet et al. 2015; Wolkow et al. 2015; Yang et al. 2021). Furthermore, IL‐2 (Axelsson et al. 2013; Said et al. 2019) was investigated in two studies, while IL‐4, IL‐10, IL‐12, IL‐5, and IFN‐γ were examined in only one study each (Axelsson et al. 2013; Said et al. 2019). Therefore, we were unable to perform a statistical analysis on these cytokines.

**TABLE 1:** Description of included studies.

Study	Sample size	Mean age ± SD	BMI (kg/m^2^)	Female %	Etnicity	Design	Randomisation	Inflammatory marker(s)	Biosample	Type of sleep deprivation	Quality score
Abedelmalek, Chtourou, et al. 2013	12	21.20 ± 1.20	nr	nr	nr	Within subjects	Yes	IL‐6	Plasma	One night of partial sleep deprivation (22:30–03:00)	11
Abedelmalek, Souissi, et al. 2013	13	21.10 ± 0.83	nr	nr	nr	Within subjects	no	IL‐6, TNF‐α	Plasma	One night of partial sleep deprivation (22:30–03:00)	11
Axelsson et al. 2013	9	25.50 ± 0.83	nr	0%	nr	Within subjects	no	TNF‐α, IL‐2, IL‐4, IL‐1β	nr	Five nights of partial sleep deprivation (03:00–07:00)	10
Barragán et al. 2023	78	34.30 ± 12.50	25.8 ± 3.5	71.7%	41% white, 26% black, 22% Asian, 9% unknown	Within subjects	yes	CRP, IL‐6, TNF‐α	Plasma	One night of partial sleep deprivation	18
Baek et al. 2020	118	39.30 ± 3.10	22.2 ± 2	50%	nr	Within subjects	no	CRP	nr	Three nights of partial sleep deprivation (01:00–05:00)	15
Benedict et al. 2007	18	25.70 ± 1.50	nr	nr	nr	Within subjects	no	IL‐7	Serum	One night of total sleep deprivation	11
Boudjeltia et al. 2008	17	24.30 ± 2.70	nr	0%	nr	Between subjects	no	hs‐CRP	nr	Three nights of partial sleep deprivation (01:00–05:00)	13
Chennaoui et al. 2011	12	29.10 ± 3.30	23.4 ± 1.5	0%	nr	Within subjects	no	CRP, IL‐6, TNF‐α	Plasma	One night of total sleep deprivation	10
Cullen et al. 2020	10	27.00 ± 6.00	nr	0%	nr	Within subjects	yes	IL‐6	Plasma	One night of partial sleep deprivation and one night of total sleep deprivation	15
Dáttilo et al. 2020	10	24.50 ± 2.90	22.7 ± 2.3	0%	nr	Within subjects	Yes	IL‐6, IL‐1β, TNF‐α	Serum	Two night of partial sleep deprivation	15
Faraut et al. 2011	40	22.00 ± 1.00	nr	0%	nr	Between subjects	no	IL‐8, hs‐CRP	Serum	One night of partial sleep deprivation (02:00–04:00)	11
Frey et al. 2007	19	28.05 ± 8.56	nr	47%	nr	Within subjects	no	CRP, IL‐6, IL‐1β	Plasma	One night of total sleep deprivation	14
Haack et al. 2007	18	27.30 ± 5.80	23.1 ± 3.3	33.3%	nr	Between subjects	Yes	IL‐6, CRP	Plasma, serum	Twelve nights of partial sleep deprivation (23:00–03:00)	16
Heiser et al. 1997	10	27.40 ± 2.80	nr	0%	nr	Within subjects	No	IL‐1β	nr	One night of total sleep deprivation	11
Heiser et al. 2001	10	27.40 ± 2.80	nr	0%	nr	Within subjects	No	IL‐1β	nr	One night of total sleep deprivation	13
John‐Henderson et al. 2022	46	19.44 ± 2.11	nr	82.6%	91.3% white; 2.2 Asian; 2.2% American Indian/Alaskan native; 4.3% multiracial; 6.5% Hispanic	Between subjects	Yes	CRP	nr	One night of partial sleep deprivation (04:00–08:00)	17
Matsubara et al. 2023	28	27.00 ± 2.20	nr	100%	nr	Within subjects	no	IL‐6	Serum	One night of total sleep deprivation	10
Matzner et al. 2013	41	24.00 ± 1.86	nr	87.8%	nr	Between subjects	Yes	IL‐6, IL‐10	Plasma	One night of partial sleep deprivation	13
Meier‐Ewert et al. 2004	10	27.20 ± 2.50	nr	0%	nr	Within subjects	No	hs‐CRP	nr	Three night of partial sleep deprivation	15
Meier‐Ewert et al. 2004	10	30.10 ± 2.00	nr	40%	nr	Between subjects	Yes	hs‐CRP	nr	Ten nights of partial sleep deprivation (00:00–04:00)	15
Mejri et al. 2017	10	17.60 ± 0.52	nr	0%	nr	Within subjects	No	us‐CRP	Plasma	One night of partial sleep deprivation	15
Pejovic et al. 2013	30	24.70 ± 3.50	23.6 ± 2.4	46.6%	nr	Within subjects	No	IL‐6	Plasma	Six nights of partial sleep deprivation	17
Redwine et al. 2000	31	35.80 ± 10.12	nr	0%	81% white; 7% Philippines; 7% native American; 3% black; 3% Asian	Within subjects	No	IL‐6	Serum	One night of partial sleep deprivation	17
Said et al. 2019	8	26.00 ± 8.25	nr	50%	nr	Within subjects	No	IL‐2, IL‐4, IL‐6, IL‐ 5, IFN‐α, TNF‐γ, IL‐1β, IL‐10, IL‐12, IL‐8	Plasma	One week of partial sleep deprivation (5 h of sleep)	15
Sauvet et al. 2010	12	29.10 ± 3.30	23.4 ± 1.5	0%	nr	Within subjects	No	CRP, IL‐6, TNF‐α	Plasma	One night of total sleep deprivation	16
Sauvet et al. 2015	12	29.30 ± 5.20	23.8 ± 2.1	0%	nr	Within subjects	No	IL‐6	Plasma	Six nights of partial sleep deprivation (02:00–06:00)	13
Schmid et al. 2011	15	27.10 ± 1.30	22.9 ± 0.3	0%	nr	Within subjects	No	IL‐6	Serum	Two nights of partial sleep deprivation (02:45–07:00)	15
Simpson et al. 2016	14	26.50 ± 2.83	24.6 ± 0.7	nr	nr	Within subjects	Yes	il‐6	Plasma	Three weeks of partial sleep deprivation (03:00–07:00)	16
Thompson et al. 2022	23	20.78 ± 2.87	nr	39.1%	nr	Within subjects	No	CRP, IL‐6	Saliva	One night of total sleep deprivation	15
van Leeuwen et al. 2009	19	23.10 ± 2.50	nr	0%	nr	Between subjects	No	hs‐CRP	Plasma	Five nights of partial sleep deprivation (03:00–07:00)	14
Vgontzas et al. 1999	8	23.60 ± 1.00	25.6 ± 0.8	0%	nr	Within subjects	No	IL‐6	Plasma	One night of total sleep deprivation	15
Vgontzas et al. 2004	25	25.20 ± 3.75	23.8 ± 2.3	52%	nr	Within subjects	No	IL‐6, TNF‐α	Plasma	Eight nights of partial sleep deprivation (22:30–04:30)	15
Vgontzas et al. 2007	41	24.00 ± 2.00	nr	51.2%	nr	Within subjects	No	IL‐6	Plasma	One night of total sleep deprivation	15
Wolkow et al. 2015	35	39.00 ± 16.00	29.6 ± 5.5	14.2%	nr	Between subjects	Yes	IL‐8	Plasma	Two nights of partial sleep deprivation (02:00–06:00)	17
Yamazaki et al. 2021	32	35.10 ± 7.10	nr	43.7%	nr	Within subjects	No	CRP	Plasma	One night of total sleep deprivation	20
Yang et al. 2021	43	31.00 ± 2.00	24 ± 1	51.16%	nr	Between subjects	Yes	IL‐6, IL‐8	Plasma	Three nights of partial sleep deprivation (03:00–07:00)	20

Abbreviations: CRP = C‐reactive protein, IL‐6 = Interleukin‐6, nr = Not Reported, SD = Standard Deviation, TNF‐α = Tumour Necrosis Factor‐α.

Referring to the type of sleep deprivation, twelve studies implemented one experimental night of total sleep deprivation (Benedict et al. 2007; Chennaoui et al. 2011; Cullen et al. 2020; Frey et al. 2007; Heiser et al. 1997, 2001; Matsubara et al. 2023; Sauvet et al. 2010; Thompson et al. 2022; Vgontzas et al. 2007; Yamazaki et al. 2021), nine studies implemented one experimental night of partial sleep deprivation (Abedelmalek, Chtourou, et al. 2013; Abedelmalek, Souissi, et al. 2013; Barragán et al. 2023; Cullen et al. 2020; Faraut et al. 2011; John‐Henderson et al. 2022; Matzner et al. 2013; Mejri et al. 2017; Redwine et al. 2000) and fifteen studies implemented multiple experimental nights of partial sleep deprivation (Axelsson et al. 2013; Baek et al. 2020; Boudjeltia et al. 2008; Dáttilo et al. 2020; Haack et al. 2007; Meier‐Ewert et al. 2004; Pejovic et al. 2013; Sauvet et al. 2015; Schmid et al. 2011; Simpson et al. 2016; van Leeuwen et al. 2009; Vgontzas et al. 2004; Wolkow et al. 2015; Yang et al. 2021).

### 3.3 Study Quality

Individual scores on the Downs and Black Quality Index scoring system (Downs and Black 1998) are reported in Table 1. Detailed quality assessment for each study is reported in Supporting Information. Scores ranged from 10 to 20. In terms of weaknesses, most of the studies (*n* = 25, 71.4%) were non‐randomised and non‐blinded studies. Additionally, most of the included studies (*n* = 31, 88.5%) exhibited limitations in external validity, particularly due to the lack of details regarding the sample recruitment process. Moreover, critical issues were identified concerning the recognition of potential confounding factors and the use of strategies to manage these confounders (*n* = 29, 82.8%). Summarising the qualitative strengths, all the included studies provided a clear definition of study subjects and settings, along with a detailed description of the experimental procedure for sleep deprivation. Furthermore, all studies employed valid and reliable outcome measures and used appropriate statistical analyses.

### 3.4 Meta‐Analytic Results

#### 3.4.1 CRP

##### 3.4.1.1 Effects of One Night of Total Sleep Deprivation on CRP

Random effect model revealed non‐significant effect of one night of experimental total sleep deprivation on CRP [*k* = 5, *d* = −0.23, [95% CI = −0.65 to 0.19], *p* = 0.280], with substantial heterogeneity (*Q* = 8.308, df = 4, *p* = 0.081; *τ* ^2^ = 0.117, *I* ^2^ = 51.85%). Forest plot is reported in Table 2a.

**TABLE 2:** Forest plots showing individual study and global effect size estimates for different types of sleep deprivation on CRP, IL‐6, TNF‐α, IL‐1β, IL‐8. Results are reported as Cohen's d and 95% confidence intervals.

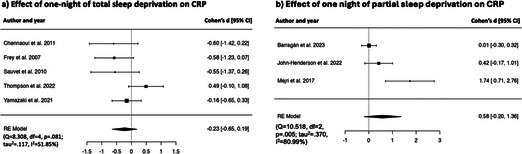
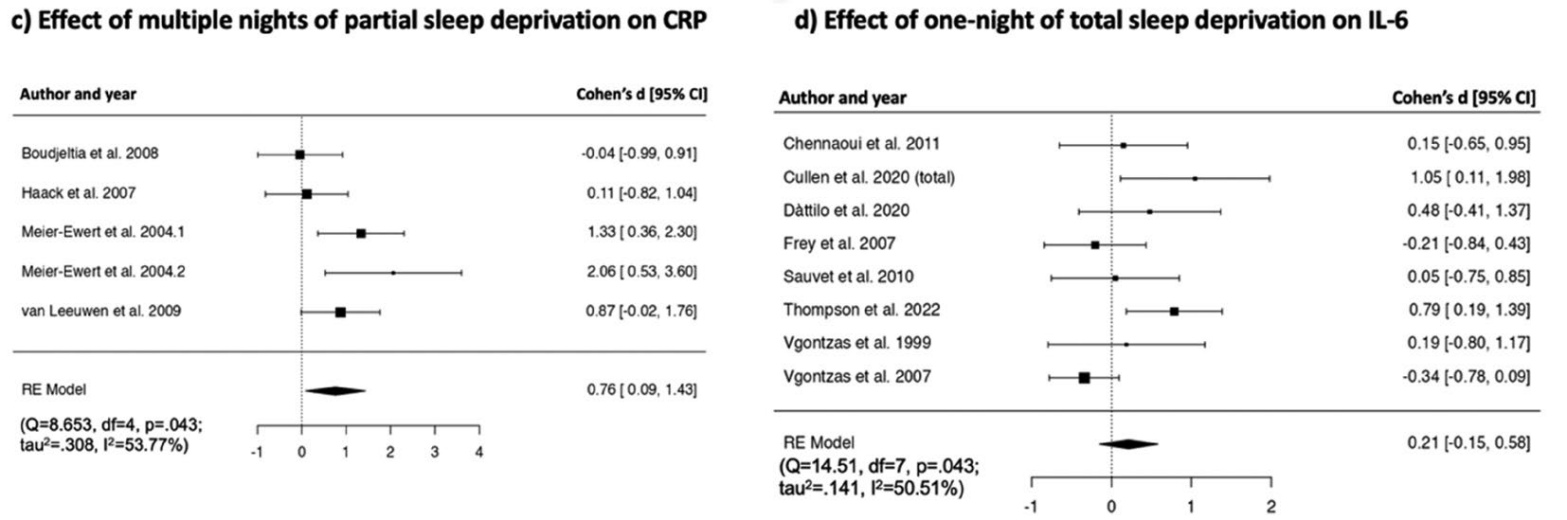
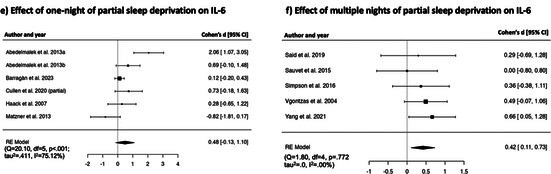
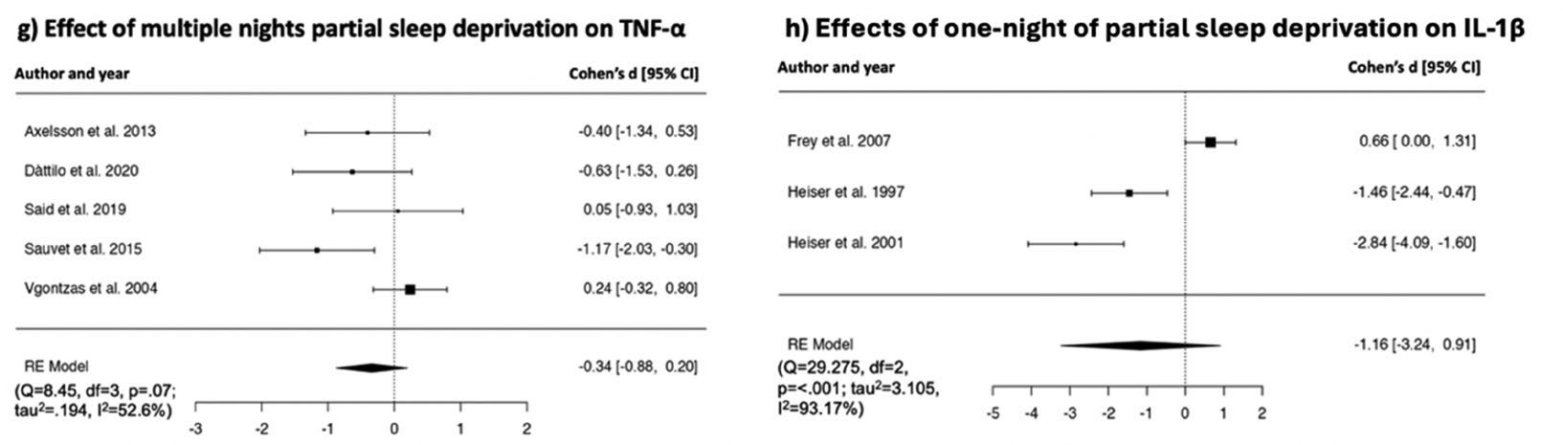
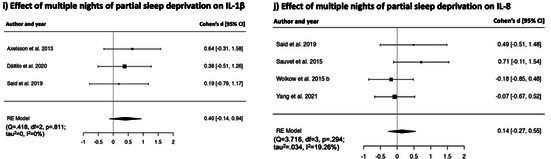

##### 3.4.1.2 Effects of One Night of Partial Sleep Deprivation on CRP

Preliminary random effect model revealed a non‐significant effect of one night of experimental partial sleep deprivation on CRP [*k* = 4, *d* = −0.47, [95% CI = −2.23 to 1.28], *p* = 0.595], with substantial heterogeneity (*Q* = 95.111, df = 3, *p* = < 0.001; *τ* ^2^ = 3.047, *I* ^2^ = 96.85%) and one outlier (Faraut et al. 2011, *d* = −4.07, 95% CI = −4.94 to 3.20). After excluding this study from the analysis, random effect model showed a non‐significant effect of one‐night of experimental partial sleep deprivation on CRP [*k* = 3, *d* = 0.58, [95% CI = −0.20 to 1.36], *p* = 0.147]. Heterogeneity statistics were still significant (*Q* = 10.518, df = 2, *p* = 0.005; *τ* ^2^ = 0.370, *I* ^2^ = 80.99%). Forest plot is reported in Table 2b.

##### 3.4.1.3 Effects of Multiple Nights of Partial Sleep Deprivation on CRP

Preliminary random effects model revealed a moderate non‐significant effect of multiple nights of experimental partial sleep deprivation on CRP [*k* = 6, *d* = 0.50, [95% CI = −0.38 to 1.38], *p* = 0.265], with substantial heterogeneity (Q = 36.65, df = 5, *p* < 0.001; *τ* ^2^ = 0.974, *I* ^2^ = 86.36%) and one potential outlier (Baek et al. 2020, *d* = −0.70, 95% CI = −0.97 to −0.44). After excluding this study from the analysis, random effects model showed a large significant effect of multiple nights of experimental partial sleep deprivation on CRP [*k* = 5, *d* = 0.76, [95% CI = 0.09 to 1.43], *p* = 0.027]. In the studies included in this analysis, sleep manipulation occurred over 7.5 ± 4.20 nights at 240 min. Heterogeneity was moderate (*Q* = 8.653, df = 4, *p* = 0.043; *τ* ^2^ = 0.308, *I* ^2^ = 53.77%). To investigate the potential sources of heterogeneity we considered study characteristics. One of the included studies adopted a between‐subjects design (Meier‐Ewert et al. 2004). We repeated the analysis excluding this study. Random effects model showed a non‐significant effect of multiple nights of experimental partial sleep deprivation on CRP for within‐subjects studies [*k* = 4, *d* = 0.61, [95% CI = −0.16 to 1.37], *p* = 0.12], and still moderate heterogeneity (*Q* = 6.544, df = 3, *p* = 0.088; *τ* ^2^ = 0.323, *I* ^2^ = 54.16%). Due to the small number of studies included in the analysis, we were not able to further examine the potential sources of heterogeneity. Forest plot is reported in Table 2c.

#### 3.4.2 IL‐6

##### 3.4.2.1 Effects of One Night of Total Sleep Deprivation on IL‐6

Preliminary random effect model revealed a non‐significant effect of one night of experimental total sleep deprivation on IL‐6 [*k* = 9, *d* = 0.41, [95% CI = −0.04 to 0.87], *p* = 0.076], with substantial heterogeneity (*Q* = 35.50, df = 8, < 0.001; *τ* ^2^ = 0.345, *I* ^2^ = 73.65%), and one potential outlier (Matsubara et al. 2023, *d* = 1.65, 95% CI = 1.05 to 2.26). After removing this study from the analysis, the random effect model showed a non‐significant effect of experimental total sleep deprivation on IL‐6 [*k* = 8, *d* = 0.21, [95% CI = −0.15 to 0.58], *p* = 0.251], and moderate heterogeneity (*Q* = 14.51, df = 7, = 0.043; *τ* ^2^ = 0.141, *I* ^2^ = 50.51%). Forest plot is reported in Table 2d.

##### 3.4.2.2 Effects of One Night of Partial Sleep Deprivation on IL‐6

Random effect model revealed a non‐significant effect of one night of experimental partial sleep deprivation on IL‐6 [*k* = 6, *d* = 0.48, [95% CI = −0.13 to 1.10], *p* = 0.125], and substantial heterogeneity (*Q* = 20.10, df = 5, < 0.001; *τ* ^2^ = 0.411, *I* ^2^ = 75.12%). Forest plot is reported in Table 2e.

##### 3.4.2.3 Effects of Multiple Nights of Partial Sleep Deprivation on IL‐6

Preliminary random effect model revealed a non‐significant effect of multiple nights of experimental partial sleep deprivation on IL‐6 [*k* = 6, *d* = 0.10, [95% CI = −0.48 to 0.67], *p* = 0.746], with substantial heterogeneity (*Q* = 18.71, df = 5, *p* < 0.001; *τ* ^2^ = 0.374, *I* ^2^ = 73.29%), and one outlier (Schmid et al. 2011, *d* = −1.38, 95% CI = −2.17 to −0.58). After excluding this study from the analysis, the random effect model showed a moderate significant effect of multiple nights of experimental partial sleep deprivation on IL‐6 [*k* = 5, *d* = 0.42, [95% CI = 0.11 to 0.73], *p* < 0.01]. Heterogeneity was low and non‐significant (*Q* = 1.80, df = 4, *p* = 0.772; *τ* ^2^ = 0.0, *I* ^2^ = 0.00%). In the studies included in this analysis, sleep manipulation occurred over 9.0 ± 7.00 nights at 276 ± 53 min. Forest plot is reported in Table 2f.

#### 3.4.3 TNF‐α

##### 3.4.3.1 Effects of Multiple Nights of Partial Sleep Deprivation on TNF‐α

Random effect model revealed a non‐significant effect of multiple nights of experimental partial sleep deprivation on TNF‐α [*k* = 5, *d* = −0.34, [95% CI = −0.88 to 0.20], *p* = 0.213], with non‐significant heterogeneity (*Q* = 8.45, df = 3, *p* = 0.07; *τ* ^2^ = 0.194, *I* ^2^ = 52.6%). Forest plot is reported in Table 2g.

#### 3.4.4 IL‐1β

##### 3.4.4.1 Effects of One Night of Total Sleep Deprivation on IL‐1β

Random effect model revealed a non‐significant effect of one night of experimental total sleep deprivation on IL‐1β [*k* = 3, *d* = −1.16, [95% CI = −3.24 to 0.91], *p* = 0.271], showing significant heterogeneity (*Q* = 29.275, df = 2, *p* = < 0.001; *τ* ^2^ = 3.105, *I* ^2^ = 93.17%). Forest plot is reported in Table 2h.

##### 3.4.4.2 Effects of Multiple Nights of Partial Sleep Deprivation on IL‐1β

Random effect model revealed a non‐significant effect of multiple nights of experimental partial sleep deprivation on IL‐1β [*k* = 3, *d* = 0.40, [95% CI = −0.14 to 0.94], *p* = 0.143], showing non‐significant heterogeneity (*Q* = 0.418, df = 2, *p* = 0.811; *τ* ^2^ = 0, *I* ^2^ = 0%). Forest plot is reported in Table 2i.

#### 3.4.5 IL‐8

##### 3.4.5.1 Effects of Multiple Nights of Partial Sleep Deprivation on IL‐8

Random effect model revealed a small non‐significant effect of multiple nights of experimental partial sleep deprivation on IL‐8 [*k* = 4, *d* = 0.14, [95% CI = −0.27 to 0.55], *p* = 0.496], with substantial heterogeneity (*Q* = 3.716, df = 3, *p* = 0.294; *τ* ^2^ = 0.034, *I* ^2^ = 19.26%). Forest plot is reported in Table 2j.

## 4 Discussion

This meta‐analysis found that partial sleep deprivation for multiple nights (8.3 ± 5.6 nights with sleep duration restricted to ~4.30 h) was associated with a significant increase in systemic markers of inflammation including IL‐6 and CRP, compared to normal sleep duration. This finding suggests that even mild, yet persistent sleep deprivation may activate inflammatory signalling pathways, therefore acting as a considerable challenge to the innate immune system. Putatively, the effects of partial sleep deprivation on IL‐6 and CRP may depend on classical mediators such as the nuclear factor κB transcriptional pathway as well as the hormone and growth factor response pathway as pointed out by previous experimental studies (Irwin et al. 2006, 2023). Interestingly, while cellular signals of inflammation (e.g., activated nuclear factor κB pathway, activator protein 1) may be activated by a single night of partial sleep deprivation (Irwin et al. 2006), our study suggests that the upregulation of inflammatory cytokines and acute‐phase proteins in blood may only occur after persistent periods of sleep deprivation.

IL‐6 is a multifunctional cytokine, physiologically involved in haematopoiesis and in the coordination of innate and adaptive immune functions including monocyte differentiation, antibody production from activated B cells, and promotion of type 2 helper T cell (Aliyu et al. 2022). The pathogenic role of IL‐6 has also been extensively described. IL‐6 is a major driver of CRP released by hepatocytes (Patel et al. 2007). The continuous over‐expression of IL‐6 results in hyper‐gammaglobulinemia and autoantibody production (Tanaka et al. 2014), so that dysregulation of IL‐6 is associated with multiple autoimmune diseases, including inflammatory bowel disease, rheumatoid arthritis, type 1 diabetes mellitus, and psoriasis (e.g., Male et al. 2006). IL‐6 is also considered a key regulator of metabolism and a growth factor involved in the development and maintenance of various cancers and cardiovascular disease (Male et al. 2006; Ho et al. 2015; Rose‐John 2020). Cardiovascular disease is the first cause of death in Europe (Townsend et al. 2022) and the USA (Ahmad and Anderson 2021). Epidemiological data show that short sleep duration (Oikonomou et al. 2021) and inflammation (Libby 2021) are involved in cardiovascular pathophysiology (e.g., atherosclerosis), with short sleep (Cappuccio et al. 2011; Wang et al. 2020), IL‐6, and CRP (Danesh et al. 2004; Feng et al. 2022) being moderate predictors of cardiovascular disease (Libby 2021; Oikonomou et al. 2021). Mediation studies further suggest that the association between short sleep and death by cardiovascular disease may partially be attributable to CRP (Gupta et al. 2021). More broadly, IL‐6 and CRP are associated with several hallmarks of disease including shortening telomere length (O'Donovan et al. 2011), mitochondrial dysfunction, and cellular senescence processes (Hoffman et al. 2023). Our meta‐analysis was based on healthy individuals. Therefore, it was not possible to directly investigate the role of sleep deprivation, IL‐6, and CRP on disease activity of specific clinical conditions. Future experimental studies may examine the potential mediating effect of IL‐6 and CRP between sleep deprivation and inflammation‐mediated pathophysiological processes.

IL‐6 and CRP are also longitudinally associated with increased risk of mental disorders which are robustly associated with short and disturbed sleep such as depression (Zhai et al. 2015). For instance, IL‐6 at 9 years old predicted the incidence of depression at age 24 in the ALSPAC cohort (Perry et al. 2021). Experimental endotoxemia (i.e., administration of purified bacterial endotoxin) is associated with peripheral and central nervous system cytokine upregulation including IL‐6 and consequent affective and physical symptoms of depression (e.g., Lasselin et al. 2021). Whether IL‐6 and CRP may mediate the association between sleep loss and the onset of depressive symptoms remains to be demonstrated (Ballesio 2023a, 2023b). In mice, sleep deprivation was associated with increased peripheral IL‐6 and neuroinflammation in the hippocampus and medial prefrontal cortex (Wang et al. 2021). Our findings suggest that multiple nights of partial sleep deprivation may serve as a valid experimental procedure to test the mediatory role of IL‐6 and CRP in the association between sleep loss and depression. Crucially, future studies are needed to explore whether IL‐6 and CRP following sleep deprivation may activate immune‐to‐brain pathways and inflammation within the central nervous system (e.g., microglia activation, cytokine released in cerebrospinal fluid).

Our results on IL‐6 are also consistent with large population longitudinal data suggesting that a decrease in sleep duration was associated with a 4.5% increase in IL‐6 over time (Ferrie et al. 2013). Moreover, our result to some extent aligns with meta‐analytic evidence on enhanced peripheral concentrations of IL‐6 and CRP in individuals with chronic insomnia (i.e., subjective difficulties in sleep onset and maintenance) compared to controls (Irwin et al. 2016; Zhang et al. 2023), and particularly with the literature on insomnia with concomitant objective short sleep duration (≤ 6 h) phenotype (Fernandez‐Mendoza et al. 2017). Indeed, insomnia is not necessarily characterised by objective short sleep duration, although a meta‐analysis of polysomnographic studies suggested a slightly shortened total sleep time in individuals with insomnia compared to good sleepers (Baglioni et al. 2014). It is possible that even a slight yet persistent decrease in sleep duration below the 7 h recommended for human adults (Hirshkowitz et al. 2015) may activate components of the innate immune system with a subsequent increase in systemic inflammatory markers. However, inflammation in chronic insomnia may also be the result of several factors other than sleep duration, including comorbidity and poor health behaviour (Ballesio 2023a, 2023b).

Notably, one night of sleep deprivation, either total or partial, did not significantly increase inflammation. This is consistent with an experimental mouse model of potential attenuation of inflammatory responses following acute sleep deprivation (Weil et al. 2009). A possible explanation is that acute sleep deprivation may result in hypothalamic–pituitary–adrenal (HPA) axis and sympathetic potentiation, with the consequent release of anti‐inflammatory glucocorticoids (e.g., Wright Jr et al. 2015). Instead, multiple nights of sleep deprivation may drive glucocorticoid resistance (i.e., reduced sensitivity of glucocorticoid receptors) and inflammation disinhibition (Miller et al. 2002). Finally, we found no significant evidence on the effects of sleep deprivation on IL‐1β, IL‐8, and TNF‐α, likely due to underpowered statistical analysis. We urge for the implementation of sleep deprivation studies of these markers in order to maximise the information on the impact of sleep on specific inflammatory processes.

### 4.1 Clinical Implications

If replicated in robust randomised controlled trials, our results would strengthen the need to include habitual sleep duration in the clinical assessment of individuals at risk of inflammation‐related conditions (e.g., autoimmune diseases, cardiovascular disease) and to target persistent short sleep duration with appropriate interventions. Daytime napping (e.g., 1 h at 13.00) and sleep extension intervention may be two strategies to reverse the effects of sleep deprivation on immune parameters in otherwise healthy individuals (Vgontzas et al. 2007; Faraut et al. 2011). In a recent a double‐blind randomised placebo‐controlled crossover trial, low dose acetylsalicylic acid reduced inflammation induced by partial sleep deprivation (Engert et al. 2024). Whether other low‐intensity strategies such as lifestyle intervention and mindfulness meditation with known efficacy in lowering IL‐6 and CRP (Ballesio et al. 2023) may buffer the effects of sleep deprivation on inflammation remains to be tested. In this regard, a recent randomised controlled trial suggested that tai chi movement meditation may reduce systemic, cellular, and genomic markers of inflammation in individuals with insomnia (Irwin et al. 2024).

### 4.2 Limitations

This meta‐analysis has several strengths, including the analysis of different types of sleep deprivation (one/multiple nights, partial/total), the assessment of multiple markers of inflammation, and the inclusion of 12 studies published after Irwin et al. (2016) meta‐analysis. A number of limitations should also be discussed. The sample size (*n* = 887) is relatively small. The dearth of studies examining the effects of experimental sleep deprivation is a problem for this field, which precludes the investigation of potential mediators. Several included studies were quasi‐experimental and lacked participant randomisation, which may potentially preclude a robust causal interpretation of the effects of sleep deprivation on inflammation. Well‐designed randomised controlled trials are therefore needed to corroborate our findings. The population under study was mostly composed of young adults. To potentiate the generalisability of results, future studies should be conducted in all‐ages samples. We were not able to examine the moderating role of biological sex, while females may be more vulnerable than males to detrimental effects of sleep loss and disordered sleep on immune parameters (Irwin et al. 2010; Ballesio et al. 2022; Ballesio et al. 2025). Females were under‐represented, and this may have impacted the magnitude of effects. Current evidence suggests that the association between short or disturbed sleep and inflammation may be stronger in females (Irwin 2019; Dolsen et al. 2019). For instance, Irwin et al. (2010) found a greater cellular immune activation following sleep deprivation compared to males. Similarly, the impact of disturbed sleep on inflammation may be more pronounced in females (Miller et al. 2009; Ballesio et al. 2025). Therefore, it is possible that the effects reported in the present meta‐analysis may underestimate the potential impact that sleep deprivation may have on females. To advance the field, future studies are recommended to report sex‐stratified analysis.

The analysis on CRP was based on a relatively limited set of studies, which precluded a deeper examination of the potential sources of heterogeneity. Meier‐Ewert et al. (2004) adopted a between‐subjects 10‐day partial sleep deprivation design and reported the largest effect size on CRP. After removing Meier‐Ewert et al. (2004) from the analysis, results were non‐significant. This may suggest a dose‐dependent effect of partial sleep deprivation: longer periods of partial sleep deprivation may have greater pro‐inflammatory effects. However, another between‐subjects study reported a small effect size and adopted a 12‐day design (Haack et al. 2007). Future evidence is needed to better define potential moderators of the association between sleep loss and CRP. Moreover, markers were assessed in the morning right after sleep manipulation, so the long‐term effects of sleep deprivation on inflammation are yet to be clarified by adopting a multiple assessments design. Following Cochrane's recommendations (Higgins and Green 2011), funnel plots for publication bias investigation, and related Egger's test for funnel plot asymmetry (Egger et al. 1997) were not employed due to the presence of fewer than 10 studies included in each analysis. Therefore, potential publication bias could not be excluded. As a further limitation of the current literature, BMI was reported by 14 out of 35 included studies. Since BMI can be strongly associated with inflammation (Zagaria et al. 2024), future studies are encouraged to assess and report BMI. Finally, we could not consider other inflammatory markers (e.g., soluble receptors) due to a lack of studies to suffice meta‐analysis.

## 5 Conclusions

This meta‐analysis of experimental and quasi‐experimental studies found that partial sleep deprivation of multiple nights was associated with increased blood concentrations of IL‐6 and CRP in healthy individuals. As aforementioned, results should be interpreted in light of the lack of randomisation in most studies. If replicated in robust randomised controlled trials, present results would suggest that partial sleep deprivation of at least 3 nights may serve as a valid procedure to elicit peripheral IL‐6 and CRP responses. Further research will be needed to understand the impact of sleep recovery in restoring inflammation homeostasis, as well as the role of neuroendocrine and autonomic factors.

## Author Contributions

**Andrea Ballesio:** conceptualization, investigation, writing – original draft, methodology, writing – review and editing, software, formal analysis, supervision, data curation. **Valeria Fiori:** writing – original draft, writing – review and editing, formal analysis, data curation. **Caterina Lombardo:** supervision, writing – review and editing.

## Conflicts of Interest

The authors declare no conflicts of interest.

## Supporting information


**Data S1** Supporting Information.

## Data Availability

The data that support the findings of this study are available from the corresponding author upon reasonable request.
