# Supplementary material for: Effects of Experimental Sleep Deprivation on Peripheral Inflammation: An Updated Meta‐Analysis of Human Studies
Source: J Sleep Res. 2025 Jun 5;35(1):e70099. doi: 10.1111/jsr.70099 (PMC12856123; doi:10.1111/jsr.70099)
Supplement: Supplementary file 1 — Data S1 Supporting Information. [file JSR-35-e70099-s001.docx]

**Supplemental Material**

1. **Detailed search string**

**Pubmed:**

("sleep deprivation"[Title/Abstract] OR "sleep restriction"[Title/Abstract] OR "sleep disruption"[Title/Abstract] OR "sleep curtailment"[Title/Abstract] OR "sleep loss"[Title/Abstract]) AND (cytokine*[Title/Abstract] OR chemokine*[Title/Abstract] OR interleukin*[Title/Abstract] OR IL-1[Title/Abstract] OR IL-2[Title/Abstract] OR IL-6[Title/Abstract] OR IL-10[Title/Abstract] OR interferon*[Title/Abstract] OR IFN[Title/Abstract] OR "tumour necrosis factor*"[Title/Abstract] OR "tumor necrosis factor*"[Title/Abstract] OR TNF[Title/Abstract] OR "C-Reactive Protein"[Title/Abstract] OR "C reactive protein"[Title/Abstract] OR CRP[Title/Abstract] OR hs-CRP[Title/Abstract])

**Scopus:**

( TITLE-ABS-KEY ( "sleep deprivation" OR "sleep restriction" OR "sleep disruption" OR "sleep curtailment" OR "sleep loss" ) AND TITLE-ABS-KEY ( cytokine* OR chemokine* OR interleukin* OR il-1 OR il-2 OR il-6 OR il-10 OR interferon* OR ifn OR "tumour necrosis factor*" OR "tumor necrosis factor*" OR tnf OR "c-reactive protein" OR "c reactive protein" OR crp OR hs-crp ) )

1. **Supplementary Table 1. Excluded studies and reasons for exclusion.**

| **Study** | **Reason for exclusion** |
| --- | --- |
| Aho et al. 2013 | no measure of inflammatory markers |
| Bollinger et al. 2009 | no data |
| Bollinger et al. 2010 | no data |
| Bottenhelf et al. 2023 | no data |
| Carroll et al. 2015 | no measure of inflammatory markers |
| Chen et al. 2016 | other manipulation* |
| Chen et al. 2023 | no measure of inflammatory markers |
| Chennaoui et al. 2014 | no measure of inflammatory markers |
| Chennaoui et al. 2017 | no measure of inflammatory markers |
| Dimitrov et al. 2004 | no measure of inflammatory markers |
| Dimitrov et al. 2015 | no data |
| Dutheil et al. 2013 | circadian misalignment |
| Foo et al. 2019 | no healthy participants |
| Goto et al. 2020 | no data |
| Haack et al. 2002 | no data |
| Hertel et al. 2024 | other manipulation |
| Hui et al. 2007 | no measure of inflammatory markers |
| Hunt et al. 2021 | no data |
| Irwin et al. 1999 | no data |
| Irwin et al. 2004 | no healthy participants |
| Irwin et al. 2006 | no measure of inflammatory markers |
| Irwin et al. 2008 | no measure of inflammatory markers |
| Irwin et al. 2010 | no measure of inflammatory markers |
| Irwin et al. 2015 | no measure of inflammatory markers |
| Irwin et al. 2023 | no measure of inflammatory markers |
| Khosro et al. 2011 | circadian misalignment |
| Kose Cinar et al. 2016 | no measure of inflammatory markers |
| Lekander et al. 2013 | no data |
| Li et al. 2018 | no human participants |
| Liu et al. 2021 | circadian misalignment |
| Liu et al. 2021 | other manipulation |
| Lorenzetti 2021 | grey literature |
| Moldofsky et al. 1989 | no data |
| Nakamura et al. 2013 | no healthy participants |
| Ness et al. 2019 | no data |
| Rahman et al. 2015 | no data |
| Reis et al. 2011 | no measure of inflammatory markers |
| Rodrigues et al. 2017 | other manipulation |
| Shearer et al. 2001 | other manipulation |
| Skein et al. 2013 | no data |
| Smith et al. 2018 | other manipulation |
| Taishi et al. 1998 | no human participants |
| Visalini et al. 2025 | no healthy participants |
| Voderholzer et al. 2012 | no healthy participants |
| Wolkow et al. 2015a | no data |
| Wolkow et al. 2016 | no data |
| Wolkow et al. 2016 | no data |
| Wright et al. 2015 | circadian misalignment |
| Xia et al. 2018 | no human participants |

*involve the concomitant manipulation of other variables during

sleep deprivation that do not allow to discriminate the effect of sleep

manipulation. For all the papers reporting no data, authors were contacted, but none responded.

1. **Supplementary Table 2. Results of the** **Downs and Black Quality Index scoring system (Downs & Black, 1998).**

| **Article Author & Name: Abedelmalek et al. 2013a** | **Unable to determine** | **no** | **yes** | **Notes/Justification** |
| --- | --- | --- | --- | --- |
| **Reporting** | 0 | 0 | 1 |  |
| *1. Is the hypothesis/aim/objective of the study clearly described?* |  |  | yes |  |
| *2. Are the main outcomes to be measured clearly described in the Introduction or Methods section?* If the main outcomes are first mentioned in the Results section, the question should be answered no |  |  | yes |  |
| *3. Are the characteristics of the patients included in the study clearly described ?* In cohort studies and trials, inclusion and/or exclusion criteria should be given. In case‐control studies, a case‐definition and the source for controls should be given. |  |  | yes |  |
| *4. Are the interventions of interest clearly described?*Treatments and placebo (where relevant) that are to be compared should be clearly described. |  |  | yes |  |
| *5. Are the distributions of principal confounders in each group of subjects to be compared clearly described?* A list of principal confounders is provided. | unable to determine |  |  |  |
| *6. Are the main findings of the study clearly described?* Simple outcome data (including denominators and numerators) should be reported for all major findings so that the reader can check the major analyses and conclusions. (This question does not cover statistical tests which are considered below). |  |  | yes |  |
| *7. Does the study provide estimates of the random variability in the data for the main outcomes?* In non normally distributed data the inter‐quartile range of results should be reported. In normally distributed data the standard error, standard deviation or confidence intervals should be reported. If the distribution of the data is not described, it must be assumed that the estimates used were appropriate and the question should be answered yes. |  |  | yes |  |
| *8. Have all important adverse events that may be a consequence of the intervention been reported?* This should be answered yes if the study demonstrates that there was a comprehensive attempt to measure adverse events. (A list of possible adverse events is provided). | unable to determine |  |  |  |
| *9. Have the characteristics of patients lost to follow‐up been described?* This should be answered yes where there were no losses to follow‐up or where losses to follow‐up were so small that findings would be unaffected by their inclusion. This should be answered no where a study does not report the number of patients lost to follow‐up. |  | no |  | The number of participants lost was not reported |
| *10. Have actual probability values been reported ( e.g. 0.035 rather than <0.05) for the main outcomes except where the probability value is less than 0.001?* |  | no |  |  |
| **External Validity** All the following criteria attempt to address the representativeness of the findings of the study and whether they may be generalised to the population from which the study subjects were derived. |  |  |  |  |
| *11. Were the subjects asked to participate in the study representative of the entire population from which they were recruited?* The study must identify the source population for patients and describe how the patients were selected. Patients would be representative if they comprised the entire source population, an unselected sample of consecutive patients, or a random sample. Random sampling is only feasible where a list of all members of the relevant population exists. Where a study does not report the proportion of the source population from which the patients are derived, the question should be answered as unable to determine. | unable to determine |  |  |  |
| *12. Were those subjects who were prepared to participate representative of the entire population from which they were recruited?*The proportion of those asked who agreed should be stated. Validation that the sample was representative would include demonstrating that the distribution of the main confounding factors was the same in the study sample and the source population. | unable to determine |  |  |  |
| *13. Were the staff, places, and facilities where the patients were treated, representative of the treatment the majority of patients receive?* For the question to be answered yes the study should demonstrate that the intervention was representative of that in use in the source population. The question should be answered no if, for example, the intervention was undertaken in a specialist centre unrepresentative of the hospitals most of the source population would attend. | unable to determine |  |  |  |
| **Internal validity – bias** |  |  |  |  |
| *14. Was an attempt made to blind study subjects to the intervention they have received?* For studies where the patients would have no way of knowing which  intervention they received, this should be answered yes. |  | no |  |  |
| *15. Was an attempt made to blind those measuring the main outcomes of the intervention?* |  | no |  |  |
| *16. If any of the results of the study were based on “data dredging”, was this made clear?* Any analyses that had not been planned at the outset of the study should be clearly indicated. If no retrospective unplanned subgroup analyses were reported, then answer yes. |  |  | yes |  |
| *17. In trials and cohort studies, do the analyses adjust for different lengths of follow‐up of patients, or in case‐control studies, is the time period between the intervention and outcome the same for cases and controls?*Where follow‐up was the same for all study patients the answer should yes. If different lengths of follow‐up were adjusted for by, for example, survival analysis the answer should be yes. Studies where differences in follow‐up are ignored should be answered no. |  |  | yes |  |
| 1*8. Were the statistical tests used to assess the main outcomes appropriate?* The statistical techniques used must be appropriate to the data. For example nonparametric methods should be used for small sample sizes. Where little statistical analysis has been undertaken but where there is no evidence of bias, the question should be answered yes. If the distribution of the data (normal or not) is not described it must be assumed that the estimates used were appropriate and the question should be answered yes. |  |  | yes |  |
| *19. Was compliance with the intervention/s reliable*? Where there was non compliance with the allocated treatment or where there was contamination of one group, the question should be answered no. For studies where the effect of any misclassification was likely to bias any association to the null, the question should be answered yes | unable to determine |  |  |  |
| *20. Were the main outcome measures used accurate (valid and reliable)?* For studies where the outcome measures are clearly described, the question should be answered yes. For studies which refer to other work or that demonstrates the outcome measures are accurate, the question should be answered as yes. |  |  | yes |  |
| **Internal validity ‐ confounding (selection bias)** |  |  |  |  |
| *21. Were the patients in different intervention groups (trials and cohort studies) or were the cases and controls (case‐control studies) recruited from the same population?* For example, patients for all comparison groups should be selected from the same hospital. The question should be answered unable to determine for cohort and casecontrol studies where there is no information concerning the source of patients included in the study. | unable to determine |  |  |  |
| *22. Were study subjects in different intervention groups (trials and cohort studies) or were the cases and controls (case‐control studies) recruited over the same period of time?* For a study which does not specify the time period over which patients were recruited, the question should be answered as unable to determine. | unable to determine |  |  |  |
| *23. Were study subjects randomised to intervention groups?* Studies which state that subjects were randomized should be answered yes except where method of randomisation would not ensure random allocation. For example alternate allocation would score no because it is predictable. |  |  | yes |  |
| *24. Was the randomised intervention assignment concealed from both patients and health care staff until recruitment was complete and irrevocable?* All non‐randomised studies should be answered no. If assignment was oncealed from patients but not from staff, it should be answered no. | unable to determine |  |  |  |
| *25. Was there adequate adjustment for confounding in the analyses from which the main findings were drawn?* This question should be answered no for trials if: the main conclusions of the study were based on analyses of treatment rather than intention to treat; the distribution of known confounders in the different treatment groups was not described; or the distribution of known confounders differed between the treatment groups but was not taken into account in the analyses. In nonrandomized studies if the effect of the main confounders was not investigated or confounding was demonstrated but no adjustment was made in the final analyses the question should be answered as no. |  | no |  |  |
| *26. Were losses of patients to follow‐up taken into account?* If the numbers of patients lost to follow‐up are not reported, the question should be answered as unable to determine. If the proportion lost to follow‐ up was too small to affect the main findings, the question should be answered yes. | unable to determine |  |  |  |
| **Power** |  |  |  |  |
| *27. Did the study have sufficient power to detect a clinically important effect where the probability value for a difference being due to chance is less than 5%?*Sample sizes have been calculated to detect a diVerence of x% and y%. |  | no |  | No a priori power analysis was conducted, unable to determine the power of the analysis of interests |
| **Total score:** |  |  | 11 |  |
|  |  |  |  |  |
| **Article Author & Name: Abedelmalek et al. 2013b** | unable to determine | no | yes | **Notes/Justification** |
| **Reporting** | 0 | 0 | 1 |  |
| *1. Is the hypothesis/aim/objective of the study clearly described?* |  |  | yes |  |
| *2. Are the main outcomes to be measured clearly described in the Introduction or Methods section?* If the main outcomes are first mentioned in the Results section, the question should be answered no |  |  | yes |  |
| *3. Are the characteristics of the patients included in the study clearly described ?* In cohort studies and trials, inclusion and/or exclusion criteria should be given. In case‐control studies, a case‐definition and the source for controls should be given. |  |  | yes |  |
| *4. Are the interventions of interest clearly described?*Treatments and placebo (where relevant) that are to be compared should be clearly described. |  |  | yes |  |
| *5. Are the distributions of principal confounders in each group of subjects to be compared clearly described?* A list of principal confounders is provided. | unable to determine |  |  |  |
| *6. Are the main findings of the study clearly described?* Simple outcome data (including denominators and numerators) should be reported for all major findings so that the reader can check the major analyses and conclusions. (This question does not cover statistical tests which are considered below). |  |  | yes |  |
| *7. Does the study provide estimates of the random variability in the data for the main outcomes?* In non normally distributed data the inter‐quartile range of results should be reported. In normally distributed data the standard error, standard deviation or confidence intervals should be reported. If the distribution of the data is not described, it must be assumed that the estimates used were appropriate and the question should be answered yes. |  |  | yes |  |
| *8. Have all important adverse events that may be a consequence of the intervention been reported?* This should be answered yes if the study demonstrates that there was a comprehensive attempt to measure adverse events. (A list of possible adverse events is provided). | unable to determine |  |  |  |
| *9. Have the characteristics of patients lost to follow‐up been described?* This should be answered yes where there were no losses to follow‐up or where losses to follow‐up were so small that findings would be unaffected by their inclusion. This should be answered no where a study does not report the number of patients lost to follow‐up. | unable to determine |  |  | The number of participants lost was not reported |
| *10. Have actual probability values been reported ( e.g. 0.035 rather than <0.05) for the main outcomes except where the probability value is less than 0.001?* |  |  | no |  |
| **External Validity** All the following criteria attempt to address the representativeness of the findings of the study and whether they may be generalised to the population from which the study subjects were derived. |  |  |  |  |
| *11. Were the subjects asked to participate in the study representative of the entire population from which they were recruited?* The study must identify the source population for patients and describe how the patients were selected. Patients would be representative if they comprised the entire source population, an unselected sample of consecutive patients, or a random sample. Random sampling is only feasible where a list of all members of the relevant population exists. Where a study does not report the proportion of the source population from which the patients are derived, the question should be answered as unable to determine. | unable to determine |  |  |  |
| *12. Were those subjects who were prepared to participate representative of the entire population from which they were recruited?*The proportion of those asked who agreed should be stated. Validation that the sample was representative would include demonstrating that the distribution of the main confounding factors was the same in the study sample and the source population. | unable to determine |  |  |  |
| *13. Were the staff, places, and facilities where the patients were treated, representative of the treatment the majority of patients receive?* For the question to be answered yes the study should demonstrate that the intervention was representative of that in use in the source population. The question should be answered no if, for example, the intervention was undertaken in a specialist centre unrepresentative of the hospitals most of the source population would attend. | unable to determine |  |  |  |
| **Internal validity – bias** |  |  |  |  |
| *14. Was an attempt made to blind study subjects to the intervention they have received?* For studies where the patients would have no way of knowing which  intervention they received, this should be answered yes. |  |  | no |  |
| *15. Was an attempt made to blind those measuring the main outcomes of the intervention?* | unable to determine |  |  |  |
| *16. If any of the results of the study were based on “data dredging”, was this made clear?* Any analyses that had not been planned at the outset of the study should be clearly indicated. If no retrospective unplanned subgroup analyses were reported, then answer yes. |  |  | yes |  |
| *17. In trials and cohort studies, do the analyses adjust for different lengths of follow‐up of patients, or in case‐control studies, is the time period between the intervention and outcome the same for cases and controls?*Where follow‐up was the same for all study patients the answer should yes. If different lengths of follow‐up were adjusted for by, for example, survival analysis the answer should be yes. Studies where differences in follow‐up are ignored should be answered no. | unable to determine |  |  |  |
| 1*8. Were the statistical tests used to assess the main outcomes appropriate?* The statistical techniques used must be appropriate to the data. For example nonparametric methods should be used for small sample sizes. Where little statistical analysis has been undertaken but where there is no evidence of bias, the question should be answered yes. If the distribution of the data (normal or not) is not described it must be assumed that the estimates used were appropriate and the question should be answered yes. |  |  | yes |  |
| *19. Was compliance with the intervention/s reliable*? Where there was non compliance with the allocated treatment or where there was contamination of one group, the question should be answered no. For studies where the effect of any misclassification was likely to bias any association to the null, the question should be answered yes |  |  | yes |  |
| *20. Were the main outcome measures used accurate (valid and reliable)?* For studies where the outcome measures are clearly described, the question should be answered yes. For studies which refer to other work or that demonstrates the outcome measures are accurate, the question should be answered as yes. |  |  | yes |  |
| **Internal validity ‐ confounding (selection bias)** |  |  |  |  |
| *21. Were the patients in different intervention groups (trials and cohort studies) or were the cases and controls (case‐control studies) recruited from the same population?* For example, patients for all comparison groups should be selected from the same hospital. The question should be answered unable to determine for cohort and casecontrol studies where there is no information concerning the source of patients included in the study. |  |  | yes |  |
| *22. Were study subjects in different intervention groups (trials and cohort studies) or were the cases and controls (case‐control studies) recruited over the same period of time?* For a study which does not specify the time period over which patients were recruited, the question should be answered as unable to determine. | unable to determine |  |  |  |
| *23. Were study subjects randomised to intervention groups?* Studies which state that subjects were randomized should be answered yes except where method of randomisation would not ensure random allocation. For example alternate allocation would score no because it is predictable. |  | no |  |  |
| *24. Was the randomised intervention assignment concealed from both patients and health care staff until recruitment was complete and irrevocable?* All non‐randomised studies should be answered no. If assignment was oncealed from patients but not from staff, it should be answered no. | unable to determine |  |  |  |
| *25. Was there adequate adjustment for confounding in the analyses from which the main findings were drawn?* This question should be answered no for trials if: the main conclusions of the study were based on analyses of treatment rather than intention to treat; the distribution of known confounders in the different treatment groups was not described; or the distribution of known confounders differed between the treatment groups but was not taken into account in the analyses. In nonrandomized studies if the effect of the main confounders was not investigated or confounding was demonstrated but no adjustment was made in the final analyses the question should be answered as no. |  | no |  |  |
| *26. Were losses of patients to follow‐up taken into account?* If the numbers of patients lost to follow‐up are not reported, the question should be answered as unable to determine. If the proportion lost to follow‐ up was too small to affect the main findings, the question should be answered yes. | unable to dtermine |  |  |  |
| **Power** |  |  |  |  |
| *27. Did the study have sufficient power to detect a clinically important effect where the probability value for a difference being due to chance is less than 5%?*Sample sizes have been calculated to detect a diVerence of x% and y%. |  | no |  | No a priori analysis was conducted, adequate effect size |
| **Total score:** |  |  | 11 |  |
|  |  |  |  |  |
| **Article Author & Name: Axelsson et al. 2013** | unable to determine | no | yes | **Notes/Justification** |
| **Reporting** | 0 | 0 | 1 |  |
| *1. Is the hypothesis/aim/objective of the study clearly described?* |  |  | yes |  |
| *2. Are the main outcomes to be measured clearly described in the Introduction or Methods section?* If the main outcomes are first mentioned in the Results section, the question should be answered no |  |  | yes |  |
| *3. Are the characteristics of the patients included in the study clearly described ?* In cohort studies and trials, inclusion and/or exclusion criteria should be given. In case‐control studies, a case‐definition and the source for controls should be given. |  |  | yes |  |
| *4. Are the interventions of interest clearly described?*Treatments and placebo (where relevant) that are to be compared should be clearly described. |  |  | yes |  |
| *5. Are the distributions of principal confounders in each group of subjects to be compared clearly described?* A list of principal confounders is provided. | unable to determine |  |  |  |
| *6. Are the main findings of the study clearly described?* Simple outcome data (including denominators and numerators) should be reported for all major findings so that the reader can check the major analyses and conclusions. (This question does not cover statistical tests which are considered below). |  |  | yes |  |
| *7. Does the study provide estimates of the random variability in the data for the main outcomes?* In non normally distributed data the inter‐quartile range of results should be reported. In normally distributed data the standard error, standard deviation or confidence intervals should be reported. If the distribution of the data is not described, it must be assumed that the estimates used were appropriate and the question should be answered yes. |  |  | yes |  |
| *8. Have all important adverse events that may be a consequence of the intervention been reported?* This should be answered yes if the study demonstrates that there was a comprehensive attempt to measure adverse events. (A list of possible adverse events is provided). | unable to determine |  |  |  |
| *9. Have the characteristics of patients lost to follow‐up been described?* This should be answered yes where there were no losses to follow‐up or where losses to follow‐up were so small that findings would be unaffected by their inclusion. This should be answered no where a study does not report the number of patients lost to follow‐up. | unable to determine |  |  | The number of participants lost was not reported |
| *10. Have actual probability values been reported ( e.g. 0.035 rather than <0.05) for the main outcomes except where the probability value is less than 0.001?* |  | no |  |  |
| **External Validity** All the following criteria attempt to address the representativeness of the findings of the study and whether they may be generalised to the population from which the study subjects were derived. |  |  |  |  |
| *11. Were the subjects asked to participate in the study representative of the entire population from which they were recruited?* The study must identify the source population for patients and describe how the patients were selected. Patients would be representative if they comprised the entire source population, an unselected sample of consecutive patients, or a random sample. Random sampling is only feasible where a list of all members of the relevant population exists. Where a study does not report the proportion of the source population from which the patients are derived, the question should be answered as unable to determine. | unable to determine |  |  |  |
| *12. Were those subjects who were prepared to participate representative of the entire population from which they were recruited?*The proportion of those asked who agreed should be stated. Validation that the sample was representative would include demonstrating that the distribution of the main confounding factors was the same in the study sample and the source population. | unable to determine |  |  |  |
| *13. Were the staff, places, and facilities where the patients were treated, representative of the treatment the majority of patients receive?* For the question to be answered yes the study should demonstrate that the intervention was representative of that in use in the source population. The question should be answered no if, for example, the intervention was undertaken in a specialist centre unrepresentative of the hospitals most of the source population would attend. |  | no |  |  |
| **Internal validity – bias** |  |  |  |  |
| *14. Was an attempt made to blind study subjects to the intervention they have received?* For studies where the patients would have no way of knowing which  intervention they received, this should be answered yes. |  | no |  |  |
| *15. Was an attempt made to blind those measuring the main outcomes of the intervention?* |  | no |  |  |
| *16. If any of the results of the study were based on “data dredging”, was this made clear?* Any analyses that had not been planned at the outset of the study should be clearly indicated. If no retrospective unplanned subgroup analyses were reported, then answer yes. |  |  | yes |  |
| *17. In trials and cohort studies, do the analyses adjust for different lengths of follow‐up of patients, or in case‐control studies, is the time period between the intervention and outcome the same for cases and controls?*Where follow‐up was the same for all study patients the answer should yes. If different lengths of follow‐up were adjusted for by, for example, survival analysis the answer should be yes. Studies where differences in follow‐up are ignored should be answered no. | unable to determine |  |  |  |
| 1*8. Were the statistical tests used to assess the main outcomes appropriate?* The statistical techniques used must be appropriate to the data. For example nonparametric methods should be used for small sample sizes. Where little statistical analysis has been undertaken but where there is no evidence of bias, the question should be answered yes. If the distribution of the data (normal or not) is not described it must be assumed that the estimates used were appropriate and the question should be answered yes. |  |  | yes |  |
| *19. Was compliance with the intervention/s reliable*? Where there was non compliance with the allocated treatment or where there was contamination of one group, the question should be answered no. For studies where the effect of any misclassification was likely to bias any association to the null, the question should be answered yes |  |  | yes |  |
| *20. Were the main outcome measures used accurate (valid and reliable)?* For studies where the outcome measures are clearly described, the question should be answered yes. For studies which refer to other work or that demonstrates the outcome measures are accurate, the question should be answered as yes. |  |  | yes |  |
| **Internal validity ‐ confounding (selection bias)** |  |  |  |  |
| *21. Were the patients in different intervention groups (trials and cohort studies) or were the cases and controls (case‐control studies) recruited from the same population?* For example, patients for all comparison groups should be selected from the same hospital. The question should be answered unable to determine for cohort and casecontrol studies where there is no information concerning the source of patients included in the study. | unable to determine |  |  |  |
| *22. Were study subjects in different intervention groups (trials and cohort studies) or were the cases and controls (case‐control studies) recruited over the same period of time?* For a study which does not specify the time period over which patients were recruited, the question should be answered as unable to determine. | unable to determine |  |  |  |
| *23. Were study subjects randomised to intervention groups?* Studies which state that subjects were randomized should be answered yes except where method of randomisation would not ensure random allocation. For example alternate allocation would score no because it is predictable. | unable to determine |  |  |  |
| *24. Was the randomised intervention assignment concealed from both patients and health care staff until recruitment was complete and irrevocable?* All non‐randomised studies should be answered no. If assignment was oncealed from patients but not from staff, it should be answered no. | unable to determine |  |  |  |
| *25. Was there adequate adjustment for confounding in the analyses from which the main findings were drawn?* This question should be answered no for trials if: the main conclusions of the study were based on analyses of treatment rather than intention to treat; the distribution of known confounders in the different treatment groups was not described; or the distribution of known confounders differed between the treatment groups but was not taken into account in the analyses. In nonrandomized studies if the effect of the main confounders was not investigated or confounding was demonstrated but no adjustment was made in the final analyses the question should be answered as no. |  | no |  |  |
| *26. Were losses of patients to follow‐up taken into account?* If the numbers of patients lost to follow‐up are not reported, the question should be answered as unable to determine. If the proportion lost to follow‐ up was too small to affect the main findings, the question should be answered yes. | unable to determine |  |  |  |
| **Power** |  |  |  |  |
| *27. Did the study have sufficient power to detect a clinically important effect where the probability value for a difference being due to chance is less than 5%?*Sample sizes have been calculated to detect a difference of x% and y%. |  | no |  | no a priori power analysis was conducted, unable to determine effect sezes of interested outcome |
| **Total score:** |  |  | 10 |  |
|  |  |  |  |  |
| **Article Author & Name: Baek et al. 2020** | unable to determine | no | yes | **Notes/Justification** |
| **Reporting** | 0 | 0 | 1 |  |
| *1. Is the hypothesis/aim/objective of the study clearly described?* |  |  | yes |  |
| *2. Are the main outcomes to be measured clearly described in the Introduction or Methods section?* If the main outcomes are first mentioned in the Results section, the question should be answered no |  |  | yes |  |
| *3. Are the characteristics of the patients included in the study clearly described ?* In cohort studies and trials, inclusion and/or exclusion criteria should be given. In case‐control studies, a case‐definition and the source for controls should be given. |  |  | yes |  |
| *4. Are the interventions of interest clearly described?*Treatments and placebo (where relevant) that are to be compared should be clearly described. |  |  | yes |  |
| *5. Are the distributions of principal confounders in each group of subjects to be compared clearly described?* A list of principal confounders is provided. |  | no |  |  |
| *6. Are the main findings of the study clearly described?* Simple outcome data (including denominators and numerators) should be reported for all major findings so that the reader can check the major analyses and conclusions. (This question does not cover statistical tests which are considered below). |  |  | yes |  |
| *7. Does the study provide estimates of the random variability in the data for the main outcomes?* In non normally distributed data the inter‐quartile range of results should be reported. In normally distributed data the standard error, standard deviation or confidence intervals should be reported. If the distribution of the data is not described, it must be assumed that the estimates used were appropriate and the question should be answered yes. |  |  | yes |  |
| *8. Have all important adverse events that may be a consequence of the intervention been reported?* This should be answered yes if the study demonstrates that there was a comprehensive attempt to measure adverse events. (A list of possible adverse events is provided). |  | no |  |  |
| *9. Have the characteristics of patients lost to follow‐up been described?* This should be answered yes where there were no losses to follow‐up or where losses to follow‐up were so small that findings would be unaffected by their inclusion. This should be answered no where a study does not report the number of patients lost to follow‐up. |  | no |  |  |
| *10. Have actual probability values been reported ( e.g. 0.035 rather than <0.05) for the main outcomes except where the probability value is less than 0.001?* |  |  | yes |  |
| **External Validity** All the following criteria attempt to address the representativeness of the findings of the study and whether they may be generalised to the population from which the study subjects were derived. |  |  |  |  |
| *11. Were the subjects asked to participate in the study representative of the entire population from which they were recruited?* The study must identify the source population for patients and describe how the patients were selected. Patients would be representative if they comprised the entire source population, an unselected sample of consecutive patients, or a random sample. Random sampling is only feasible where a list of all members of the relevant population exists. Where a study does not report the proportion of the source population from which the patients are derived, the question should be answered as unable to determine. | unable to determine |  |  |  |
| *12. Were those subjects who were prepared to participate representative of the entire population from which they were recruited?*The proportion of those asked who agreed should be stated. Validation that the sample was representative would include demonstrating that the distribution of the main confounding factors was the same in the study sample and the source population. | unable to determine |  |  |  |
| *13. Were the staff, places, and facilities where the patients were treated, representative of the treatment the majority of patients receive?* For the question to be answered yes the study should demonstrate that the intervention was representative of that in use in the source population. The question should be answered no if, for example, the intervention was undertaken in a specialist centre unrepresentative of the hospitals most of the source population would attend. |  |  | yes |  |
| **Internal validity – bias** |  |  |  |  |
| *14. Was an attempt made to blind study subjects to the intervention they have received?* For studies where the patients would have no way of knowing which  intervention they received, this should be answered yes. |  | no |  |  |
| *15. Was an attempt made to blind those measuring the main outcomes of the intervention?* |  | no |  |  |
| *16. If any of the results of the study were based on “data dredging”, was this made clear?* Any analyses that had not been planned at the outset of the study should be clearly indicated. If no retrospective unplanned subgroup analyses were reported, then answer yes. |  |  | yes |  |
| *17. In trials and cohort studies, do the analyses adjust for different lengths of follow‐up of patients, or in case‐control studies, is the time period between the intervention and outcome the same for cases and controls?*Where follow‐up was the same for all study patients the answer should yes. If different lengths of follow‐up were adjusted for by, for example, survival analysis the answer should be yes. Studies where differences in follow‐up are ignored should be answered no. |  |  | yes |  |
| 1*8. Were the statistical tests used to assess the main outcomes appropriate?* The statistical techniques used must be appropriate to the data. For example nonparametric methods should be used for small sample sizes. Where little statistical analysis has been undertaken but where there is no evidence of bias, the question should be answered yes. If the distribution of the data (normal or not) is not described it must be assumed that the estimates used were appropriate and the question should be answered yes. |  |  | yes |  |
| *19. Was compliance with the intervention/s reliable*? Where there was non compliance with the allocated treatment or where there was contamination of one group, the question should be answered no. For studies where the effect of any misclassification was likely to bias any association to the null, the question should be answered yes | unable to determine |  |  |  |
| *20. Were the main outcome measures used accurate (valid and reliable)?* For studies where the outcome measures are clearly described, the question should be answered yes. For studies which refer to other work or that demonstrates the outcome measures are accurate, the question should be answered as yes. |  |  | yes |  |
| **Internal validity ‐ confounding (selection bias)** |  |  |  |  |
| *21. Were the patients in different intervention groups (trials and cohort studies) or were the cases and controls (case‐control studies) recruited from the same population?* For example, patients for all comparison groups should be selected from the same hospital. The question should be answered unable to determine for cohort and casecontrol studies where there is no information concerning the source of patients included in the study. | unable to determine |  |  |  |
| *22. Were study subjects in different intervention groups (trials and cohort studies) or were the cases and controls (case‐control studies) recruited over the same period of time?* For a study which does not specify the time period over which patients were recruited, the question should be answered as unable to determine. |  |  | yes |  |
| *23. Were study subjects randomised to intervention groups?* Studies which state that subjects were randomized should be answered yes except where method of randomisation would not ensure random allocation. For example alternate allocation would score no because it is predictable. |  | no |  |  |
| *24. Was the randomised intervention assignment concealed from both patients and health care staff until recruitment was complete and irrevocable?* All non‐randomised studies should be answered no. If assignment was oncealed from patients but not from staff, it should be answered no. | unable to deteermine |  |  |  |
| *25. Was there adequate adjustment for confounding in the analyses from which the main findings were drawn?* This question should be answered no for trials if: the main conclusions of the study were based on analyses of treatment rather than intention to treat; the distribution of known confounders in the different treatment groups was not described; or the distribution of known confounders differed between the treatment groups but was not taken into account in the analyses. In nonrandomized studies if the effect of the main confounders was not investigated or confounding was demonstrated but no adjustment was made in the final analyses the question should be answered as no. |  | no |  |  |
| *26. Were losses of patients to follow‐up taken into account?* If the numbers of patients lost to follow‐up are not reported, the question should be answered as unable to determine. If the proportion lost to follow‐ up was too small to affect the main findings, the question should be answered yes. |  |  | yes |  |
| **Power** |  |  |  |  |
| *27. Did the study have sufficient power to detect a clinically important effect where the probability value for a difference being due to chance is less than 5%?*Sample sizes have been calculated to detect a difference of x% and y%. |  |  | yes |  |
| **Total score:** |  |  | 15 |  |
|  |  |  |  |  |
| **Article Author & Name: Barragan et al. 2023** | unable to determine | no | yes | **Notes/Justification** |
| **Reporting** | 0 | 0 | 1 |  |
| *1. Is the hypothesis/aim/objective of the study clearly described?* |  |  | yes |  |
| *2. Are the main outcomes to be measured clearly described in the Introduction or Methods section?* If the main outcomes are first mentioned in the Results section, the question should be answered no |  |  | yes |  |
| *3. Are the characteristics of the patients included in the study clearly described ?* In cohort studies and trials, inclusion and/or exclusion criteria should be given. In case‐control studies, a case‐definition and the source for controls should be given. |  |  | yes |  |
| *4. Are the interventions of interest clearly described?*Treatments and placebo (where relevant) that are to be compared should be clearly described. |  |  | yes |  |
| *5. Are the distributions of principal confounders in each group of subjects to be compared clearly described?* A list of principal confounders is provided. |  |  | yes (2) |  |
| *6. Are the main findings of the study clearly described?* Simple outcome data (including denominators and numerators) should be reported for all major findings so that the reader can check the major analyses and conclusions. (This question does not cover statistical tests which are considered below). |  |  | yes |  |
| *7. Does the study provide estimates of the random variability in the data for the main outcomes?* In non normally distributed data the inter‐quartile range of results should be reported. In normally distributed data the standard error, standard deviation or confidence intervals should be reported. If the distribution of the data is not described, it must be assumed that the estimates used were appropriate and the question should be answered yes. |  |  | yes |  |
| *8. Have all important adverse events that may be a consequence of the intervention been reported?* This should be answered yes if the study demonstrates that there was a comprehensive attempt to measure adverse events. (A list of possible adverse events is provided). | unable to determine |  |  |  |
| *9. Have the characteristics of patients lost to follow‐up been described?* This should be answered yes where there were no losses to follow‐up or where losses to follow‐up were so small that findings would be unaffected by their inclusion. This should be answered no where a study does not report the number of patients lost to follow‐up. |  | no |  |  |
| *10. Have actual probability values been reported ( e.g. 0.035 rather than <0.05) for the main outcomes except where the probability value is less than 0.001?* |  |  | yes |  |
| **External Validity** All the following criteria attempt to address the representativeness of the findings of the study and whether they may be generalised to the population from which the study subjects were derived. |  |  |  |  |
| *11. Were the subjects asked to participate in the study representative of the entire population from which they were recruited?* The study must identify the source population for patients and describe how the patients were selected. Patients would be representative if they comprised the entire source population, an unselected sample of consecutive patients, or a random sample. Random sampling is only feasible where a list of all members of the relevant population exists. Where a study does not report the proportion of the source population from which the patients are derived, the question should be answered as unable to determine. | unable to determine |  |  |  |
| *12. Were those subjects who were prepared to participate representative of the entire population from which they were recruited?*The proportion of those asked who agreed should be stated. Validation that the sample was representative would include demonstrating that the distribution of the main confounding factors was the same in the study sample and the source population. |  |  | yes |  |
| *13. Were the staff, places, and facilities where the patients were treated, representative of the treatment the majority of patients receive?* For the question to be answered yes the study should demonstrate that the intervention was representative of that in use in the source population. The question should be answered no if, for example, the intervention was undertaken in a specialist centre unrepresentative of the hospitals most of the source population would attend. | unable to determine |  |  |  |
| **Internal validity – bias** |  |  |  |  |
| *14. Was an attempt made to blind study subjects to the intervention they have received?* For studies where the patients would have no way of knowing which  intervention they received, this should be answered yes. |  | no |  |  |
| *15. Was an attempt made to blind those measuring the main outcomes of the intervention?* |  | no |  |  |
| *16. If any of the results of the study were based on “data dredging”, was this made clear?* Any analyses that had not been planned at the outset of the study should be clearly indicated. If no retrospective unplanned subgroup analyses were reported, then answer yes. |  |  | yes |  |
| *17. In trials and cohort studies, do the analyses adjust for different lengths of follow‐up of patients, or in case‐control studies, is the time period between the intervention and outcome the same for cases and controls?*Where follow‐up was the same for all study patients the answer should yes. If different lengths of follow‐up were adjusted for by, for example, survival analysis the answer should be yes. Studies where differences in follow‐up are ignored should be answered no. |  |  | yes |  |
| 1*8. Were the statistical tests used to assess the main outcomes appropriate?* The statistical techniques used must be appropriate to the data. For example nonparametric methods should be used for small sample sizes. Where little statistical analysis has been undertaken but where there is no evidence of bias, the question should be answered yes. If the distribution of the data (normal or not) is not described it must be assumed that the estimates used were appropriate and the question should be answered yes. |  |  | yes |  |
| *19. Was compliance with the intervention/s reliable*? Where there was non compliance with the allocated treatment or where there was contamination of one group, the question should be answered no. For studies where the effect of any misclassification was likely to bias any association to the null, the question should be answered yes |  |  | yes |  |
| *20. Were the main outcome measures used accurate (valid and reliable)?* For studies where the outcome measures are clearly described, the question should be answered yes. For studies which refer to other work or that demonstrates the outcome measures are accurate, the question should be answered as yes. |  |  | yes |  |
| **Internal validity ‐ confounding (selection bias)** |  |  |  |  |
| *21. Were the patients in different intervention groups (trials and cohort studies) or were the cases and controls (case‐control studies) recruited from the same population?* For example, patients for all comparison groups should be selected from the same hospital. The question should be answered unable to determine for cohort and casecontrol studies where there is no information concerning the source of patients included in the study. | unable to determine |  |  |  |
| *22. Were study subjects in different intervention groups (trials and cohort studies) or were the cases and controls (case‐control studies) recruited over the same period of time?* For a study which does not specify the time period over which patients were recruited, the question should be answered as unable to determine. |  |  | yes |  |
| *23. Were study subjects randomised to intervention groups?* Studies which state that subjects were randomized should be answered yes except where method of randomisation would not ensure random allocation. For example alternate allocation would score no because it is predictable. |  |  | yes |  |
| *24. Was the randomised intervention assignment concealed from both patients and health care staff until recruitment was complete and irrevocable?* All non‐randomised studies should be answered no. If assignment was oncealed from patients but not from staff, it should be answered no. | unable to determine |  |  |  |
| *25. Was there adequate adjustment for confounding in the analyses from which the main findings were drawn?* This question should be answered no for trials if: the main conclusions of the study were based on analyses of treatment rather than intention to treat; the distribution of known confounders in the different treatment groups was not described; or the distribution of known confounders differed between the treatment groups but was not taken into account in the analyses. In nonrandomized studies if the effect of the main confounders was not investigated or confounding was demonstrated but no adjustment was made in the final analyses the question should be answered as no. |  | no |  |  |
| *26. Were losses of patients to follow‐up taken into account?* If the numbers of patients lost to follow‐up are not reported, the question should be answered as unable to determine. If the proportion lost to follow‐ up was too small to affect the main findings, the question should be answered yes. |  |  | yes |  |
| **Power** |  |  |  |  |
| *27. Did the study have sufficient power to detect a clinically important effect where the probability value for a difference being due to chance is less than 5%?*Sample sizes have been calculated to detect a difference of x% and y%. |  | no |  | no a priori power analysis was conducted, small effect size |
| **Total score:** |  |  | 18 |  |
|  |  |  |  |  |
| **Article Author & Name: Benedict et al. 2007** | unable to determine | no | yes | **Notes/Justification** |
| **Reporting** | 0 | 0 | 1 |  |
| *1. Is the hypothesis/aim/objective of the study clearly described?* |  |  | yes |  |
| *2. Are the main outcomes to be measured clearly described in the Introduction or Methods section?* If the main outcomes are first mentioned in the Results section, the question should be answered no |  |  | yes |  |
| *3. Are the characteristics of the patients included in the study clearly described ?* In cohort studies and trials, inclusion and/or exclusion criteria should be given. In case‐control studies, a case‐definition and the source for controls should be given. |  |  | yes |  |
| *4. Are the interventions of interest clearly described?*Treatments and placebo (where relevant) that are to be compared should be clearly described. |  |  | yes |  |
| *5. Are the distributions of principal confounders in each group of subjects to be compared clearly described?* A list of principal confounders is provided. | unable to determine |  |  |  |
| *6. Are the main findings of the study clearly described?* Simple outcome data (including denominators and numerators) should be reported for all major findings so that the reader can check the major analyses and conclusions. (This question does not cover statistical tests which are considered below). |  |  | yes |  |
| *7. Does the study provide estimates of the random variability in the data for the main outcomes?* In non normally distributed data the inter‐quartile range of results should be reported. In normally distributed data the standard error, standard deviation or confidence intervals should be reported. If the distribution of the data is not described, it must be assumed that the estimates used were appropriate and the question should be answered yes. |  |  | yes |  |
| *8. Have all important adverse events that may be a consequence of the intervention been reported?* This should be answered yes if the study demonstrates that there was a comprehensive attempt to measure adverse events. (A list of possible adverse events is provided). |  | no |  |  |
| *9. Have the characteristics of patients lost to follow‐up been described?* This should be answered yes where there were no losses to follow‐up or where losses to follow‐up were so small that findings would be unaffected by their inclusion. This should be answered no where a study does not report the number of patients lost to follow‐up. |  | no |  |  |
| *10. Have actual probability values been reported ( e.g. 0.035 rather than <0.05) for the main outcomes except where the probability value is less than 0.001?* |  |  | yes |  |
| **External Validity** All the following criteria attempt to address the representativeness of the findings of the study and whether they may be generalised to the population from which the study subjects were derived. |  |  |  |  |
| *11. Were the subjects asked to participate in the study representative of the entire population from which they were recruited?* The study must identify the source population for patients and describe how the patients were selected. Patients would be representative if they comprised the entire source population, an unselected sample of consecutive patients, or a random sample. Random sampling is only feasible where a list of all members of the relevant population exists. Where a study does not report the proportion of the source population from which the patients are derived, the question should be answered as unable to determine. | unable to determine |  |  |  |
| *12. Were those subjects who were prepared to participate representative of the entire population from which they were recruited?*The proportion of those asked who agreed should be stated. Validation that the sample was representative would include demonstrating that the distribution of the main confounding factors was the same in the study sample and the source population. | unable to determine |  |  |  |
| *13. Were the staff, places, and facilities where the patients were treated, representative of the treatment the majority of patients receive?* For the question to be answered yes the study should demonstrate that the intervention was representative of that in use in the source population. The question should be answered no if, for example, the intervention was undertaken in a specialist centre unrepresentative of the hospitals most of the source population would attend. | unable to determine |  |  |  |
| **Internal validity – bias** |  |  |  |  |
| *14. Was an attempt made to blind study subjects to the intervention they have received?* For studies where the patients would have no way of knowing which  intervention they received, this should be answered yes. |  | no |  |  |
| *15. Was an attempt made to blind those measuring the main outcomes of the intervention?* |  | no |  |  |
| *16. If any of the results of the study were based on “data dredging”, was this made clear?* Any analyses that had not been planned at the outset of the study should be clearly indicated. If no retrospective unplanned subgroup analyses were reported, then answer yes. |  |  | yes |  |
| *17. In trials and cohort studies, do the analyses adjust for different lengths of follow‐up of patients, or in case‐control studies, is the time period between the intervention and outcome the same for cases and controls?*Where follow‐up was the same for all study patients the answer should yes. If different lengths of follow‐up were adjusted for by, for example, survival analysis the answer should be yes. Studies where differences in follow‐up are ignored should be answered no. | unable to determine |  |  |  |
| 1*8. Were the statistical tests used to assess the main outcomes appropriate?* The statistical techniques used must be appropriate to the data. For example nonparametric methods should be used for small sample sizes. Where little statistical analysis has been undertaken but where there is no evidence of bias, the question should be answered yes. If the distribution of the data (normal or not) is not described it must be assumed that the estimates used were appropriate and the question should be answered yes. |  |  | yes |  |
| *19. Was compliance with the intervention/s reliable*? Where there was non compliance with the allocated treatment or where there was contamination of one group, the question should be answered no. For studies where the effect of any misclassification was likely to bias any association to the null, the question should be answered yes |  |  | yes |  |
| *20. Were the main outcome measures used accurate (valid and reliable)?* For studies where the outcome measures are clearly described, the question should be answered yes. For studies which refer to other work or that demonstrates the outcome measures are accurate, the question should be answered as yes. |  |  | yes |  |
| **Internal validity ‐ confounding (selection bias)** |  |  |  |  |
| *21. Were the patients in different intervention groups (trials and cohort studies) or were the cases and controls (case‐control studies) recruited from the same population?* For example, patients for all comparison groups should be selected from the same hospital. The question should be answered unable to determine for cohort and casecontrol studies where there is no information concerning the source of patients included in the study. | unable to determine |  |  |  |
| *22. Were study subjects in different intervention groups (trials and cohort studies) or were the cases and controls (case‐control studies) recruited over the same period of time?* For a study which does not specify the time period over which patients were recruited, the question should be answered as unable to determine. | unable to determine |  |  |  |
| *23. Were study subjects randomised to intervention groups?* Studies which state that subjects were randomized should be answered yes except where method of randomisation would not ensure random allocation. For example alternate allocation would score no because it is predictable. |  | no |  |  |
| *24. Was the randomised intervention assignment concealed from both patients and health care staff until recruitment was complete and irrevocable?* All non‐randomised studies should be answered no. If assignment was oncealed from patients but not from staff, it should be answered no. | unable to determine |  |  |  |
| *25. Was there adequate adjustment for confounding in the analyses from which the main findings were drawn?* This question should be answered no for trials if: the main conclusions of the study were based on analyses of treatment rather than intention to treat; the distribution of known confounders in the different treatment groups was not described; or the distribution of known confounders differed between the treatment groups but was not taken into account in the analyses. In nonrandomized studies if the effect of the main confounders was not investigated or confounding was demonstrated but no adjustment was made in the final analyses the question should be answered as no. | unable to determine |  |  |  |
| *26. Were losses of patients to follow‐up taken into account?* If the numbers of patients lost to follow‐up are not reported, the question should be answered as unable to determine. If the proportion lost to follow‐ up was too small to affect the main findings, the question should be answered yes. | unable to determine |  |  |  |
| **Power** |  |  |  |  |
| *27. Did the study have sufficient power to detect a clinically important effect where the probability value for a difference being due to chance is less than 5%?*Sample sizes have been calculated to detect a difference of x% and y%. |  | no |  | no a priori power analysis was conducted, unable to determine effect size of interested outcome |
| **Total score:** |  |  | 11 |  |
|  |  |  |  |  |
| **Article Author & Name: Boudjeltia et al. 2008** | unable to determine | no | yes | **Notes/Justification** |
| **Reporting** | 0 | 0 | 1 |  |
| *1. Is the hypothesis/aim/objective of the study clearly described?* |  |  | yes |  |
| *2. Are the main outcomes to be measured clearly described in the Introduction or Methods section?* If the main outcomes are first mentioned in the Results section, the question should be answered no |  |  | yes |  |
| *3. Are the characteristics of the patients included in the study clearly described ?* In cohort studies and trials, inclusion and/or exclusion criteria should be given. In case‐control studies, a case‐definition and the source for controls should be given. |  |  | yes |  |
| *4. Are the interventions of interest clearly described?*Treatments and placebo (where relevant) that are to be compared should be clearly described. |  |  | yes |  |
| *5. Are the distributions of principal confounders in each group of subjects to be compared clearly described?* A list of principal confounders is provided. |  |  | yes (2) |  |
| *6. Are the main findings of the study clearly described?* Simple outcome data (including denominators and numerators) should be reported for all major findings so that the reader can check the major analyses and conclusions. (This question does not cover statistical tests which are considered below). |  |  | yes |  |
| *7. Does the study provide estimates of the random variability in the data for the main outcomes?* In non normally distributed data the inter‐quartile range of results should be reported. In normally distributed data the standard error, standard deviation or confidence intervals should be reported. If the distribution of the data is not described, it must be assumed that the estimates used were appropriate and the question should be answered yes. |  |  | yes |  |
| *8. Have all important adverse events that may be a consequence of the intervention been reported?* This should be answered yes if the study demonstrates that there was a comprehensive attempt to measure adverse events. (A list of possible adverse events is provided). |  | no |  |  |
| *9. Have the characteristics of patients lost to follow‐up been described?* This should be answered yes where there were no losses to follow‐up or where losses to follow‐up were so small that findings would be unaffected by their inclusion. This should be answered no where a study does not report the number of patients lost to follow‐up. | unable to determine |  |  |  |
| *10. Have actual probability values been reported ( e.g. 0.035 rather than <0.05) for the main outcomes except where the probability value is less than 0.001?* |  |  | yes |  |
| **External Validity** All the following criteria attempt to address the representativeness of the findings of the study and whether they may be generalised to the population from which the study subjects were derived. |  |  |  |  |
| *11. Were the subjects asked to participate in the study representative of the entire population from which they were recruited?* The study must identify the source population for patients and describe how the patients were selected. Patients would be representative if they comprised the entire source population, an unselected sample of consecutive patients, or a random sample. Random sampling is only feasible where a list of all members of the relevant population exists. Where a study does not report the proportion of the source population from which the patients are derived, the question should be answered as unable to determine. | unable to determine |  |  |  |
| *12. Were those subjects who were prepared to participate representative of the entire population from which they were recruited?*The proportion of those asked who agreed should be stated. Validation that the sample was representative would include demonstrating that the distribution of the main confounding factors was the same in the study sample and the source population. | unable to determine |  |  |  |
| *13. Were the staff, places, and facilities where the patients were treated, representative of the treatment the majority of patients receive?* For the question to be answered yes the study should demonstrate that the intervention was representative of that in use in the source population. The question should be answered no if, for example, the intervention was undertaken in a specialist centre unrepresentative of the hospitals most of the source population would attend. | unable to determine |  |  |  |
| **Internal validity – bias** |  |  |  |  |
| *14. Was an attempt made to blind study subjects to the intervention they have received?* For studies where the patients would have no way of knowing which  intervention they received, this should be answered yes. |  | no |  |  |
| *15. Was an attempt made to blind those measuring the main outcomes of the intervention?* |  | no |  |  |
| *16. If any of the results of the study were based on “data dredging”, was this made clear?* Any analyses that had not been planned at the outset of the study should be clearly indicated. If no retrospective unplanned subgroup analyses were reported, then answer yes. |  |  | yes |  |
| *17. In trials and cohort studies, do the analyses adjust for different lengths of follow‐up of patients, or in case‐control studies, is the time period between the intervention and outcome the same for cases and controls?*Where follow‐up was the same for all study patients the answer should yes. If different lengths of follow‐up were adjusted for by, for example, survival analysis the answer should be yes. Studies where differences in follow‐up are ignored should be answered no. | unable to determine |  |  |  |
| 1*8. Were the statistical tests used to assess the main outcomes appropriate?* The statistical techniques used must be appropriate to the data. For example nonparametric methods should be used for small sample sizes. Where little statistical analysis has been undertaken but where there is no evidence of bias, the question should be answered yes. If the distribution of the data (normal or not) is not described it must be assumed that the estimates used were appropriate and the question should be answered yes. |  |  | yes |  |
| *19. Was compliance with the intervention/s reliable*? Where there was non compliance with the allocated treatment or where there was contamination of one group, the question should be answered no. For studies where the effect of any misclassification was likely to bias any association to the null, the question should be answered yes |  |  | yes |  |
| *20. Were the main outcome measures used accurate (valid and reliable)?* For studies where the outcome measures are clearly described, the question should be answered yes. For studies which refer to other work or that demonstrates the outcome measures are accurate, the question should be answered as yes. |  |  | yes |  |
| **Internal validity ‐ confounding (selection bias)** |  |  |  |  |
| *21. Were the patients in different intervention groups (trials and cohort studies) or were the cases and controls (case‐control studies) recruited from the same population?* For example, patients for all comparison groups should be selected from the same hospital. The question should be answered unable to determine for cohort and casecontrol studies where there is no information concerning the source of patients included in the study. | unable to determine |  |  |  |
| *22. Were study subjects in different intervention groups (trials and cohort studies) or were the cases and controls (case‐control studies) recruited over the same period of time?* For a study which does not specify the time period over which patients were recruited, the question should be answered as unable to determine. | unable to determine |  |  |  |
| *23. Were study subjects randomised to intervention groups?* Studies which state that subjects were randomized should be answered yes except where method of randomisation would not ensure random allocation. For example alternate allocation would score no because it is predictable. |  | no |  |  |
| *24. Was the randomised intervention assignment concealed from both patients and health care staff until recruitment was complete and irrevocable?* All non‐randomised studies should be answered no. If assignment was oncealed from patients but not from staff, it should be answered no. |  | no |  |  |
| *25. Was there adequate adjustment for confounding in the analyses from which the main findings were drawn?* This question should be answered no for trials if: the main conclusions of the study were based on analyses of treatment rather than intention to treat; the distribution of known confounders in the different treatment groups was not described; or the distribution of known confounders differed between the treatment groups but was not taken into account in the analyses. In nonrandomized studies if the effect of the main confounders was not investigated or confounding was demonstrated but no adjustment was made in the final analyses the question should be answered as no. |  | no |  |  |
| *26. Were losses of patients to follow‐up taken into account?* If the numbers of patients lost to follow‐up are not reported, the question should be answered as unable to determine. If the proportion lost to follow‐ up was too small to affect the main findings, the question should be answered yes. | unable to determine |  |  |  |
| **Power** |  |  |  |  |
| *27. Did the study have sufficient power to detect a clinically important effect where the probability value for a difference being due to chance is less than 5%?*Sample sizes have been calculated to detect a difference of x% and y%. |  | no |  | no a priori power analysis was conducted, unable to determine effect size of interested outcome |
| **Total score:** |  |  | 13 |  |
|  |  |  |  |  |
| **Article Author & Name: Chennaoui et al. 2011** | unable to determine | no | yes | **Notes/Justification** |
| **Reporting** | 0 | 0 | 1 |  |
| *1. Is the hypothesis/aim/objective of the study clearly described?* |  |  | yes |  |
| *2. Are the main outcomes to be measured clearly described in the Introduction or Methods section?* If the main outcomes are first mentioned in the Results section, the question should be answered no |  |  | yes |  |
| *3. Are the characteristics of the patients included in the study clearly described ?* In cohort studies and trials, inclusion and/or exclusion criteria should be given. In case‐control studies, a case‐definition and the source for controls should be given. |  |  | yes |  |
| *4. Are the interventions of interest clearly described?*Treatments and placebo (where relevant) that are to be compared should be clearly described. |  |  | yes |  |
| *5. Are the distributions of principal confounders in each group of subjects to be compared clearly described?* A list of principal confounders is provided. |  |  | yes |  |
| *6. Are the main findings of the study clearly described?* Simple outcome data (including denominators and numerators) should be reported for all major findings so that the reader can check the major analyses and conclusions. (This question does not cover statistical tests which are considered below). |  |  | yes |  |
| *7. Does the study provide estimates of the random variability in the data for the main outcomes?* In non normally distributed data the inter‐quartile range of results should be reported. In normally distributed data the standard error, standard deviation or confidence intervals should be reported. If the distribution of the data is not described, it must be assumed that the estimates used were appropriate and the question should be answered yes. |  |  | yes |  |
| *8. Have all important adverse events that may be a consequence of the intervention been reported?* This should be answered yes if the study demonstrates that there was a comprehensive attempt to measure adverse events. (A list of possible adverse events is provided). |  | no |  |  |
| *9. Have the characteristics of patients lost to follow‐up been described?* This should be answered yes where there were no losses to follow‐up or where losses to follow‐up were so small that findings would be unaffected by their inclusion. This should be answered no where a study does not report the number of patients lost to follow‐up. | unable to determine |  |  |  |
| *10. Have actual probability values been reported ( e.g. 0.035 rather than <0.05) for the main outcomes except where the probability value is less than 0.001?* |  | no |  |  |
| **External Validity** All the following criteria attempt to address the representativeness of the findings of the study and whether they may be generalised to the population from which the study subjects were derived. |  |  |  |  |
| *11. Were the subjects asked to participate in the study representative of the entire population from which they were recruited?* The study must identify the source population for patients and describe how the patients were selected. Patients would be representative if they comprised the entire source population, an unselected sample of consecutive patients, or a random sample. Random sampling is only feasible where a list of all members of the relevant population exists. Where a study does not report the proportion of the source population from which the patients are derived, the question should be answered as unable to determine. | unable to determine |  |  |  |
| *12. Were those subjects who were prepared to participate representative of the entire population from which they were recruited?*The proportion of those asked who agreed should be stated. Validation that the sample was representative would include demonstrating that the distribution of the main confounding factors was the same in the study sample and the source population. | unable to determine |  |  |  |
| *13. Were the staff, places, and facilities where the patients were treated, representative of the treatment the majority of patients receive?* For the question to be answered yes the study should demonstrate that the intervention was representative of that in use in the source population. The question should be answered no if, for example, the intervention was undertaken in a specialist centre unrepresentative of the hospitals most of the source population would attend. |  | no |  |  |
| **Internal validity – bias** |  |  |  |  |
| *14. Was an attempt made to blind study subjects to the intervention they have received?* For studies where the patients would have no way of knowing which  intervention they received, this should be answered yes. |  | no |  |  |
| *15. Was an attempt made to blind those measuring the main outcomes of the intervention?* |  | no |  |  |
| *16. If any of the results of the study were based on “data dredging”, was this made clear?* Any analyses that had not been planned at the outset of the study should be clearly indicated. If no retrospective unplanned subgroup analyses were reported, then answer yes. |  |  |  |  |
| *17. In trials and cohort studies, do the analyses adjust for different lengths of follow‐up of patients, or in case‐control studies, is the time period between the intervention and outcome the same for cases and controls?*Where follow‐up was the same for all study patients the answer should yes. If different lengths of follow‐up were adjusted for by, for example, survival analysis the answer should be yes. Studies where differences in follow‐up are ignored should be answered no. | unable to determine |  |  |  |
| 1*8. Were the statistical tests used to assess the main outcomes appropriate?* The statistical techniques used must be appropriate to the data. For example nonparametric methods should be used for small sample sizes. Where little statistical analysis has been undertaken but where there is no evidence of bias, the question should be answered yes. If the distribution of the data (normal or not) is not described it must be assumed that the estimates used were appropriate and the question should be answered yes. |  |  | yes |  |
| *19. Was compliance with the intervention/s reliable*? Where there was non compliance with the allocated treatment or where there was contamination of one group, the question should be answered no. For studies where the effect of any misclassification was likely to bias any association to the null, the question should be answered yes |  |  | yes |  |
| *20. Were the main outcome measures used accurate (valid and reliable)?* For studies where the outcome measures are clearly described, the question should be answered yes. For studies which refer to other work or that demonstrates the outcome measures are accurate, the question should be answered as yes. |  |  | yes |  |
| **Internal validity ‐ confounding (selection bias)** |  |  |  |  |
| *21. Were the patients in different intervention groups (trials and cohort studies) or were the cases and controls (case‐control studies) recruited from the same population?* For example, patients for all comparison groups should be selected from the same hospital. The question should be answered unable to determine for cohort and casecontrol studies where there is no information concerning the source of patients included in the study. | unable to determine |  |  |  |
| *22. Were study subjects in different intervention groups (trials and cohort studies) or were the cases and controls (case‐control studies) recruited over the same period of time?* For a study which does not specify the time period over which patients were recruited, the question should be answered as unable to determine. | unable to determine |  |  |  |
| *23. Were study subjects randomised to intervention groups?* Studies which state that subjects were randomized should be answered yes except where method of randomisation would not ensure random allocation. For example alternate allocation would score no because it is predictable. |  | no |  |  |
| *24. Was the randomised intervention assignment concealed from both patients and health care staff until recruitment was complete and irrevocable?* All non‐randomised studies should be answered no. If assignment was oncealed from patients but not from staff, it should be answered no. |  | no |  |  |
| *25. Was there adequate adjustment for confounding in the analyses from which the main findings were drawn?* This question should be answered no for trials if: the main conclusions of the study were based on analyses of treatment rather than intention to treat; the distribution of known confounders in the different treatment groups was not described; or the distribution of known confounders differed between the treatment groups but was not taken into account in the analyses. In nonrandomized studies if the effect of the main confounders was not investigated or confounding was demonstrated but no adjustment was made in the final analyses the question should be answered as no. |  | no |  |  |
| *26. Were losses of patients to follow‐up taken into account?* If the numbers of patients lost to follow‐up are not reported, the question should be answered as unable to determine. If the proportion lost to follow‐ up was too small to affect the main findings, the question should be answered yes. | unable to determine |  |  |  |
| **Power** |  |  |  |  |
| *27. Did the study have sufficient power to detect a clinically important effect where the probability value for a difference being due to chance is less than 5%?*Sample sizes have been calculated to detect a difference of x% and y%. |  | no |  | no a priori power analysis was conducted, unable to determine effect size of interested outcome |
| **Total score:** |  |  | 10 |  |
|  |  |  |  |  |
| **Article Author & Name: Cullen et al. 2020** | unable to determine | no | yes | **Notes/Justification** |
| **Reporting** | 0 | 0 | 1 |  |
| *1. Is the hypothesis/aim/objective of the study clearly described?* |  |  | yes |  |
| *2. Are the main outcomes to be measured clearly described in the Introduction or Methods section?* If the main outcomes are first mentioned in the Results section, the question should be answered no |  |  | yes |  |
| *3. Are the characteristics of the patients included in the study clearly described ?* In cohort studies and trials, inclusion and/or exclusion criteria should be given. In case‐control studies, a case‐definition and the source for controls should be given. |  |  | yes |  |
| *4. Are the interventions of interest clearly described?*Treatments and placebo (where relevant) that are to be compared should be clearly described. |  |  | yes |  |
| *5. Are the distributions of principal confounders in each group of subjects to be compared clearly described?* A list of principal confounders is provided. |  |  | yes (2) |  |
| *6. Are the main findings of the study clearly described?* Simple outcome data (including denominators and numerators) should be reported for all major findings so that the reader can check the major analyses and conclusions. (This question does not cover statistical tests which are considered below). |  |  | yes |  |
| *7. Does the study provide estimates of the random variability in the data for the main outcomes?* In non normally distributed data the inter‐quartile range of results should be reported. In normally distributed data the standard error, standard deviation or confidence intervals should be reported. If the distribution of the data is not described, it must be assumed that the estimates used were appropriate and the question should be answered yes. |  |  | yes |  |
| *8. Have all important adverse events that may be a consequence of the intervention been reported?* This should be answered yes if the study demonstrates that there was a comprehensive attempt to measure adverse events. (A list of possible adverse events is provided). |  | no |  |  |
| *9. Have the characteristics of patients lost to follow‐up been described?* This should be answered yes where there were no losses to follow‐up or where losses to follow‐up were so small that findings would be unaffected by their inclusion. This should be answered no where a study does not report the number of patients lost to follow‐up. |  | no |  |  |
| *10. Have actual probability values been reported ( e.g. 0.035 rather than <0.05) for the main outcomes except where the probability value is less than 0.001?* |  |  | yes |  |
| **External Validity** All the following criteria attempt to address the representativeness of the findings of the study and whether they may be generalised to the population from which the study subjects were derived. |  |  |  |  |
| *11. Were the subjects asked to participate in the study representative of the entire population from which they were recruited?* The study must identify the source population for patients and describe how the patients were selected. Patients would be representative if they comprised the entire source population, an unselected sample of consecutive patients, or a random sample. Random sampling is only feasible where a list of all members of the relevant population exists. Where a study does not report the proportion of the source population from which the patients are derived, the question should be answered as unable to determine. | unable to determine |  |  |  |
| *12. Were those subjects who were prepared to participate representative of the entire population from which they were recruited?*The proportion of those asked who agreed should be stated. Validation that the sample was representative would include demonstrating that the distribution of the main confounding factors was the same in the study sample and the source population. | unable to determine |  |  |  |
| *13. Were the staff, places, and facilities where the patients were treated, representative of the treatment the majority of patients receive?* For the question to be answered yes the study should demonstrate that the intervention was representative of that in use in the source population. The question should be answered no if, for example, the intervention was undertaken in a specialist centre unrepresentative of the hospitals most of the source population would attend. | unable to determine |  |  |  |
| **Internal validity – bias** |  |  |  |  |
| *14. Was an attempt made to blind study subjects to the intervention they have received?* For studies where the patients would have no way of knowing which  intervention they received, this should be answered yes. |  | no |  |  |
| *15. Was an attempt made to blind those measuring the main outcomes of the intervention?* |  | no |  |  |
| *16. If any of the results of the study were based on “data dredging”, was this made clear?* Any analyses that had not been planned at the outset of the study should be clearly indicated. If no retrospective unplanned subgroup analyses were reported, then answer yes. |  |  | yes |  |
| *17. In trials and cohort studies, do the analyses adjust for different lengths of follow‐up of patients, or in case‐control studies, is the time period between the intervention and outcome the same for cases and controls?*Where follow‐up was the same for all study patients the answer should yes. If different lengths of follow‐up were adjusted for by, for example, survival analysis the answer should be yes. Studies where differences in follow‐up are ignored should be answered no. |  |  | yes |  |
| 1*8. Were the statistical tests used to assess the main outcomes appropriate?* The statistical techniques used must be appropriate to the data. For example nonparametric methods should be used for small sample sizes. Where little statistical analysis has been undertaken but where there is no evidence of bias, the question should be answered yes. If the distribution of the data (normal or not) is not described it must be assumed that the estimates used were appropriate and the question should be answered yes. |  |  | yes |  |
| *19. Was compliance with the intervention/s reliable*? Where there was non compliance with the allocated treatment or where there was contamination of one group, the question should be answered no. For studies where the effect of any misclassification was likely to bias any association to the null, the question should be answered yes |  |  | yes |  |
| *20. Were the main outcome measures used accurate (valid and reliable)?* For studies where the outcome measures are clearly described, the question should be answered yes. For studies which refer to other work or that demonstrates the outcome measures are accurate, the question should be answered as yes. |  |  | yes |  |
| **Internal validity ‐ confounding (selection bias)** |  |  |  |  |
| *21. Were the patients in different intervention groups (trials and cohort studies) or were the cases and controls (case‐control studies) recruited from the same population?* For example, patients for all comparison groups should be selected from the same hospital. The question should be answered unable to determine for cohort and casecontrol studies where there is no information concerning the source of patients included in the study. | unable to determine |  |  |  |
| *22. Were study subjects in different intervention groups (trials and cohort studies) or were the cases and controls (case‐control studies) recruited over the same period of time?* For a study which does not specify the time period over which patients were recruited, the question should be answered as unable to determine. | unable to determine |  |  |  |
| *23. Were study subjects randomised to intervention groups?* Studies which state that subjects were randomized should be answered yes except where method of randomisation would not ensure random allocation. For example alternate allocation would score no because it is predictable. |  |  | yes |  |
| *24. Was the randomised intervention assignment concealed from both patients and health care staff until recruitment was complete and irrevocable?* All non‐randomised studies should be answered no. If assignment was oncealed from patients but not from staff, it should be answered no. |  | no |  |  |
| *25. Was there adequate adjustment for confounding in the analyses from which the main findings were drawn?* This question should be answered no for trials if: the main conclusions of the study were based on analyses of treatment rather than intention to treat; the distribution of known confounders in the different treatment groups was not described; or the distribution of known confounders differed between the treatment groups but was not taken into account in the analyses. In nonrandomized studies if the effect of the main confounders was not investigated or confounding was demonstrated but no adjustment was made in the final analyses the question should be answered as no. |  | no |  |  |
| *26. Were losses of patients to follow‐up taken into account?* If the numbers of patients lost to follow‐up are not reported, the question should be answered as unable to determine. If the proportion lost to follow‐ up was too small to affect the main findings, the question should be answered yes. | unable to determine |  |  |  |
| **Power** |  |  |  |  |
| *27. Did the study have sufficient power to detect a clinically important effect where the probability value for a difference being due to chance is less than 5%?*Sample sizes have been calculated to detect a difference of x% and y%. |  | no |  | no a priori power analysis was conducted, small effect size |
| **Total score:** |  |  | 15 |  |
|  |  |  |  |  |
| **Article Author & Name: Dàttilo et al. 2020** | unable to determine | no | yes | **Notes/Justification** |
| **Reporting** | 0 | 0 | 1 |  |
| *1. Is the hypothesis/aim/objective of the study clearly described?* |  |  | yes |  |
| *2. Are the main outcomes to be measured clearly described in the Introduction or Methods section?* If the main outcomes are first mentioned in the Results section, the question should be answered no |  |  | yes |  |
| *3. Are the characteristics of the patients included in the study clearly described ?* In cohort studies and trials, inclusion and/or exclusion criteria should be given. In case‐control studies, a case‐definition and the source for controls should be given. |  |  | yes |  |
| *4. Are the interventions of interest clearly described?*Treatments and placebo (where relevant) that are to be compared should be clearly described. |  |  | yes |  |
| *5. Are the distributions of principal confounders in each group of subjects to be compared clearly described?* A list of principal confounders is provided. |  |  | yes (2) |  |
| *6. Are the main findings of the study clearly described?* Simple outcome data (including denominators and numerators) should be reported for all major findings so that the reader can check the major analyses and conclusions. (This question does not cover statistical tests which are considered below). |  |  | yes |  |
| *7. Does the study provide estimates of the random variability in the data for the main outcomes?* In non normally distributed data the inter‐quartile range of results should be reported. In normally distributed data the standard error, standard deviation or confidence intervals should be reported. If the distribution of the data is not described, it must be assumed that the estimates used were appropriate and the question should be answered yes. |  |  | yes |  |
| *8. Have all important adverse events that may be a consequence of the intervention been reported?* This should be answered yes if the study demonstrates that there was a comprehensive attempt to measure adverse events. (A list of possible adverse events is provided). |  | no |  |  |
| *9. Have the characteristics of patients lost to follow‐up been described?* This should be answered yes where there were no losses to follow‐up or where losses to follow‐up were so small that findings would be unaffected by their inclusion. This should be answered no where a study does not report the number of patients lost to follow‐up. | unable to determine |  |  |  |
| *10. Have actual probability values been reported ( e.g. 0.035 rather than <0.05) for the main outcomes except where the probability value is less than 0.001?* |  |  | yes |  |
| **External Validity** All the following criteria attempt to address the representativeness of the findings of the study and whether they may be generalised to the population from which the study subjects were derived. |  |  |  |  |
| *11. Were the subjects asked to participate in the study representative of the entire population from which they were recruited?* The study must identify the source population for patients and describe how the patients were selected. Patients would be representative if they comprised the entire source population, an unselected sample of consecutive patients, or a random sample. Random sampling is only feasible where a list of all members of the relevant population exists. Where a study does not report the proportion of the source population from which the patients are derived, the question should be answered as unable to determine. | unable to determine |  |  |  |
| *12. Were those subjects who were prepared to participate representative of the entire population from which they were recruited?*The proportion of those asked who agreed should be stated. Validation that the sample was representative would include demonstrating that the distribution of the main confounding factors was the same in the study sample and the source population. | unable to determine |  |  |  |
| *13. Were the staff, places, and facilities where the patients were treated, representative of the treatment the majority of patients receive?* For the question to be answered yes the study should demonstrate that the intervention was representative of that in use in the source population. The question should be answered no if, for example, the intervention was undertaken in a specialist centre unrepresentative of the hospitals most of the source population would attend. |  |  | yes |  |
| **Internal validity – bias** |  |  |  |  |
| *14. Was an attempt made to blind study subjects to the intervention they have received?* For studies where the patients would have no way of knowing which  intervention they received, this should be answered yes. |  | no |  |  |
| *15. Was an attempt made to blind those measuring the main outcomes of the intervention?* |  | no |  |  |
| *16. If any of the results of the study were based on “data dredging”, was this made clear?* Any analyses that had not been planned at the outset of the study should be clearly indicated. If no retrospective unplanned subgroup analyses were reported, then answer yes. |  |  | yes |  |
| *17. In trials and cohort studies, do the analyses adjust for different lengths of follow‐up of patients, or in case‐control studies, is the time period between the intervention and outcome the same for cases and controls?*Where follow‐up was the same for all study patients the answer should yes. If different lengths of follow‐up were adjusted for by, for example, survival analysis the answer should be yes. Studies where differences in follow‐up are ignored should be answered no. |  | no |  |  |
| 1*8. Were the statistical tests used to assess the main outcomes appropriate?* The statistical techniques used must be appropriate to the data. For example nonparametric methods should be used for small sample sizes. Where little statistical analysis has been undertaken but where there is no evidence of bias, the question should be answered yes. If the distribution of the data (normal or not) is not described it must be assumed that the estimates used were appropriate and the question should be answered yes. |  |  | yes |  |
| *19. Was compliance with the intervention/s reliable*? Where there was non compliance with the allocated treatment or where there was contamination of one group, the question should be answered no. For studies where the effect of any misclassification was likely to bias any association to the null, the question should be answered yes |  |  | yes |  |
| *20. Were the main outcome measures used accurate (valid and reliable)?* For studies where the outcome measures are clearly described, the question should be answered yes. For studies which refer to other work or that demonstrates the outcome measures are accurate, the question should be answered as yes. |  |  | yes |  |
| **Internal validity ‐ confounding (selection bias)** |  |  |  |  |
| *21. Were the patients in different intervention groups (trials and cohort studies) or were the cases and controls (case‐control studies) recruited from the same population?* For example, patients for all comparison groups should be selected from the same hospital. The question should be answered unable to determine for cohort and casecontrol studies where there is no information concerning the source of patients included in the study. | unable to determine |  |  |  |
| *22. Were study subjects in different intervention groups (trials and cohort studies) or were the cases and controls (case‐control studies) recruited over the same period of time?* For a study which does not specify the time period over which patients were recruited, the question should be answered as unable to determine. | unable to determine |  |  |  |
| *23. Were study subjects randomised to intervention groups?* Studies which state that subjects were randomized should be answered yes except where method of randomisation would not ensure random allocation. For example alternate allocation would score no because it is predictable. |  |  | yes |  |
| *24. Was the randomised intervention assignment concealed from both patients and health care staff until recruitment was complete and irrevocable?* All non‐randomised studies should be answered no. If assignment was oncealed from patients but not from staff, it should be answered no. |  | no |  |  |
| *25. Was there adequate adjustment for confounding in the analyses from which the main findings were drawn?* This question should be answered no for trials if: the main conclusions of the study were based on analyses of treatment rather than intention to treat; the distribution of known confounders in the different treatment groups was not described; or the distribution of known confounders differed between the treatment groups but was not taken into account in the analyses. In nonrandomized studies if the effect of the main confounders was not investigated or confounding was demonstrated but no adjustment was made in the final analyses the question should be answered as no. |  | no |  |  |
| *26. Were losses of patients to follow‐up taken into account?* If the numbers of patients lost to follow‐up are not reported, the question should be answered as unable to determine. If the proportion lost to follow‐ up was too small to affect the main findings, the question should be answered yes. | unable to determine |  |  |  |
| **Power** |  |  |  |  |
| *27. Did the study have sufficient power to detect a clinically important effect where the probability value for a difference being due to chance is less than 5%?*Sample sizes have been calculated to detect a difference of x% and y%. |  | no |  | no a priori power analysis was conducted, small effect size |
| **Total score:** |  |  | 15 |  |
|  |  |  |  |  |
| **Article Author & Name: Faraut et al. 2011** | unable to determine | no | yes | **Notes/Justification** |
| **Reporting** | 0 |  | 0 | 1 |
| *1. Is the hypothesis/aim/objective of the study clearly described?* |  |  | yes |  |
| *2. Are the main outcomes to be measured clearly described in the Introduction or Methods section?* If the main outcomes are first mentioned in the Results section, the question should be answered no |  |  | yes |  |
| *3. Are the characteristics of the patients included in the study clearly described ?* In cohort studies and trials, inclusion and/or exclusion criteria should be given. In case‐control studies, a case‐definition and the source for controls should be given. |  |  | yes |  |
| *4. Are the interventions of interest clearly described?*Treatments and placebo (where relevant) that are to be compared should be clearly described. |  |  | yes |  |
| *5. Are the distributions of principal confounders in each group of subjects to be compared clearly described?* A list of principal confounders is provided. |  | no |  |  |
| *6. Are the main findings of the study clearly described?* Simple outcome data (including denominators and numerators) should be reported for all major findings so that the reader can check the major analyses and conclusions. (This question does not cover statistical tests which are considered below). |  |  | yes |  |
| *7. Does the study provide estimates of the random variability in the data for the main outcomes?* In non normally distributed data the inter‐quartile range of results should be reported. In normally distributed data the standard error, standard deviation or confidence intervals should be reported. If the distribution of the data is not described, it must be assumed that the estimates used were appropriate and the question should be answered yes. |  |  | yes |  |
| *8. Have all important adverse events that may be a consequence of the intervention been reported?* This should be answered yes if the study demonstrates that there was a comprehensive attempt to measure adverse events. (A list of possible adverse events is provided). |  | no |  |  |
| *9. Have the characteristics of patients lost to follow‐up been described?* This should be answered yes where there were no losses to follow‐up or where losses to follow‐up were so small that findings would be unaffected by their inclusion. This should be answered no where a study does not report the number of patients lost to follow‐up. | unable to determine |  |  |  |
| *10. Have actual probability values been reported ( e.g. 0.035 rather than <0.05) for the main outcomes except where the probability value is less than 0.001?* |  |  | yes |  |
| **External Validity** All the following criteria attempt to address the representativeness of the findings of the study and whether they may be generalised to the population from which the study subjects were derived. |  |  |  |  |
| *11. Were the subjects asked to participate in the study representative of the entire population from which they were recruited?* The study must identify the source population for patients and describe how the patients were selected. Patients would be representative if they comprised the entire source population, an unselected sample of consecutive patients, or a random sample. Random sampling is only feasible where a list of all members of the relevant population exists. Where a study does not report the proportion of the source population from which the patients are derived, the question should be answered as unable to determine. | unable to determine |  |  |  |
| *12. Were those subjects who were prepared to participate representative of the entire population from which they were recruited?*The proportion of those asked who agreed should be stated. Validation that the sample was representative would include demonstrating that the distribution of the main confounding factors was the same in the study sample and the source population. | unable to determine |  |  |  |
| *13. Were the staff, places, and facilities where the patients were treated, representative of the treatment the majority of patients receive?* For the question to be answered yes the study should demonstrate that the intervention was representative of that in use in the source population. The question should be answered no if, for example, the intervention was undertaken in a specialist centre unrepresentative of the hospitals most of the source population would attend. | unable to determine |  |  |  |
| **Internal validity – bias** |  |  |  |  |
| *14. Was an attempt made to blind study subjects to the intervention they have received?* For studies where the patients would have no way of knowing which  intervention they received, this should be answered yes. |  | no |  |  |
| *15. Was an attempt made to blind those measuring the main outcomes of the intervention?* |  | no |  |  |
| *16. If any of the results of the study were based on “data dredging”, was this made clear?* Any analyses that had not been planned at the outset of the study should be clearly indicated. If no retrospective unplanned subgroup analyses were reported, then answer yes. |  |  | yes |  |
| *17. In trials and cohort studies, do the analyses adjust for different lengths of follow‐up of patients, or in case‐control studies, is the time period between the intervention and outcome the same for cases and controls?*Where follow‐up was the same for all study patients the answer should yes. If different lengths of follow‐up were adjusted for by, for example, survival analysis the answer should be yes. Studies where differences in follow‐up are ignored should be answered no. | unable to determine |  |  |  |
| 1*8. Were the statistical tests used to assess the main outcomes appropriate?* The statistical techniques used must be appropriate to the data. For example nonparametric methods should be used for small sample sizes. Where little statistical analysis has been undertaken but where there is no evidence of bias, the question should be answered yes. If the distribution of the data (normal or not) is not described it must be assumed that the estimates used were appropriate and the question should be answered yes. |  |  | yes |  |
| *19. Was compliance with the intervention/s reliable*? Where there was non compliance with the allocated treatment or where there was contamination of one group, the question should be answered no. For studies where the effect of any misclassification was likely to bias any association to the null, the question should be answered yes |  |  | yes |  |
| *20. Were the main outcome measures used accurate (valid and reliable)?* For studies where the outcome measures are clearly described, the question should be answered yes. For studies which refer to other work or that demonstrates the outcome measures are accurate, the question should be answered as yes. |  |  | yes |  |
| **Internal validity ‐ confounding (selection bias)** |  |  |  |  |
| *21. Were the patients in different intervention groups (trials and cohort studies) or were the cases and controls (case‐control studies) recruited from the same population?* For example, patients for all comparison groups should be selected from the same hospital. The question should be answered unable to determine for cohort and casecontrol studies where there is no information concerning the source of patients included in the study. | unable to determine |  |  |  |
| *22. Were study subjects in different intervention groups (trials and cohort studies) or were the cases and controls (case‐control studies) recruited over the same period of time?* For a study which does not specify the time period over which patients were recruited, the question should be answered as unable to determine. | unable to determine |  |  |  |
| *23. Were study subjects randomised to intervention groups?* Studies which state that subjects were randomized should be answered yes except where method of randomisation would not ensure random allocation. For example alternate allocation would score no because it is predictable. |  | no |  |  |
| *24. Was the randomised intervention assignment concealed from both patients and health care staff until recruitment was complete and irrevocable?* All non‐randomised studies should be answered no. If assignment was oncealed from patients but not from staff, it should be answered no. |  | no |  |  |
| *25. Was there adequate adjustment for confounding in the analyses from which the main findings were drawn?* This question should be answered no for trials if: the main conclusions of the study were based on analyses of treatment rather than intention to treat; the distribution of known confounders in the different treatment groups was not described; or the distribution of known confounders differed between the treatment groups but was not taken into account in the analyses. In nonrandomized studies if the effect of the main confounders was not investigated or confounding was demonstrated but no adjustment was made in the final analyses the question should be answered as no. |  | no |  |  |
| *26. Were losses of patients to follow‐up taken into account?* If the numbers of patients lost to follow‐up are not reported, the question should be answered as unable to determine. If the proportion lost to follow‐ up was too small to affect the main findings, the question should be answered yes. | unable to determine |  |  |  |
| **Power** |  |  |  |  |
| *27. Did the study have sufficient power to detect a clinically important effect where the probability value for a difference being due to chance is less than 5%?*Sample sizes have been calculated to detect a difference of x% and y%. |  | no |  | no a priori power analysis was conducted, unable to determine effect size of interested outcome |
| **Total score:** |  |  | 11 |  |
|  |  |  |  |  |
| **Article Author & Name: Frey et al. 2007** | unable to determine | no | yes | **Notes/Justification** |
| **Reporting** | 0 | 0 | 1 |  |
| *1. Is the hypothesis/aim/objective of the study clearly described?* |  |  | yes |  |
| *2. Are the main outcomes to be measured clearly described in the Introduction or Methods section?* If the main outcomes are first mentioned in the Results section, the question should be answered no |  |  | yes |  |
| *3. Are the characteristics of the patients included in the study clearly described ?* In cohort studies and trials, inclusion and/or exclusion criteria should be given. In case‐control studies, a case‐definition and the source for controls should be given. |  |  | yes |  |
| *4. Are the interventions of interest clearly described?*Treatments and placebo (where relevant) that are to be compared should be clearly described. |  |  | yes |  |
| *5. Are the distributions of principal confounders in each group of subjects to be compared clearly described?* A list of principal confounders is provided. |  |  | yes (2) |  |
| *6. Are the main findings of the study clearly described?* Simple outcome data (including denominators and numerators) should be reported for all major findings so that the reader can check the major analyses and conclusions. (This question does not cover statistical tests which are considered below). |  |  | yes |  |
| *7. Does the study provide estimates of the random variability in the data for the main outcomes?* In non normally distributed data the inter‐quartile range of results should be reported. In normally distributed data the standard error, standard deviation or confidence intervals should be reported. If the distribution of the data is not described, it must be assumed that the estimates used were appropriate and the question should be answered yes. |  |  | yes |  |
| *8. Have all important adverse events that may be a consequence of the intervention been reported?* This should be answered yes if the study demonstrates that there was a comprehensive attempt to measure adverse events. (A list of possible adverse events is provided). |  | no |  |  |
| *9. Have the characteristics of patients lost to follow‐up been described?* This should be answered yes where there were no losses to follow‐up or where losses to follow‐up were so small that findings would be unaffected by their inclusion. This should be answered no where a study does not report the number of patients lost to follow‐up. |  | no |  |  |
| *10. Have actual probability values been reported ( e.g. 0.035 rather than <0.05) for the main outcomes except where the probability value is less than 0.001?* |  | no |  |  |
| **External Validity** All the following criteria attempt to address the representativeness of the findings of the study and whether they may be generalised to the population from which the study subjects were derived. |  |  |  |  |
| *11. Were the subjects asked to participate in the study representative of the entire population from which they were recruited?* The study must identify the source population for patients and describe how the patients were selected. Patients would be representative if they comprised the entire source population, an unselected sample of consecutive patients, or a random sample. Random sampling is only feasible where a list of all members of the relevant population exists. Where a study does not report the proportion of the source population from which the patients are derived, the question should be answered as unable to determine. | unable to determine |  |  |  |
| *12. Were those subjects who were prepared to participate representative of the entire population from which they were recruited?*The proportion of those asked who agreed should be stated. Validation that the sample was representative would include demonstrating that the distribution of the main confounding factors was the same in the study sample and the source population. | unable to determine |  |  |  |
| *13. Were the staff, places, and facilities where the patients were treated, representative of the treatment the majority of patients receive?* For the question to be answered yes the study should demonstrate that the intervention was representative of that in use in the source population. The question should be answered no if, for example, the intervention was undertaken in a specialist centre unrepresentative of the hospitals most of the source population would attend. |  | no |  |  |
| **Internal validity – bias** |  |  |  |  |
| *14. Was an attempt made to blind study subjects to the intervention they have received?* For studies where the patients would have no way of knowing which  intervention they received, this should be answered yes. |  | no |  |  |
| *15. Was an attempt made to blind those measuring the main outcomes of the intervention?* |  | no |  |  |
| *16. If any of the results of the study were based on “data dredging”, was this made clear?* Any analyses that had not been planned at the outset of the study should be clearly indicated. If no retrospective unplanned subgroup analyses were reported, then answer yes. |  |  | yes |  |
| *17. In trials and cohort studies, do the analyses adjust for different lengths of follow‐up of patients, or in case‐control studies, is the time period between the intervention and outcome the same for cases and controls?*Where follow‐up was the same for all study patients the answer should yes. If different lengths of follow‐up were adjusted for by, for example, survival analysis the answer should be yes. Studies where differences in follow‐up are ignored should be answered no. |  |  | yes |  |
| 1*8. Were the statistical tests used to assess the main outcomes appropriate?* The statistical techniques used must be appropriate to the data. For example nonparametric methods should be used for small sample sizes. Where little statistical analysis has been undertaken but where there is no evidence of bias, the question should be answered yes. If the distribution of the data (normal or not) is not described it must be assumed that the estimates used were appropriate and the question should be answered yes. |  |  | yes |  |
| *19. Was compliance with the intervention/s reliable*? Where there was non compliance with the allocated treatment or where there was contamination of one group, the question should be answered no. For studies where the effect of any misclassification was likely to bias any association to the null, the question should be answered yes |  |  | yes |  |
| *20. Were the main outcome measures used accurate (valid and reliable)?* For studies where the outcome measures are clearly described, the question should be answered yes. For studies which refer to other work or that demonstrates the outcome measures are accurate, the question should be answered as yes. |  |  | yes |  |
| **Internal validity ‐ confounding (selection bias)** |  |  |  |  |
| *21. Were the patients in different intervention groups (trials and cohort studies) or were the cases and controls (case‐control studies) recruited from the same population?* For example, patients for all comparison groups should be selected from the same hospital. The question should be answered unable to determine for cohort and casecontrol studies where there is no information concerning the source of patients included in the study. | unable to determine |  |  |  |
| *22. Were study subjects in different intervention groups (trials and cohort studies) or were the cases and controls (case‐control studies) recruited over the same period of time?* For a study which does not specify the time period over which patients were recruited, the question should be answered as unable to determine. | unable to determine |  |  |  |
| *23. Were study subjects randomised to intervention groups?* Studies which state that subjects were randomized should be answered yes except where method of randomisation would not ensure random allocation. For example alternate allocation would score no because it is predictable. |  | no |  |  |
| *24. Was the randomised intervention assignment concealed from both patients and health care staff until recruitment was complete and irrevocable?* All non‐randomised studies should be answered no. If assignment was oncealed from patients but not from staff, it should be answered no. |  | no |  |  |
| *25. Was there adequate adjustment for confounding in the analyses from which the main findings were drawn?* This question should be answered no for trials if: the main conclusions of the study were based on analyses of treatment rather than intention to treat; the distribution of known confounders in the different treatment groups was not described; or the distribution of known confounders differed between the treatment groups but was not taken into account in the analyses. In nonrandomized studies if the effect of the main confounders was not investigated or confounding was demonstrated but no adjustment was made in the final analyses the question should be answered as no. |  | no |  |  |
| *26. Were losses of patients to follow‐up taken into account?* If the numbers of patients lost to follow‐up are not reported, the question should be answered as unable to determine. If the proportion lost to follow‐ up was too small to affect the main findings, the question should be answered yes. |  |  | yes |  |
| **Power** |  |  |  |  |
| *27. Did the study have sufficient power to detect a clinically important effect where the probability value for a difference being due to chance is less than 5%?*Sample sizes have been calculated to detect a difference of x% and y%. |  | no |  | no a priori power analysis was conducted, unable to determine effect size of interested outcome |
| **Total score:** |  |  | 14 |  |
|  |  |  |  |  |
| **Article Author & Name: Haack et al. 2007** | unable to determine | no | yes | **Notes/Justification** |
| **Reporting** |  |  |  |  |
| *1. Is the hypothesis/aim/objective of the study clearly described?* |  |  | yes |  |
| *2. Are the main outcomes to be measured clearly described in the Introduction or Methods section?* If the main outcomes are first mentioned in the Results section, the question should be answered no |  |  | yes |  |
| *3. Are the characteristics of the patients included in the study clearly described ?* In cohort studies and trials, inclusion and/or exclusion criteria should be given. In case‐control studies, a case‐definition and the source for controls should be given. |  |  | yes |  |
| *4. Are the interventions of interest clearly described?*Treatments and placebo (where relevant) that are to be compared should be clearly described. |  |  | yes |  |
| *5. Are the distributions of principal confounders in each group of subjects to be compared clearly described?* A list of principal confounders is provided. |  |  | yes (2) |  |
| *6. Are the main findings of the study clearly described?* Simple outcome data (including denominators and numerators) should be reported for all major findings so that the reader can check the major analyses and conclusions. (This question does not cover statistical tests which are considered below). |  |  | yes |  |
| *7. Does the study provide estimates of the random variability in the data for the main outcomes?* In non normally distributed data the inter‐quartile range of results should be reported. In normally distributed data the standard error, standard deviation or confidence intervals should be reported. If the distribution of the data is not described, it must be assumed that the estimates used were appropriate and the question should be answered yes. |  |  | yes |  |
| *8. Have all important adverse events that may be a consequence of the intervention been reported?* This should be answered yes if the study demonstrates that there was a comprehensive attempt to measure adverse events. (A list of possible adverse events is provided). |  | no |  |  |
| *9. Have the characteristics of patients lost to follow‐up been described?* This should be answered yes where there were no losses to follow‐up or where losses to follow‐up were so small that findings would be unaffected by their inclusion. This should be answered no where a study does not report the number of patients lost to follow‐up. | unable to determine |  |  |  |
| *10. Have actual probability values been reported ( e.g. 0.035 rather than <0.05) for the main outcomes except where the probability value is less than 0.001?* |  |  | yes |  |
| **External Validity** All the following criteria attempt to address the representativeness of the findings of the study and whether they may be generalised to the population from which the study subjects were derived. |  |  |  |  |
| *11. Were the subjects asked to participate in the study representative of the entire population from which they were recruited?* The study must identify the source population for patients and describe how the patients were selected. Patients would be representative if they comprised the entire source population, an unselected sample of consecutive patients, or a random sample. Random sampling is only feasible where a list of all members of the relevant population exists. Where a study does not report the proportion of the source population from which the patients are derived, the question should be answered as unable to determine. | unable to determine |  |  |  |
| *12. Were those subjects who were prepared to participate representative of the entire population from which they were recruited?*The proportion of those asked who agreed should be stated. Validation that the sample was representative would include demonstrating that the distribution of the main confounding factors was the same in the study sample and the source population. | unable to determine |  |  |  |
| *13. Were the staff, places, and facilities where the patients were treated, representative of the treatment the majority of patients receive?* For the question to be answered yes the study should demonstrate that the intervention was representative of that in use in the source population. The question should be answered no if, for example, the intervention was undertaken in a specialist centre unrepresentative of the hospitals most of the source population would attend. |  | no |  |  |
| **Internal validity – bias** |  |  |  |  |
| *14. Was an attempt made to blind study subjects to the intervention they have received?* For studies where the patients would have no way of knowing which  intervention they received, this should be answered yes. |  | no |  |  |
| *15. Was an attempt made to blind those measuring the main outcomes of the intervention?* |  | no |  |  |
| *16. If any of the results of the study were based on “data dredging”, was this made clear?* Any analyses that had not been planned at the outset of the study should be clearly indicated. If no retrospective unplanned subgroup analyses were reported, then answer yes. |  |  | yes |  |
| *17. In trials and cohort studies, do the analyses adjust for different lengths of follow‐up of patients, or in case‐control studies, is the time period between the intervention and outcome the same for cases and controls?*Where follow‐up was the same for all study patients the answer should yes. If different lengths of follow‐up were adjusted for by, for example, survival analysis the answer should be yes. Studies where differences in follow‐up are ignored should be answered no. |  | no |  |  |
| 1*8. Were the statistical tests used to assess the main outcomes appropriate?* The statistical techniques used must be appropriate to the data. For example nonparametric methods should be used for small sample sizes. Where little statistical analysis has been undertaken but where there is no evidence of bias, the question should be answered yes. If the distribution of the data (normal or not) is not described it must be assumed that the estimates used were appropriate and the question should be answered yes. |  |  | yes |  |
| *19. Was compliance with the intervention/s reliable*? Where there was non compliance with the allocated treatment or where there was contamination of one group, the question should be answered no. For studies where the effect of any misclassification was likely to bias any association to the null, the question should be answered yes |  |  | yes |  |
| *20. Were the main outcome measures used accurate (valid and reliable)?* For studies where the outcome measures are clearly described, the question should be answered yes. For studies which refer to other work or that demonstrates the outcome measures are accurate, the question should be answered as yes. |  |  | yes |  |
| **Internal validity ‐ confounding (selection bias)** |  |  |  |  |
| *21. Were the patients in different intervention groups (trials and cohort studies) or were the cases and controls (case‐control studies) recruited from the same population?* For example, patients for all comparison groups should be selected from the same hospital. The question should be answered unable to determine for cohort and casecontrol studies where there is no information concerning the source of patients included in the study. |  |  | yes |  |
| *22. Were study subjects in different intervention groups (trials and cohort studies) or were the cases and controls (case‐control studies) recruited over the same period of time?* For a study which does not specify the time period over which patients were recruited, the question should be answered as unable to determine. | unable to determine |  |  |  |
| *23. Were study subjects randomised to intervention groups?* Studies which state that subjects were randomized should be answered yes except where method of randomisation would not ensure random allocation. For example alternate allocation would score no because it is predictable. |  |  | yes |  |
| *24. Was the randomised intervention assignment concealed from both patients and health care staff until recruitment was complete and irrevocable?* All non‐randomised studies should be answered no. If assignment was oncealed from patients but not from staff, it should be answered no. |  | no |  |  |
| *25. Was there adequate adjustment for confounding in the analyses from which the main findings were drawn?* This question should be answered no for trials if: the main conclusions of the study were based on analyses of treatment rather than intention to treat; the distribution of known confounders in the different treatment groups was not described; or the distribution of known confounders differed between the treatment groups but was not taken into account in the analyses. In nonrandomized studies if the effect of the main confounders was not investigated or confounding was demonstrated but no adjustment was made in the final analyses the question should be answered as no. |  | no |  |  |
| *26. Were losses of patients to follow‐up taken into account?* If the numbers of patients lost to follow‐up are not reported, the question should be answered as unable to determine. If the proportion lost to follow‐ up was too small to affect the main findings, the question should be answered yes. | unable to determine |  |  |  |
| **Power** |  |  |  |  |
| *27. Did the study have sufficient power to detect a clinically important effect where the probability value for a difference being due to chance is less than 5%?*Sample sizes have been calculated to detect a difference of x% and y%. |  |  | yes | no a priori power analysis was conducted, adequate effect size |
| **Total score:** |  |  | 16 |  |
|  |  |  |  |  |
| **Article Author & Name: Heiser et al. 1997** | unable to determine | no | yes | **Notes/Justification** |
| **Reporting** | 0 | 0 | 1 |  |
| *1. Is the hypothesis/aim/objective of the study clearly described?* |  |  | yes |  |
| *2. Are the main outcomes to be measured clearly described in the Introduction or Methods section?* If the main outcomes are first mentioned in the Results section, the question should be answered no |  |  | yes |  |
| *3. Are the characteristics of the patients included in the study clearly described ?* In cohort studies and trials, inclusion and/or exclusion criteria should be given. In case‐control studies, a case‐definition and the source for controls should be given. |  |  | yes |  |
| *4. Are the interventions of interest clearly described?*Treatments and placebo (where relevant) that are to be compared should be clearly described. |  |  | yes |  |
| *5. Are the distributions of principal confounders in each group of subjects to be compared clearly described?* A list of principal confounders is provided. |  | no |  |  |
| *6. Are the main findings of the study clearly described?* Simple outcome data (including denominators and numerators) should be reported for all major findings so that the reader can check the major analyses and conclusions. (This question does not cover statistical tests which are considered below). |  |  | yes |  |
| *7. Does the study provide estimates of the random variability in the data for the main outcomes?* In non normally distributed data the inter‐quartile range of results should be reported. In normally distributed data the standard error, standard deviation or confidence intervals should be reported. If the distribution of the data is not described, it must be assumed that the estimates used were appropriate and the question should be answered yes. |  |  | yes |  |
| *8. Have all important adverse events that may be a consequence of the intervention been reported?* This should be answered yes if the study demonstrates that there was a comprehensive attempt to measure adverse events. (A list of possible adverse events is provided). |  | no |  |  |
| *9. Have the characteristics of patients lost to follow‐up been described?* This should be answered yes where there were no losses to follow‐up or where losses to follow‐up were so small that findings would be unaffected by their inclusion. This should be answered no where a study does not report the number of patients lost to follow‐up. | unable to determine |  |  |  |
| *10. Have actual probability values been reported ( e.g. 0.035 rather than <0.05) for the main outcomes except where the probability value is less than 0.001?* |  |  | yes |  |
| **External Validity** All the following criteria attempt to address the representativeness of the findings of the study and whether they may be generalised to the population from which the study subjects were derived. |  |  |  |  |
| *11. Were the subjects asked to participate in the study representative of the entire population from which they were recruited?* The study must identify the source population for patients and describe how the patients were selected. Patients would be representative if they comprised the entire source population, an unselected sample of consecutive patients, or a random sample. Random sampling is only feasible where a list of all members of the relevant population exists. Where a study does not report the proportion of the source population from which the patients are derived, the question should be answered as unable to determine. | unable to determine |  |  |  |
| *12. Were those subjects who were prepared to participate representative of the entire population from which they were recruited?*The proportion of those asked who agreed should be stated. Validation that the sample was representative would include demonstrating that the distribution of the main confounding factors was the same in the study sample and the source population. | unable to determine |  |  |  |
| *13. Were the staff, places, and facilities where the patients were treated, representative of the treatment the majority of patients receive?* For the question to be answered yes the study should demonstrate that the intervention was representative of that in use in the source population. The question should be answered no if, for example, the intervention was undertaken in a specialist centre unrepresentative of the hospitals most of the source population would attend. | unable to determine |  |  |  |
| **Internal validity – bias** |  |  |  |  |
| *14. Was an attempt made to blind study subjects to the intervention they have received?* For studies where the patients would have no way of knowing which  intervention they received, this should be answered yes. |  | no |  |  |
| *15. Was an attempt made to blind those measuring the main outcomes of the intervention?* |  | no |  |  |
| *16. If any of the results of the study were based on “data dredging”, was this made clear?* Any analyses that had not been planned at the outset of the study should be clearly indicated. If no retrospective unplanned subgroup analyses were reported, then answer yes. |  |  | yes |  |
| *17. In trials and cohort studies, do the analyses adjust for different lengths of follow‐up of patients, or in case‐control studies, is the time period between the intervention and outcome the same for cases and controls?*Where follow‐up was the same for all study patients the answer should yes. If different lengths of follow‐up were adjusted for by, for example, survival analysis the answer should be yes. Studies where differences in follow‐up are ignored should be answered no. | unable to determine |  |  |  |
| 1*8. Were the statistical tests used to assess the main outcomes appropriate?* The statistical techniques used must be appropriate to the data. For example nonparametric methods should be used for small sample sizes. Where little statistical analysis has been undertaken but where there is no evidence of bias, the question should be answered yes. If the distribution of the data (normal or not) is not described it must be assumed that the estimates used were appropriate and the question should be answered yes. |  |  | yes |  |
| *19. Was compliance with the intervention/s reliable*? Where there was non compliance with the allocated treatment or where there was contamination of one group, the question should be answered no. For studies where the effect of any misclassification was likely to bias any association to the null, the question should be answered yes |  |  | yes |  |
| *20. Were the main outcome measures used accurate (valid and reliable)?* For studies where the outcome measures are clearly described, the question should be answered yes. For studies which refer to other work or that demonstrates the outcome measures are accurate, the question should be answered as yes. |  |  | yes |  |
| **Internal validity ‐ confounding (selection bias)** |  |  |  |  |
| *21. Were the patients in different intervention groups (trials and cohort studies) or were the cases and controls (case‐control studies) recruited from the same population?* For example, patients for all comparison groups should be selected from the same hospital. The question should be answered unable to determine for cohort and casecontrol studies where there is no information concerning the source of patients included in the study. | unable to determine |  |  |  |
| *22. Were study subjects in different intervention groups (trials and cohort studies) or were the cases and controls (case‐control studies) recruited over the same period of time?* For a study which does not specify the time period over which patients were recruited, the question should be answered as unable to determine. | unable to determine |  |  |  |
| *23. Were study subjects randomised to intervention groups?* Studies which state that subjects were randomized should be answered yes except where method of randomisation would not ensure random allocation. For example alternate allocation would score no because it is predictable. |  | no |  |  |
| *24. Was the randomised intervention assignment concealed from both patients and health care staff until recruitment was complete and irrevocable?* All non‐randomised studies should be answered no. If assignment was oncealed from patients but not from staff, it should be answered no. |  | no |  |  |
| *25. Was there adequate adjustment for confounding in the analyses from which the main findings were drawn?* This question should be answered no for trials if: the main conclusions of the study were based on analyses of treatment rather than intention to treat; the distribution of known confounders in the different treatment groups was not described; or the distribution of known confounders differed between the treatment groups but was not taken into account in the analyses. In nonrandomized studies if the effect of the main confounders was not investigated or confounding was demonstrated but no adjustment was made in the final analyses the question should be answered as no. |  | no |  |  |
| *26. Were losses of patients to follow‐up taken into account?* If the numbers of patients lost to follow‐up are not reported, the question should be answered as unable to determine. If the proportion lost to follow‐ up was too small to affect the main findings, the question should be answered yes. | unable to determine |  |  |  |
| **Power** |  |  |  |  |
| *27. Did the study have sufficient power to detect a clinically important effect where the probability value for a difference being due to chance is less than 5%?*Sample sizes have been calculated to detect a difference of x% and y%. |  | no |  | no a priori power analysis was conducted, unable to determine effect size of interested outcome |
| **Total score:** |  |  | 11 |  |
|  |  |  |  |  |
| **Article Author & Name: Heiser et al. 2001** | unable to determine | no | yes | **Notes/Justification** |
| **Reporting** | 0 | 0 | 1 |  |
| *1. Is the hypothesis/aim/objective of the study clearly described?* |  |  | yes |  |
| *2. Are the main outcomes to be measured clearly described in the Introduction or Methods section?* If the main outcomes are first mentioned in the Results section, the question should be answered no |  |  | yes |  |
| *3. Are the characteristics of the patients included in the study clearly described ?* In cohort studies and trials, inclusion and/or exclusion criteria should be given. In case‐control studies, a case‐definition and the source for controls should be given. |  |  | yes |  |
| *4. Are the interventions of interest clearly described?*Treatments and placebo (where relevant) that are to be compared should be clearly described. |  |  | yes |  |
| *5. Are the distributions of principal confounders in each group of subjects to be compared clearly described?* A list of principal confounders is provided. |  |  | yes (2) |  |
| *6. Are the main findings of the study clearly described?* Simple outcome data (including denominators and numerators) should be reported for all major findings so that the reader can check the major analyses and conclusions. (This question does not cover statistical tests which are considered below). |  |  | yes |  |
| *7. Does the study provide estimates of the random variability in the data for the main outcomes?* In non normally distributed data the inter‐quartile range of results should be reported. In normally distributed data the standard error, standard deviation or confidence intervals should be reported. If the distribution of the data is not described, it must be assumed that the estimates used were appropriate and the question should be answered yes. |  |  | yes |  |
| *8. Have all important adverse events that may be a consequence of the intervention been reported?* This should be answered yes if the study demonstrates that there was a comprehensive attempt to measure adverse events. (A list of possible adverse events is provided). |  | no |  |  |
| *9. Have the characteristics of patients lost to follow‐up been described?* This should be answered yes where there were no losses to follow‐up or where losses to follow‐up were so small that findings would be unaffected by their inclusion. This should be answered no where a study does not report the number of patients lost to follow‐up. | unable to determine |  |  |  |
| *10. Have actual probability values been reported ( e.g. 0.035 rather than <0.05) for the main outcomes except where the probability value is less than 0.001?* |  |  | yes |  |
| **External Validity** All the following criteria attempt to address the representativeness of the findings of the study and whether they may be generalised to the population from which the study subjects were derived. |  |  |  |  |
| *11. Were the subjects asked to participate in the study representative of the entire population from which they were recruited?* The study must identify the source population for patients and describe how the patients were selected. Patients would be representative if they comprised the entire source population, an unselected sample of consecutive patients, or a random sample. Random sampling is only feasible where a list of all members of the relevant population exists. Where a study does not report the proportion of the source population from which the patients are derived, the question should be answered as unable to determine. | unable to determine |  |  |  |
| *12. Were those subjects who were prepared to participate representative of the entire population from which they were recruited?*The proportion of those asked who agreed should be stated. Validation that the sample was representative would include demonstrating that the distribution of the main confounding factors was the same in the study sample and the source population. | unable to determine |  |  |  |
| *13. Were the staff, places, and facilities where the patients were treated, representative of the treatment the majority of patients receive?* For the question to be answered yes the study should demonstrate that the intervention was representative of that in use in the source population. The question should be answered no if, for example, the intervention was undertaken in a specialist centre unrepresentative of the hospitals most of the source population would attend. | unable to determine |  |  |  |
| **Internal validity – bias** |  |  |  |  |
| *14. Was an attempt made to blind study subjects to the intervention they have received?* For studies where the patients would have no way of knowing which  intervention they received, this should be answered yes. |  | no |  |  |
| *15. Was an attempt made to blind those measuring the main outcomes of the intervention?* |  | no |  |  |
| *16. If any of the results of the study were based on “data dredging”, was this made clear?* Any analyses that had not been planned at the outset of the study should be clearly indicated. If no retrospective unplanned subgroup analyses were reported, then answer yes. |  |  | yes |  |
| *17. In trials and cohort studies, do the analyses adjust for different lengths of follow‐up of patients, or in case‐control studies, is the time period between the intervention and outcome the same for cases and controls?*Where follow‐up was the same for all study patients the answer should yes. If different lengths of follow‐up were adjusted for by, for example, survival analysis the answer should be yes. Studies where differences in follow‐up are ignored should be answered no. | unable to determine |  |  |  |
| 1*8. Were the statistical tests used to assess the main outcomes appropriate?* The statistical techniques used must be appropriate to the data. For example nonparametric methods should be used for small sample sizes. Where little statistical analysis has been undertaken but where there is no evidence of bias, the question should be answered yes. If the distribution of the data (normal or not) is not described it must be assumed that the estimates used were appropriate and the question should be answered yes. |  |  | yes |  |
| *19. Was compliance with the intervention/s reliable*? Where there was non compliance with the allocated treatment or where there was contamination of one group, the question should be answered no. For studies where the effect of any misclassification was likely to bias any association to the null, the question should be answered yes |  |  | yes |  |
| *20. Were the main outcome measures used accurate (valid and reliable)?* For studies where the outcome measures are clearly described, the question should be answered yes. For studies which refer to other work or that demonstrates the outcome measures are accurate, the question should be answered as yes. |  |  | yes |  |
| **Internal validity ‐ confounding (selection bias)** |  |  |  |  |
| *21. Were the patients in different intervention groups (trials and cohort studies) or were the cases and controls (case‐control studies) recruited from the same population?* For example, patients for all comparison groups should be selected from the same hospital. The question should be answered unable to determine for cohort and casecontrol studies where there is no information concerning the source of patients included in the study. | unable to determine |  |  |  |
| *22. Were study subjects in different intervention groups (trials and cohort studies) or were the cases and controls (case‐control studies) recruited over the same period of time?* For a study which does not specify the time period over which patients were recruited, the question should be answered as unable to determine. | unable to determine |  |  |  |
| *23. Were study subjects randomised to intervention groups?* Studies which state that subjects were randomized should be answered yes except where method of randomisation would not ensure random allocation. For example alternate allocation would score no because it is predictable. |  | no |  |  |
| *24. Was the randomised intervention assignment concealed from both patients and health care staff until recruitment was complete and irrevocable?* All non‐randomised studies should be answered no. If assignment was oncealed from patients but not from staff, it should be answered no. |  | no |  |  |
| *25. Was there adequate adjustment for confounding in the analyses from which the main findings were drawn?* This question should be answered no for trials if: the main conclusions of the study were based on analyses of treatment rather than intention to treat; the distribution of known confounders in the different treatment groups was not described; or the distribution of known confounders differed between the treatment groups but was not taken into account in the analyses. In nonrandomized studies if the effect of the main confounders was not investigated or confounding was demonstrated but no adjustment was made in the final analyses the question should be answered as no. |  | no |  |  |
| *26. Were losses of patients to follow‐up taken into account?* If the numbers of patients lost to follow‐up are not reported, the question should be answered as unable to determine. If the proportion lost to follow‐ up was too small to affect the main findings, the question should be answered yes. | unable to determine |  |  |  |
| **Power** |  |  |  |  |
| *27. Did the study have sufficient power to detect a clinically important effect where the probability value for a difference being due to chance is less than 5%?*Sample sizes have been calculated to detect a difference of x% and y%. |  | no |  | no a priori power analysis was conducted, unable to determine effect size of interested outcome |
| **Total score:** |  |  | 13 |  |
|  |  |  |  |  |
| **Article Author & Name: John-Henderson et al. 2022** | unable to determine | no | yes | **Notes/Justification** |
| **Reporting** | 0 | 0 | 1 |  |
| *1. Is the hypothesis/aim/objective of the study clearly described?* |  |  | yes |  |
| *2. Are the main outcomes to be measured clearly described in the Introduction or Methods section?* If the main outcomes are first mentioned in the Results section, the question should be answered no |  |  | yes |  |
| *3. Are the characteristics of the patients included in the study clearly described ?* In cohort studies and trials, inclusion and/or exclusion criteria should be given. In case‐control studies, a case‐definition and the source for controls should be given. |  |  | yes |  |
| *4. Are the interventions of interest clearly described?*Treatments and placebo (where relevant) that are to be compared should be clearly described. |  |  | yes |  |
| *5. Are the distributions of principal confounders in each group of subjects to be compared clearly described?* A list of principal confounders is provided. |  |  | yes (2) |  |
| *6. Are the main findings of the study clearly described?* Simple outcome data (including denominators and numerators) should be reported for all major findings so that the reader can check the major analyses and conclusions. (This question does not cover statistical tests which are considered below). |  |  | yes |  |
| *7. Does the study provide estimates of the random variability in the data for the main outcomes?* In non normally distributed data the inter‐quartile range of results should be reported. In normally distributed data the standard error, standard deviation or confidence intervals should be reported. If the distribution of the data is not described, it must be assumed that the estimates used were appropriate and the question should be answered yes. |  |  | yes |  |
| *8. Have all important adverse events that may be a consequence of the intervention been reported?* This should be answered yes if the study demonstrates that there was a comprehensive attempt to measure adverse events. (A list of possible adverse events is provided). |  | no |  |  |
| *9. Have the characteristics of patients lost to follow‐up been described?* This should be answered yes where there were no losses to follow‐up or where losses to follow‐up were so small that findings would be unaffected by their inclusion. This should be answered no where a study does not report the number of patients lost to follow‐up. |  |  | yes |  |
| *10. Have actual probability values been reported ( e.g. 0.035 rather than <0.05) for the main outcomes except where the probability value is less than 0.001?* |  |  | yes |  |
| **External Validity** All the following criteria attempt to address the representativeness of the findings of the study and whether they may be generalised to the population from which the study subjects were derived. |  |  |  |  |
| *11. Were the subjects asked to participate in the study representative of the entire population from which they were recruited?* The study must identify the source population for patients and describe how the patients were selected. Patients would be representative if they comprised the entire source population, an unselected sample of consecutive patients, or a random sample. Random sampling is only feasible where a list of all members of the relevant population exists. Where a study does not report the proportion of the source population from which the patients are derived, the question should be answered as unable to determine. | unable to determine |  |  |  |
| *12. Were those subjects who were prepared to participate representative of the entire population from which they were recruited?*The proportion of those asked who agreed should be stated. Validation that the sample was representative would include demonstrating that the distribution of the main confounding factors was the same in the study sample and the source population. | unable to determine |  |  |  |
| *13. Were the staff, places, and facilities where the patients were treated, representative of the treatment the majority of patients receive?* For the question to be answered yes the study should demonstrate that the intervention was representative of that in use in the source population. The question should be answered no if, for example, the intervention was undertaken in a specialist centre unrepresentative of the hospitals most of the source population would attend. | unable to determine |  |  |  |
| **Internal validity – bias** |  |  |  |  |
| *14. Was an attempt made to blind study subjects to the intervention they have received?* For studies where the patients would have no way of knowing which  intervention they received, this should be answered yes. |  | no |  |  |
| *15. Was an attempt made to blind those measuring the main outcomes of the intervention?* |  | no |  |  |
| *16. If any of the results of the study were based on “data dredging”, was this made clear?* Any analyses that had not been planned at the outset of the study should be clearly indicated. If no retrospective unplanned subgroup analyses were reported, then answer yes. |  |  | yes |  |
| *17. In trials and cohort studies, do the analyses adjust for different lengths of follow‐up of patients, or in case‐control studies, is the time period between the intervention and outcome the same for cases and controls?*Where follow‐up was the same for all study patients the answer should yes. If different lengths of follow‐up were adjusted for by, for example, survival analysis the answer should be yes. Studies where differences in follow‐up are ignored should be answered no. |  |  | yes |  |
| 1*8. Were the statistical tests used to assess the main outcomes appropriate?* The statistical techniques used must be appropriate to the data. For example nonparametric methods should be used for small sample sizes. Where little statistical analysis has been undertaken but where there is no evidence of bias, the question should be answered yes. If the distribution of the data (normal or not) is not described it must be assumed that the estimates used were appropriate and the question should be answered yes. |  |  | yes |  |
| *19. Was compliance with the intervention/s reliable*? Where there was non compliance with the allocated treatment or where there was contamination of one group, the question should be answered no. For studies where the effect of any misclassification was likely to bias any association to the null, the question should be answered yes |  |  | yes |  |
| *20. Were the main outcome measures used accurate (valid and reliable)?* For studies where the outcome measures are clearly described, the question should be answered yes. For studies which refer to other work or that demonstrates the outcome measures are accurate, the question should be answered as yes. |  |  | yes |  |
| **Internal validity ‐ confounding (selection bias)** |  |  |  |  |
| *21. Were the patients in different intervention groups (trials and cohort studies) or were the cases and controls (case‐control studies) recruited from the same population?* For example, patients for all comparison groups should be selected from the same hospital. The question should be answered unable to determine for cohort and casecontrol studies where there is no information concerning the source of patients included in the study. | unable to determine |  |  |  |
| *22. Were study subjects in different intervention groups (trials and cohort studies) or were the cases and controls (case‐control studies) recruited over the same period of time?* For a study which does not specify the time period over which patients were recruited, the question should be answered as unable to determine. | unable to determine |  |  |  |
| *23. Were study subjects randomised to intervention groups?* Studies which state that subjects were randomized should be answered yes except where method of randomisation would not ensure random allocation. For example alternate allocation would score no because it is predictable. |  |  | yes |  |
| *24. Was the randomised intervention assignment concealed from both patients and health care staff until recruitment was complete and irrevocable?* All non‐randomised studies should be answered no. If assignment was oncealed from patients but not from staff, it should be answered no. |  | no |  |  |
| *25. Was there adequate adjustment for confounding in the analyses from which the main findings were drawn?* This question should be answered no for trials if: the main conclusions of the study were based on analyses of treatment rather than intention to treat; the distribution of known confounders in the different treatment groups was not described; or the distribution of known confounders differed between the treatment groups but was not taken into account in the analyses. In nonrandomized studies if the effect of the main confounders was not investigated or confounding was demonstrated but no adjustment was made in the final analyses the question should be answered as no. |  | no |  |  |
| *26. Were losses of patients to follow‐up taken into account?* If the numbers of patients lost to follow‐up are not reported, the question should be answered as unable to determine. If the proportion lost to follow‐ up was too small to affect the main findings, the question should be answered yes. | unable to determine |  |  |  |
| **Power** |  |  |  |  |
| *27. Did the study have sufficient power to detect a clinically important effect where the probability value for a difference being due to chance is less than 5%?*Sample sizes have been calculated to detect a difference of x% and y%. |  |  | yes |  |
| **Total score:** |  |  | 17 |  |
|  |  |  |  |  |
| **Article Author & Name: Matsubara et al. 2023** | unable to determine | no | yes | **Notes/Justification** |
| **Reporting** | 0 | 0 | 1 |  |
| *1. Is the hypothesis/aim/objective of the study clearly described?* |  |  | yes |  |
| *2. Are the main outcomes to be measured clearly described in the Introduction or Methods section?* If the main outcomes are first mentioned in the Results section, the question should be answered no |  |  | yes |  |
| *3. Are the characteristics of the patients included in the study clearly described ?* In cohort studies and trials, inclusion and/or exclusion criteria should be given. In case‐control studies, a case‐definition and the source for controls should be given. |  |  | yes |  |
| *4. Are the interventions of interest clearly described?*Treatments and placebo (where relevant) that are to be compared should be clearly described. |  |  | yes |  |
| *5. Are the distributions of principal confounders in each group of subjects to be compared clearly described?* A list of principal confounders is provided. |  | no |  |  |
| *6. Are the main findings of the study clearly described?* Simple outcome data (including denominators and numerators) should be reported for all major findings so that the reader can check the major analyses and conclusions. (This question does not cover statistical tests which are considered below). |  |  | yes |  |
| *7. Does the study provide estimates of the random variability in the data for the main outcomes?* In non normally distributed data the inter‐quartile range of results should be reported. In normally distributed data the standard error, standard deviation or confidence intervals should be reported. If the distribution of the data is not described, it must be assumed that the estimates used were appropriate and the question should be answered yes. |  |  | yes |  |
| *8. Have all important adverse events that may be a consequence of the intervention been reported?* This should be answered yes if the study demonstrates that there was a comprehensive attempt to measure adverse events. (A list of possible adverse events is provided). |  | no |  |  |
| *9. Have the characteristics of patients lost to follow‐up been described?* This should be answered yes where there were no losses to follow‐up or where losses to follow‐up were so small that findings would be unaffected by their inclusion. This should be answered no where a study does not report the number of patients lost to follow‐up. | unable to determine |  |  |  |
| *10. Have actual probability values been reported ( e.g. 0.035 rather than <0.05) for the main outcomes except where the probability value is less than 0.001?* |  | no |  |  |
| **External Validity** All the following criteria attempt to address the representativeness of the findings of the study and whether they may be generalised to the population from which the study subjects were derived. |  |  |  |  |
| *11. Were the subjects asked to participate in the study representative of the entire population from which they were recruited?* The study must identify the source population for patients and describe how the patients were selected. Patients would be representative if they comprised the entire source population, an unselected sample of consecutive patients, or a random sample. Random sampling is only feasible where a list of all members of the relevant population exists. Where a study does not report the proportion of the source population from which the patients are derived, the question should be answered as unable to determine. | unable to determine |  |  |  |
| *12. Were those subjects who were prepared to participate representative of the entire population from which they were recruited?*The proportion of those asked who agreed should be stated. Validation that the sample was representative would include demonstrating that the distribution of the main confounding factors was the same in the study sample and the source population. | unable to determine |  |  |  |
| *13. Were the staff, places, and facilities where the patients were treated, representative of the treatment the majority of patients receive?* For the question to be answered yes the study should demonstrate that the intervention was representative of that in use in the source population. The question should be answered no if, for example, the intervention was undertaken in a specialist centre unrepresentative of the hospitals most of the source population would attend. | unable to determine |  |  |  |
| **Internal validity – bias** |  |  |  |  |
| *14. Was an attempt made to blind study subjects to the intervention they have received?* For studies where the patients would have no way of knowing which  intervention they received, this should be answered yes. |  | no |  |  |
| *15. Was an attempt made to blind those measuring the main outcomes of the intervention?* |  | no |  |  |
| *16. If any of the results of the study were based on “data dredging”, was this made clear?* Any analyses that had not been planned at the outset of the study should be clearly indicated. If no retrospective unplanned subgroup analyses were reported, then answer yes. |  |  | yes |  |
| *17. In trials and cohort studies, do the analyses adjust for different lengths of follow‐up of patients, or in case‐control studies, is the time period between the intervention and outcome the same for cases and controls?*Where follow‐up was the same for all study patients the answer should yes. If different lengths of follow‐up were adjusted for by, for example, survival analysis the answer should be yes. Studies where differences in follow‐up are ignored should be answered no. | unable to determine |  |  |  |
| 1*8. Were the statistical tests used to assess the main outcomes appropriate?* The statistical techniques used must be appropriate to the data. For example nonparametric methods should be used for small sample sizes. Where little statistical analysis has been undertaken but where there is no evidence of bias, the question should be answered yes. If the distribution of the data (normal or not) is not described it must be assumed that the estimates used were appropriate and the question should be answered yes. |  |  | yes |  |
| *19. Was compliance with the intervention/s reliable*? Where there was non compliance with the allocated treatment or where there was contamination of one group, the question should be answered no. For studies where the effect of any misclassification was likely to bias any association to the null, the question should be answered yes |  |  | yes |  |
| *20. Were the main outcome measures used accurate (valid and reliable)?* For studies where the outcome measures are clearly described, the question should be answered yes. For studies which refer to other work or that demonstrates the outcome measures are accurate, the question should be answered as yes. |  |  | yes |  |
| **Internal validity ‐ confounding (selection bias)** |  |  |  |  |
| *21. Were the patients in different intervention groups (trials and cohort studies) or were the cases and controls (case‐control studies) recruited from the same population?* For example, patients for all comparison groups should be selected from the same hospital. The question should be answered unable to determine for cohort and casecontrol studies where there is no information concerning the source of patients included in the study. | unable to determine |  |  |  |
| *22. Were study subjects in different intervention groups (trials and cohort studies) or were the cases and controls (case‐control studies) recruited over the same period of time?* For a study which does not specify the time period over which patients were recruited, the question should be answered as unable to determine. | unable to determine |  |  |  |
| *23. Were study subjects randomised to intervention groups?* Studies which state that subjects were randomized should be answered yes except where method of randomisation would not ensure random allocation. For example alternate allocation would score no because it is predictable. |  | no |  |  |
| *24. Was the randomised intervention assignment concealed from both patients and health care staff until recruitment was complete and irrevocable?* All non‐randomised studies should be answered no. If assignment was oncealed from patients but not from staff, it should be answered no. |  | no |  |  |
| *25. Was there adequate adjustment for confounding in the analyses from which the main findings were drawn?* This question should be answered no for trials if: the main conclusions of the study were based on analyses of treatment rather than intention to treat; the distribution of known confounders in the different treatment groups was not described; or the distribution of known confounders differed between the treatment groups but was not taken into account in the analyses. In nonrandomized studies if the effect of the main confounders was not investigated or confounding was demonstrated but no adjustment was made in the final analyses the question should be answered as no. |  | no |  |  |
| *26. Were losses of patients to follow‐up taken into account?* If the numbers of patients lost to follow‐up are not reported, the question should be answered as unable to determine. If the proportion lost to follow‐ up was too small to affect the main findings, the question should be answered yes. | unable to determine |  |  |  |
| **Power** |  |  |  |  |
| *27. Did the study have sufficient power to detect a clinically important effect where the probability value for a difference being due to chance is less than 5%?*Sample sizes have been calculated to detect a difference of x% and y%. |  | no |  | no a priori power analysis was conducted, unable to determine effect size of interested outcome |
| **Total score:** |  |  | 10 |  |
|  |  |  |  |  |
| **Article Author & Name: Matzner et al. 2013** | unable to determine | no | yes | **Notes/Justification** |
| **Reporting** | 0 | 0 | 1 |  |
| *1. Is the hypothesis/aim/objective of the study clearly described?* |  |  | yes |  |
| *2. Are the main outcomes to be measured clearly described in the Introduction or Methods section?* If the main outcomes are first mentioned in the Results section, the question should be answered no |  |  | yes |  |
| *3. Are the characteristics of the patients included in the study clearly described ?* In cohort studies and trials, inclusion and/or exclusion criteria should be given. In case‐control studies, a case‐definition and the source for controls should be given. |  |  | yes |  |
| *4. Are the interventions of interest clearly described?*Treatments and placebo (where relevant) that are to be compared should be clearly described. |  |  | yes |  |
| *5. Are the distributions of principal confounders in each group of subjects to be compared clearly described?* A list of principal confounders is provided. | unable to determine |  |  |  |
| *6. Are the main findings of the study clearly described?* Simple outcome data (including denominators and numerators) should be reported for all major findings so that the reader can check the major analyses and conclusions. (This question does not cover statistical tests which are considered below). |  |  | yes |  |
| *7. Does the study provide estimates of the random variability in the data for the main outcomes?* In non normally distributed data the inter‐quartile range of results should be reported. In normally distributed data the standard error, standard deviation or confidence intervals should be reported. If the distribution of the data is not described, it must be assumed that the estimates used were appropriate and the question should be answered yes. |  | no |  |  |
| *8. Have all important adverse events that may be a consequence of the intervention been reported?* This should be answered yes if the study demonstrates that there was a comprehensive attempt to measure adverse events. (A list of possible adverse events is provided). |  | no |  |  |
| *9. Have the characteristics of patients lost to follow‐up been described?* This should be answered yes where there were no losses to follow‐up or where losses to follow‐up were so small that findings would be unaffected by their inclusion. This should be answered no where a study does not report the number of patients lost to follow‐up. | unable to determine |  |  |  |
| *10. Have actual probability values been reported ( e.g. 0.035 rather than <0.05) for the main outcomes except where the probability value is less than 0.001?* |  | no |  |  |
| **External Validity** All the following criteria attempt to address the representativeness of the findings of the study and whether they may be generalised to the population from which the study subjects were derived. |  |  |  |  |
| *11. Were the subjects asked to participate in the study representative of the entire population from which they were recruited?* The study must identify the source population for patients and describe how the patients were selected. Patients would be representative if they comprised the entire source population, an unselected sample of consecutive patients, or a random sample. Random sampling is only feasible where a list of all members of the relevant population exists. Where a study does not report the proportion of the source population from which the patients are derived, the question should be answered as unable to determine. |  |  | yes |  |
| *12. Were those subjects who were prepared to participate representative of the entire population from which they were recruited?*The proportion of those asked who agreed should be stated. Validation that the sample was representative would include demonstrating that the distribution of the main confounding factors was the same in the study sample and the source population. | unable to determine |  |  |  |
| *13. Were the staff, places, and facilities where the patients were treated, representative of the treatment the majority of patients receive?* For the question to be answered yes the study should demonstrate that the intervention was representative of that in use in the source population. The question should be answered no if, for example, the intervention was undertaken in a specialist centre unrepresentative of the hospitals most of the source population would attend. | unable to determine |  |  |  |
| **Internal validity – bias** |  |  |  |  |
| *14. Was an attempt made to blind study subjects to the intervention they have received?* For studies where the patients would have no way of knowing which  intervention they received, this should be answered yes. |  | no |  |  |
| *15. Was an attempt made to blind those measuring the main outcomes of the intervention?* |  | no |  |  |
| *16. If any of the results of the study were based on “data dredging”, was this made clear?* Any analyses that had not been planned at the outset of the study should be clearly indicated. If no retrospective unplanned subgroup analyses were reported, then answer yes. |  |  | yes |  |
| *17. In trials and cohort studies, do the analyses adjust for different lengths of follow‐up of patients, or in case‐control studies, is the time period between the intervention and outcome the same for cases and controls?*Where follow‐up was the same for all study patients the answer should yes. If different lengths of follow‐up were adjusted for by, for example, survival analysis the answer should be yes. Studies where differences in follow‐up are ignored should be answered no. |  |  | yes |  |
| 1*8. Were the statistical tests used to assess the main outcomes appropriate?* The statistical techniques used must be appropriate to the data. For example nonparametric methods should be used for small sample sizes. Where little statistical analysis has been undertaken but where there is no evidence of bias, the question should be answered yes. If the distribution of the data (normal or not) is not described it must be assumed that the estimates used were appropriate and the question should be answered yes. |  |  | yes |  |
| *19. Was compliance with the intervention/s reliable*? Where there was non compliance with the allocated treatment or where there was contamination of one group, the question should be answered no. For studies where the effect of any misclassification was likely to bias any association to the null, the question should be answered yes |  |  | yes |  |
| *20. Were the main outcome measures used accurate (valid and reliable)?* For studies where the outcome measures are clearly described, the question should be answered yes. For studies which refer to other work or that demonstrates the outcome measures are accurate, the question should be answered as yes. |  |  | yes |  |
| **Internal validity ‐ confounding (selection bias)** |  |  |  |  |
| *21. Were the patients in different intervention groups (trials and cohort studies) or were the cases and controls (case‐control studies) recruited from the same population?* For example, patients for all comparison groups should be selected from the same hospital. The question should be answered unable to determine for cohort and casecontrol studies where there is no information concerning the source of patients included in the study. |  |  | yes |  |
| *22. Were study subjects in different intervention groups (trials and cohort studies) or were the cases and controls (case‐control studies) recruited over the same period of time?* For a study which does not specify the time period over which patients were recruited, the question should be answered as unable to determine. | unable to determine |  |  |  |
| *23. Were study subjects randomised to intervention groups?* Studies which state that subjects were randomized should be answered yes except where method of randomisation would not ensure random allocation. For example alternate allocation would score no because it is predictable. |  |  | yes |  |
| *24. Was the randomised intervention assignment concealed from both patients and health care staff until recruitment was complete and irrevocable?* All non‐randomised studies should be answered no. If assignment was oncealed from patients but not from staff, it should be answered no. |  | no |  |  |
| *25. Was there adequate adjustment for confounding in the analyses from which the main findings were drawn?* This question should be answered no for trials if: the main conclusions of the study were based on analyses of treatment rather than intention to treat; the distribution of known confounders in the different treatment groups was not described; or the distribution of known confounders differed between the treatment groups but was not taken into account in the analyses. In nonrandomized studies if the effect of the main confounders was not investigated or confounding was demonstrated but no adjustment was made in the final analyses the question should be answered as no. |  | no |  |  |
| *26. Were losses of patients to follow‐up taken into account?* If the numbers of patients lost to follow‐up are not reported, the question should be answered as unable to determine. If the proportion lost to follow‐ up was too small to affect the main findings, the question should be answered yes. | unable to determine |  |  |  |
| **Power** |  |  |  |  |
| *27. Did the study have sufficient power to detect a clinically important effect where the probability value for a difference being due to chance is less than 5%?*Sample sizes have been calculated to detect a difference of x% and y%. |  | no |  | no a priori power analysis was conducted, unable to determine effect size of interested outcome |
| **Total score:** |  |  | 13 |  |
|  |  |  |  |  |
| **Article Author & Name: Meier-Ewert et al. 2004** | unable to determine | no | yes | **Notes/Justification** |
| **Reporting** | 0 | 0 | 1 |  |
| *1. Is the hypothesis/aim/objective of the study clearly described?* |  |  | yes |  |
| *2. Are the main outcomes to be measured clearly described in the Introduction or Methods section?* If the main outcomes are first mentioned in the Results section, the question should be answered no |  |  | yes |  |
| *3. Are the characteristics of the patients included in the study clearly described ?* In cohort studies and trials, inclusion and/or exclusion criteria should be given. In case‐control studies, a case‐definition and the source for controls should be given. |  |  | yes |  |
| *4. Are the interventions of interest clearly described?*Treatments and placebo (where relevant) that are to be compared should be clearly described. |  |  | yes |  |
| *5. Are the distributions of principal confounders in each group of subjects to be compared clearly described?* A list of principal confounders is provided. |  |  | yes (2) |  |
| *6. Are the main findings of the study clearly described?* Simple outcome data (including denominators and numerators) should be reported for all major findings so that the reader can check the major analyses and conclusions. (This question does not cover statistical tests which are considered below). |  |  | yes |  |
| *7. Does the study provide estimates of the random variability in the data for the main outcomes?* In non normally distributed data the inter‐quartile range of results should be reported. In normally distributed data the standard error, standard deviation or confidence intervals should be reported. If the distribution of the data is not described, it must be assumed that the estimates used were appropriate and the question should be answered yes. |  |  | yes |  |
| *8. Have all important adverse events that may be a consequence of the intervention been reported?* This should be answered yes if the study demonstrates that there was a comprehensive attempt to measure adverse events. (A list of possible adverse events is provided). |  | no |  |  |
| *9. Have the characteristics of patients lost to follow‐up been described?* This should be answered yes where there were no losses to follow‐up or where losses to follow‐up were so small that findings would be unaffected by their inclusion. This should be answered no where a study does not report the number of patients lost to follow‐up. | unable to determine |  |  |  |
| *10. Have actual probability values been reported ( e.g. 0.035 rather than <0.05) for the main outcomes except where the probability value is less than 0.001?* |  |  | yes |  |
| **External Validity** All the following criteria attempt to address the representativeness of the findings of the study and whether they may be generalised to the population from which the study subjects were derived. |  |  |  |  |
| *11. Were the subjects asked to participate in the study representative of the entire population from which they were recruited?* The study must identify the source population for patients and describe how the patients were selected. Patients would be representative if they comprised the entire source population, an unselected sample of consecutive patients, or a random sample. Random sampling is only feasible where a list of all members of the relevant population exists. Where a study does not report the proportion of the source population from which the patients are derived, the question should be answered as unable to determine. | unable to determine |  |  |  |
| *12. Were those subjects who were prepared to participate representative of the entire population from which they were recruited?*The proportion of those asked who agreed should be stated. Validation that the sample was representative would include demonstrating that the distribution of the main confounding factors was the same in the study sample and the source population. | unable to determine |  |  |  |
| *13. Were the staff, places, and facilities where the patients were treated, representative of the treatment the majority of patients receive?* For the question to be answered yes the study should demonstrate that the intervention was representative of that in use in the source population. The question should be answered no if, for example, the intervention was undertaken in a specialist centre unrepresentative of the hospitals most of the source population would attend. | unable to determine |  |  |  |
| **Internal validity – bias** |  |  |  |  |
| *14. Was an attempt made to blind study subjects to the intervention they have received?* For studies where the patients would have no way of knowing which  intervention they received, this should be answered yes. |  | no |  |  |
| *15. Was an attempt made to blind those measuring the main outcomes of the intervention?* |  | no |  |  |
| *16. If any of the results of the study were based on “data dredging”, was this made clear?* Any analyses that had not been planned at the outset of the study should be clearly indicated. If no retrospective unplanned subgroup analyses were reported, then answer yes. |  |  | yes |  |
| *17. In trials and cohort studies, do the analyses adjust for different lengths of follow‐up of patients, or in case‐control studies, is the time period between the intervention and outcome the same for cases and controls?*Where follow‐up was the same for all study patients the answer should yes. If different lengths of follow‐up were adjusted for by, for example, survival analysis the answer should be yes. Studies where differences in follow‐up are ignored should be answered no. |  |  | yes |  |
| 1*8. Were the statistical tests used to assess the main outcomes appropriate?* The statistical techniques used must be appropriate to the data. For example nonparametric methods should be used for small sample sizes. Where little statistical analysis has been undertaken but where there is no evidence of bias, the question should be answered yes. If the distribution of the data (normal or not) is not described it must be assumed that the estimates used were appropriate and the question should be answered yes. |  |  | yes |  |
| *19. Was compliance with the intervention/s reliable*? Where there was non compliance with the allocated treatment or where there was contamination of one group, the question should be answered no. For studies where the effect of any misclassification was likely to bias any association to the null, the question should be answered yes |  |  | yes |  |
| *20. Were the main outcome measures used accurate (valid and reliable)?* For studies where the outcome measures are clearly described, the question should be answered yes. For studies which refer to other work or that demonstrates the outcome measures are accurate, the question should be answered as yes. |  |  | yes |  |
| **Internal validity ‐ confounding (selection bias)** |  |  |  |  |
| *21. Were the patients in different intervention groups (trials and cohort studies) or were the cases and controls (case‐control studies) recruited from the same population?* For example, patients for all comparison groups should be selected from the same hospital. The question should be answered unable to determine for cohort and casecontrol studies where there is no information concerning the source of patients included in the study. | unable to determine |  |  |  |
| *22. Were study subjects in different intervention groups (trials and cohort studies) or were the cases and controls (case‐control studies) recruited over the same period of time?* For a study which does not specify the time period over which patients were recruited, the question should be answered as unable to determine. | unable to determine |  |  |  |
| *23. Were study subjects randomised to intervention groups?* Studies which state that subjects were randomized should be answered yes except where method of randomisation would not ensure random allocation. For example alternate allocation would score no because it is predictable. |  |  | yes |  |
| *24. Was the randomised intervention assignment concealed from both patients and health care staff until recruitment was complete and irrevocable?* All non‐randomised studies should be answered no. If assignment was oncealed from patients but not from staff, it should be answered no. | unable to determine |  |  |  |
| *25. Was there adequate adjustment for confounding in the analyses from which the main findings were drawn?* This question should be answered no for trials if: the main conclusions of the study were based on analyses of treatment rather than intention to treat; the distribution of known confounders in the different treatment groups was not described; or the distribution of known confounders differed between the treatment groups but was not taken into account in the analyses. In nonrandomized studies if the effect of the main confounders was not investigated or confounding was demonstrated but no adjustment was made in the final analyses the question should be answered as no. |  | no |  |  |
| *26. Were losses of patients to follow‐up taken into account?* If the numbers of patients lost to follow‐up are not reported, the question should be answered as unable to determine. If the proportion lost to follow‐ up was too small to affect the main findings, the question should be answered yes. | unable to determine |  |  |  |
| **Power** |  |  |  |  |
| *27. Did the study have sufficient power to detect a clinically important effect where the probability value for a difference being due to chance is less than 5%?*Sample sizes have been calculated to detect a difference of x% and y%. |  | no |  |  |
| **Total score:** |  |  | 15 |  |
|  |  |  |  |  |
| **Article Author & Name: Mejri et al. 2017** | unable to determine | no | yes | **Notes/Justification** |
| **Reporting** | 0 | 0 | 1 |  |
| *1. Is the hypothesis/aim/objective of the study clearly described?* |  |  | yes |  |
| *2. Are the main outcomes to be measured clearly described in the Introduction or Methods section?* If the main outcomes are first mentioned in the Results section, the question should be answered no |  |  | yes |  |
| *3. Are the characteristics of the patients included in the study clearly described ?* In cohort studies and trials, inclusion and/or exclusion criteria should be given. In case‐control studies, a case‐definition and the source for controls should be given. |  |  | yes |  |
| *4. Are the interventions of interest clearly described?*Treatments and placebo (where relevant) that are to be compared should be clearly described. |  |  | yes |  |
| *5. Are the distributions of principal confounders in each group of subjects to be compared clearly described?* A list of principal confounders is provided. |  |  | yes (2) |  |
| *6. Are the main findings of the study clearly described?* Simple outcome data (including denominators and numerators) should be reported for all major findings so that the reader can check the major analyses and conclusions. (This question does not cover statistical tests which are considered below). |  |  | yes |  |
| *7. Does the study provide estimates of the random variability in the data for the main outcomes?* In non normally distributed data the inter‐quartile range of results should be reported. In normally distributed data the standard error, standard deviation or confidence intervals should be reported. If the distribution of the data is not described, it must be assumed that the estimates used were appropriate and the question should be answered yes. |  |  | yes |  |
| *8. Have all important adverse events that may be a consequence of the intervention been reported?* This should be answered yes if the study demonstrates that there was a comprehensive attempt to measure adverse events. (A list of possible adverse events is provided). |  | no |  |  |
| *9. Have the characteristics of patients lost to follow‐up been described?* This should be answered yes where there were no losses to follow‐up or where losses to follow‐up were so small that findings would be unaffected by their inclusion. This should be answered no where a study does not report the number of patients lost to follow‐up. | unable to determine |  |  |  |
| *10. Have actual probability values been reported ( e.g. 0.035 rather than <0.05) for the main outcomes except where the probability value is less than 0.001?* |  | no |  |  |
| **External Validity** All the following criteria attempt to address the representativeness of the findings of the study and whether they may be generalised to the population from which the study subjects were derived. |  |  |  |  |
| *11. Were the subjects asked to participate in the study representative of the entire population from which they were recruited?* The study must identify the source population for patients and describe how the patients were selected. Patients would be representative if they comprised the entire source population, an unselected sample of consecutive patients, or a random sample. Random sampling is only feasible where a list of all members of the relevant population exists. Where a study does not report the proportion of the source population from which the patients are derived, the question should be answered as unable to determine. |  |  | yes |  |
| *12. Were those subjects who were prepared to participate representative of the entire population from which they were recruited?*The proportion of those asked who agreed should be stated. Validation that the sample was representative would include demonstrating that the distribution of the main confounding factors was the same in the study sample and the source population. | unable to determine |  |  |  |
| *13. Were the staff, places, and facilities where the patients were treated, representative of the treatment the majority of patients receive?* For the question to be answered yes the study should demonstrate that the intervention was representative of that in use in the source population. The question should be answered no if, for example, the intervention was undertaken in a specialist centre unrepresentative of the hospitals most of the source population would attend. | unable to determine |  |  |  |
| **Internal validity – bias** |  |  |  |  |
| *14. Was an attempt made to blind study subjects to the intervention they have received?* For studies where the patients would have no way of knowing which  intervention they received, this should be answered yes. |  | no |  |  |
| *15. Was an attempt made to blind those measuring the main outcomes of the intervention?* |  | no |  |  |
| *16. If any of the results of the study were based on “data dredging”, was this made clear?* Any analyses that had not been planned at the outset of the study should be clearly indicated. If no retrospective unplanned subgroup analyses were reported, then answer yes. |  |  | yes |  |
| *17. In trials and cohort studies, do the analyses adjust for different lengths of follow‐up of patients, or in case‐control studies, is the time period between the intervention and outcome the same for cases and controls?*Where follow‐up was the same for all study patients the answer should yes. If different lengths of follow‐up were adjusted for by, for example, survival analysis the answer should be yes. Studies where differences in follow‐up are ignored should be answered no. | unable to determine |  |  |  |
| 1*8. Were the statistical tests used to assess the main outcomes appropriate?* The statistical techniques used must be appropriate to the data. For example nonparametric methods should be used for small sample sizes. Where little statistical analysis has been undertaken but where there is no evidence of bias, the question should be answered yes. If the distribution of the data (normal or not) is not described it must be assumed that the estimates used were appropriate and the question should be answered yes. |  |  | yes |  |
| *19. Was compliance with the intervention/s reliable*? Where there was non compliance with the allocated treatment or where there was contamination of one group, the question should be answered no. For studies where the effect of any misclassification was likely to bias any association to the null, the question should be answered yes |  |  | yes |  |
| *20. Were the main outcome measures used accurate (valid and reliable)?* For studies where the outcome measures are clearly described, the question should be answered yes. For studies which refer to other work or that demonstrates the outcome measures are accurate, the question should be answered as yes. |  |  | yes |  |
| **Internal validity ‐ confounding (selection bias)** |  |  |  |  |
| *21. Were the patients in different intervention groups (trials and cohort studies) or were the cases and controls (case‐control studies) recruited from the same population?* For example, patients for all comparison groups should be selected from the same hospital. The question should be answered unable to determine for cohort and casecontrol studies where there is no information concerning the source of patients included in the study. |  |  | yes |  |
| *22. Were study subjects in different intervention groups (trials and cohort studies) or were the cases and controls (case‐control studies) recruited over the same period of time?* For a study which does not specify the time period over which patients were recruited, the question should be answered as unable to determine. | unable to determine |  |  |  |
| *23. Were study subjects randomised to intervention groups?* Studies which state that subjects were randomized should be answered yes except where method of randomisation would not ensure random allocation. For example alternate allocation would score no because it is predictable. |  | no |  |  |
| *24. Was the randomised intervention assignment concealed from both patients and health care staff until recruitment was complete and irrevocable?* All non‐randomised studies should be answered no. If assignment was oncealed from patients but not from staff, it should be answered no. |  | no |  |  |
| *25. Was there adequate adjustment for confounding in the analyses from which the main findings were drawn?* This question should be answered no for trials if: the main conclusions of the study were based on analyses of treatment rather than intention to treat; the distribution of known confounders in the different treatment groups was not described; or the distribution of known confounders differed between the treatment groups but was not taken into account in the analyses. In nonrandomized studies if the effect of the main confounders was not investigated or confounding was demonstrated but no adjustment was made in the final analyses the question should be answered as no. |  | no |  |  |
| *26. Were losses of patients to follow‐up taken into account?* If the numbers of patients lost to follow‐up are not reported, the question should be answered as unable to determine. If the proportion lost to follow‐ up was too small to affect the main findings, the question should be answered yes. | unable to determine |  |  |  |
| **Power** |  |  |  |  |
| *27. Did the study have sufficient power to detect a clinically important effect where the probability value for a difference being due to chance is less than 5%?*Sample sizes have been calculated to detect a difference of x% and y%. |  |  | yes | no a priori power analysis was conducted, adequate effect size |
| **Total score:** |  |  | 15 |  |
|  |  |  |  |  |
| **Article Author & Name: Pejovic et al. 2013** | unable to determine | no | yes | **Notes/Justification** |
| **Reporting** | 0 | 0 | 1 |  |
| *1. Is the hypothesis/aim/objective of the study clearly described?* |  |  | yes |  |
| *2. Are the main outcomes to be measured clearly described in the Introduction or Methods section?* If the main outcomes are first mentioned in the Results section, the question should be answered no |  |  | yes |  |
| *3. Are the characteristics of the patients included in the study clearly described ?* In cohort studies and trials, inclusion and/or exclusion criteria should be given. In case‐control studies, a case‐definition and the source for controls should be given. |  |  | yes |  |
| *4. Are the interventions of interest clearly described?*Treatments and placebo (where relevant) that are to be compared should be clearly described. |  |  | yes |  |
| *5. Are the distributions of principal confounders in each group of subjects to be compared clearly described?* A list of principal confounders is provided. |  |  | yes (2) |  |
| *6. Are the main findings of the study clearly described?* Simple outcome data (including denominators and numerators) should be reported for all major findings so that the reader can check the major analyses and conclusions. (This question does not cover statistical tests which are considered below). |  |  | yes |  |
| *7. Does the study provide estimates of the random variability in the data for the main outcomes?* In non normally distributed data the inter‐quartile range of results should be reported. In normally distributed data the standard error, standard deviation or confidence intervals should be reported. If the distribution of the data is not described, it must be assumed that the estimates used were appropriate and the question should be answered yes. |  |  | yes |  |
| *8. Have all important adverse events that may be a consequence of the intervention been reported?* This should be answered yes if the study demonstrates that there was a comprehensive attempt to measure adverse events. (A list of possible adverse events is provided). |  | no |  |  |
| *9. Have the characteristics of patients lost to follow‐up been described?* This should be answered yes where there were no losses to follow‐up or where losses to follow‐up were so small that findings would be unaffected by their inclusion. This should be answered no where a study does not report the number of patients lost to follow‐up. |  | no |  |  |
| *10. Have actual probability values been reported ( e.g. 0.035 rather than <0.05) for the main outcomes except where the probability value is less than 0.001?* |  |  | yes |  |
| **External Validity** All the following criteria attempt to address the representativeness of the findings of the study and whether they may be generalised to the population from which the study subjects were derived. |  |  |  |  |
| *11. Were the subjects asked to participate in the study representative of the entire population from which they were recruited?* The study must identify the source population for patients and describe how the patients were selected. Patients would be representative if they comprised the entire source population, an unselected sample of consecutive patients, or a random sample. Random sampling is only feasible where a list of all members of the relevant population exists. Where a study does not report the proportion of the source population from which the patients are derived, the question should be answered as unable to determine. |  |  | yes |  |
| *12. Were those subjects who were prepared to participate representative of the entire population from which they were recruited?*The proportion of those asked who agreed should be stated. Validation that the sample was representative would include demonstrating that the distribution of the main confounding factors was the same in the study sample and the source population. | unable to determine |  |  |  |
| *13. Were the staff, places, and facilities where the patients were treated, representative of the treatment the majority of patients receive?* For the question to be answered yes the study should demonstrate that the intervention was representative of that in use in the source population. The question should be answered no if, for example, the intervention was undertaken in a specialist centre unrepresentative of the hospitals most of the source population would attend. |  | no |  |  |
| **Internal validity – bias** |  |  |  |  |
| *14. Was an attempt made to blind study subjects to the intervention they have received?* For studies where the patients would have no way of knowing which  intervention they received, this should be answered yes. |  | no |  |  |
| *15. Was an attempt made to blind those measuring the main outcomes of the intervention?* |  | no |  |  |
| *16. If any of the results of the study were based on “data dredging”, was this made clear?* Any analyses that had not been planned at the outset of the study should be clearly indicated. If no retrospective unplanned subgroup analyses were reported, then answer yes. |  |  | yes |  |
| *17. In trials and cohort studies, do the analyses adjust for different lengths of follow‐up of patients, or in case‐control studies, is the time period between the intervention and outcome the same for cases and controls?*Where follow‐up was the same for all study patients the answer should yes. If different lengths of follow‐up were adjusted for by, for example, survival analysis the answer should be yes. Studies where differences in follow‐up are ignored should be answered no. |  |  | yes |  |
| 1*8. Were the statistical tests used to assess the main outcomes appropriate?* The statistical techniques used must be appropriate to the data. For example nonparametric methods should be used for small sample sizes. Where little statistical analysis has been undertaken but where there is no evidence of bias, the question should be answered yes. If the distribution of the data (normal or not) is not described it must be assumed that the estimates used were appropriate and the question should be answered yes. |  |  | yes |  |
| *19. Was compliance with the intervention/s reliable*? Where there was non compliance with the allocated treatment or where there was contamination of one group, the question should be answered no. For studies where the effect of any misclassification was likely to bias any association to the null, the question should be answered yes |  |  | yes |  |
| *20. Were the main outcome measures used accurate (valid and reliable)?* For studies where the outcome measures are clearly described, the question should be answered yes. For studies which refer to other work or that demonstrates the outcome measures are accurate, the question should be answered as yes. |  |  | yes |  |
| **Internal validity ‐ confounding (selection bias)** |  |  |  |  |
| *21. Were the patients in different intervention groups (trials and cohort studies) or were the cases and controls (case‐control studies) recruited from the same population?* For example, patients for all comparison groups should be selected from the same hospital. The question should be answered unable to determine for cohort and casecontrol studies where there is no information concerning the source of patients included in the study. |  |  | yes |  |
| *22. Were study subjects in different intervention groups (trials and cohort studies) or were the cases and controls (case‐control studies) recruited over the same period of time?* For a study which does not specify the time period over which patients were recruited, the question should be answered as unable to determine. | unable to determine |  |  |  |
| *23. Were study subjects randomised to intervention groups?* Studies which state that subjects were randomized should be answered yes except where method of randomisation would not ensure random allocation. For example alternate allocation would score no because it is predictable. |  | no |  |  |
| *24. Was the randomised intervention assignment concealed from both patients and health care staff until recruitment was complete and irrevocable?* All non‐randomised studies should be answered no. If assignment was oncealed from patients but not from staff, it should be answered no. |  | no |  |  |
| *25. Was there adequate adjustment for confounding in the analyses from which the main findings were drawn?* This question should be answered no for trials if: the main conclusions of the study were based on analyses of treatment rather than intention to treat; the distribution of known confounders in the different treatment groups was not described; or the distribution of known confounders differed between the treatment groups but was not taken into account in the analyses. In nonrandomized studies if the effect of the main confounders was not investigated or confounding was demonstrated but no adjustment was made in the final analyses the question should be answered as no. |  |  | yes |  |
| *26. Were losses of patients to follow‐up taken into account?* If the numbers of patients lost to follow‐up are not reported, the question should be answered as unable to determine. If the proportion lost to follow‐ up was too small to affect the main findings, the question should be answered yes. | unable to determine |  |  |  |
| **Power** |  |  |  |  |
| *27. Did the study have sufficient power to detect a clinically important effect where the probability value for a difference being due to chance is less than 5%?*Sample sizes have been calculated to detect a difference of x% and y%. |  | no |  | no a priori power analysis was conducted, unable to determine effect size of interested outcome |
| **Total score:** |  |  | 17 |  |
|  |  |  |  |  |
| **Article Author & Name: Redwine et al. 2000** | unable to determine | no | yes | **Notes/Justification** |
| **Reporting** | 0 | 0 | 1 |  |
| *1. Is the hypothesis/aim/objective of the study clearly described?* |  |  | yes |  |
| *2. Are the main outcomes to be measured clearly described in the Introduction or Methods section?* If the main outcomes are first mentioned in the Results section, the question should be answered no |  |  | yes |  |
| *3. Are the characteristics of the patients included in the study clearly described ?* In cohort studies and trials, inclusion and/or exclusion criteria should be given. In case‐control studies, a case‐definition and the source for controls should be given. |  |  | yes |  |
| *4. Are the interventions of interest clearly described?*Treatments and placebo (where relevant) that are to be compared should be clearly described. |  |  | yes |  |
| *5. Are the distributions of principal confounders in each group of subjects to be compared clearly described?* A list of principal confounders is provided. |  |  | yes (2) |  |
| *6. Are the main findings of the study clearly described?* Simple outcome data (including denominators and numerators) should be reported for all major findings so that the reader can check the major analyses and conclusions. (This question does not cover statistical tests which are considered below). |  |  | yes |  |
| *7. Does the study provide estimates of the random variability in the data for the main outcomes?* In non normally distributed data the inter‐quartile range of results should be reported. In normally distributed data the standard error, standard deviation or confidence intervals should be reported. If the distribution of the data is not described, it must be assumed that the estimates used were appropriate and the question should be answered yes. |  |  | yes |  |
| *8. Have all important adverse events that may be a consequence of the intervention been reported?* This should be answered yes if the study demonstrates that there was a comprehensive attempt to measure adverse events. (A list of possible adverse events is provided). |  | no |  |  |
| *9. Have the characteristics of patients lost to follow‐up been described?* This should be answered yes where there were no losses to follow‐up or where losses to follow‐up were so small that findings would be unaffected by their inclusion. This should be answered no where a study does not report the number of patients lost to follow‐up. |  | no |  |  |
| *10. Have actual probability values been reported ( e.g. 0.035 rather than <0.05) for the main outcomes except where the probability value is less than 0.001?* |  |  | yes |  |
| **External Validity** All the following criteria attempt to address the representativeness of the findings of the study and whether they may be generalised to the population from which the study subjects were derived. |  |  |  |  |
| *11. Were the subjects asked to participate in the study representative of the entire population from which they were recruited?* The study must identify the source population for patients and describe how the patients were selected. Patients would be representative if they comprised the entire source population, an unselected sample of consecutive patients, or a random sample. Random sampling is only feasible where a list of all members of the relevant population exists. Where a study does not report the proportion of the source population from which the patients are derived, the question should be answered as unable to determine. |  |  | yes |  |
| *12. Were those subjects who were prepared to participate representative of the entire population from which they were recruited?*The proportion of those asked who agreed should be stated. Validation that the sample was representative would include demonstrating that the distribution of the main confounding factors was the same in the study sample and the source population. |  |  | yes |  |
| *13. Were the staff, places, and facilities where the patients were treated, representative of the treatment the majority of patients receive?* For the question to be answered yes the study should demonstrate that the intervention was representative of that in use in the source population. The question should be answered no if, for example, the intervention was undertaken in a specialist centre unrepresentative of the hospitals most of the source population would attend. | unable to determine |  |  |  |
| **Internal validity – bias** |  |  |  |  |
| *14. Was an attempt made to blind study subjects to the intervention they have received?* For studies where the patients would have no way of knowing which  intervention they received, this should be answered yes. |  | no |  |  |
| *15. Was an attempt made to blind those measuring the main outcomes of the intervention?* |  | no |  |  |
| *16. If any of the results of the study were based on “data dredging”, was this made clear?* Any analyses that had not been planned at the outset of the study should be clearly indicated. If no retrospective unplanned subgroup analyses were reported, then answer yes. |  |  | yes |  |
| *17. In trials and cohort studies, do the analyses adjust for different lengths of follow‐up of patients, or in case‐control studies, is the time period between the intervention and outcome the same for cases and controls?*Where follow‐up was the same for all study patients the answer should yes. If different lengths of follow‐up were adjusted for by, for example, survival analysis the answer should be yes. Studies where differences in follow‐up are ignored should be answered no. |  |  | yes |  |
| 1*8. Were the statistical tests used to assess the main outcomes appropriate?* The statistical techniques used must be appropriate to the data. For example nonparametric methods should be used for small sample sizes. Where little statistical analysis has been undertaken but where there is no evidence of bias, the question should be answered yes. If the distribution of the data (normal or not) is not described it must be assumed that the estimates used were appropriate and the question should be answered yes. |  |  | yes |  |
| *19. Was compliance with the intervention/s reliable*? Where there was non compliance with the allocated treatment or where there was contamination of one group, the question should be answered no. For studies where the effect of any misclassification was likely to bias any association to the null, the question should be answered yes |  |  | yes |  |
| *20. Were the main outcome measures used accurate (valid and reliable)?* For studies where the outcome measures are clearly described, the question should be answered yes. For studies which refer to other work or that demonstrates the outcome measures are accurate, the question should be answered as yes. |  |  | yes |  |
| **Internal validity ‐ confounding (selection bias)** |  |  |  |  |
| *21. Were the patients in different intervention groups (trials and cohort studies) or were the cases and controls (case‐control studies) recruited from the same population?* For example, patients for all comparison groups should be selected from the same hospital. The question should be answered unable to determine for cohort and casecontrol studies where there is no information concerning the source of patients included in the study. |  |  | yes |  |
| *22. Were study subjects in different intervention groups (trials and cohort studies) or were the cases and controls (case‐control studies) recruited over the same period of time?* For a study which does not specify the time period over which patients were recruited, the question should be answered as unable to determine. | unable to determine |  |  |  |
| *23. Were study subjects randomised to intervention groups?* Studies which state that subjects were randomized should be answered yes except where method of randomisation would not ensure random allocation. For example alternate allocation would score no because it is predictable. |  | no |  |  |
| *24. Was the randomised intervention assignment concealed from both patients and health care staff until recruitment was complete and irrevocable?* All non‐randomised studies should be answered no. If assignment was oncealed from patients but not from staff, it should be answered no. |  | no |  |  |
| *25. Was there adequate adjustment for confounding in the analyses from which the main findings were drawn?* This question should be answered no for trials if: the main conclusions of the study were based on analyses of treatment rather than intention to treat; the distribution of known confounders in the different treatment groups was not described; or the distribution of known confounders differed between the treatment groups but was not taken into account in the analyses. In nonrandomized studies if the effect of the main confounders was not investigated or confounding was demonstrated but no adjustment was made in the final analyses the question should be answered as no. |  | no |  |  |
| *26. Were losses of patients to follow‐up taken into account?* If the numbers of patients lost to follow‐up are not reported, the question should be answered as unable to determine. If the proportion lost to follow‐ up was too small to affect the main findings, the question should be answered yes. | unable to determine |  |  |  |
| **Power** |  |  |  |  |
| *27. Did the study have sufficient power to detect a clinically important effect where the probability value for a difference being due to chance is less than 5%?*Sample sizes have been calculated to detect a difference of x% and y%. |  | no |  | no a priori power analysis was conducted, unable to determine effect size of interested outcome |
| **Total score:** |  |  | 17 |  |
|  |  |  |  |  |
| **Article Author & Name: Said et al. 2019** | unable to determine | no | yes | **Notes/Justification** |
| **Reporting** | 0 | 0 | 1 |  |
| *1. Is the hypothesis/aim/objective of the study clearly described?* |  |  | yes |  |
| *2. Are the main outcomes to be measured clearly described in the Introduction or Methods section?* If the main outcomes are first mentioned in the Results section, the question should be answered no |  |  | yes |  |
| *3. Are the characteristics of the patients included in the study clearly described ?* In cohort studies and trials, inclusion and/or exclusion criteria should be given. In case‐control studies, a case‐definition and the source for controls should be given. |  |  | yes |  |
| *4. Are the interventions of interest clearly described?*Treatments and placebo (where relevant) that are to be compared should be clearly described. |  |  | yes |  |
| *5. Are the distributions of principal confounders in each group of subjects to be compared clearly described?* A list of principal confounders is provided. |  |  | yes (2) |  |
| *6. Are the main findings of the study clearly described?* Simple outcome data (including denominators and numerators) should be reported for all major findings so that the reader can check the major analyses and conclusions. (This question does not cover statistical tests which are considered below). |  |  | yes |  |
| *7. Does the study provide estimates of the random variability in the data for the main outcomes?* In non normally distributed data the inter‐quartile range of results should be reported. In normally distributed data the standard error, standard deviation or confidence intervals should be reported. If the distribution of the data is not described, it must be assumed that the estimates used were appropriate and the question should be answered yes. |  |  | yes |  |
| *8. Have all important adverse events that may be a consequence of the intervention been reported?* This should be answered yes if the study demonstrates that there was a comprehensive attempt to measure adverse events. (A list of possible adverse events is provided). |  | no |  |  |
| *9. Have the characteristics of patients lost to follow‐up been described?* This should be answered yes where there were no losses to follow‐up or where losses to follow‐up were so small that findings would be unaffected by their inclusion. This should be answered no where a study does not report the number of patients lost to follow‐up. |  | no |  |  |
| *10. Have actual probability values been reported ( e.g. 0.035 rather than <0.05) for the main outcomes except where the probability value is less than 0.001?* |  |  | yes |  |
| **External Validity** All the following criteria attempt to address the representativeness of the findings of the study and whether they may be generalised to the population from which the study subjects were derived. |  |  |  |  |
| *11. Were the subjects asked to participate in the study representative of the entire population from which they were recruited?* The study must identify the source population for patients and describe how the patients were selected. Patients would be representative if they comprised the entire source population, an unselected sample of consecutive patients, or a random sample. Random sampling is only feasible where a list of all members of the relevant population exists. Where a study does not report the proportion of the source population from which the patients are derived, the question should be answered as unable to determine. | unable to determine |  |  |  |
| *12. Were those subjects who were prepared to participate representative of the entire population from which they were recruited?*The proportion of those asked who agreed should be stated. Validation that the sample was representative would include demonstrating that the distribution of the main confounding factors was the same in the study sample and the source population. | unable to determine |  |  |  |
| *13. Were the staff, places, and facilities where the patients were treated, representative of the treatment the majority of patients receive?* For the question to be answered yes the study should demonstrate that the intervention was representative of that in use in the source population. The question should be answered no if, for example, the intervention was undertaken in a specialist centre unrepresentative of the hospitals most of the source population would attend. | unable to determine |  |  |  |
| **Internal validity – bias** |  |  |  |  |
| *14. Was an attempt made to blind study subjects to the intervention they have received?* For studies where the patients would have no way of knowing which  intervention they received, this should be answered yes. |  | no |  |  |
| *15. Was an attempt made to blind those measuring the main outcomes of the intervention?* |  | no |  |  |
| *16. If any of the results of the study were based on “data dredging”, was this made clear?* Any analyses that had not been planned at the outset of the study should be clearly indicated. If no retrospective unplanned subgroup analyses were reported, then answer yes. |  |  | yes |  |
| *17. In trials and cohort studies, do the analyses adjust for different lengths of follow‐up of patients, or in case‐control studies, is the time period between the intervention and outcome the same for cases and controls?*Where follow‐up was the same for all study patients the answer should yes. If different lengths of follow‐up were adjusted for by, for example, survival analysis the answer should be yes. Studies where differences in follow‐up are ignored should be answered no. |  |  | yes |  |
| 1*8. Were the statistical tests used to assess the main outcomes appropriate?* The statistical techniques used must be appropriate to the data. For example nonparametric methods should be used for small sample sizes. Where little statistical analysis has been undertaken but where there is no evidence of bias, the question should be answered yes. If the distribution of the data (normal or not) is not described it must be assumed that the estimates used were appropriate and the question should be answered yes. |  |  | yes |  |
| *19. Was compliance with the intervention/s reliable*? Where there was non compliance with the allocated treatment or where there was contamination of one group, the question should be answered no. For studies where the effect of any misclassification was likely to bias any association to the null, the question should be answered yes |  |  | yes |  |
| *20. Were the main outcome measures used accurate (valid and reliable)?* For studies where the outcome measures are clearly described, the question should be answered yes. For studies which refer to other work or that demonstrates the outcome measures are accurate, the question should be answered as yes. |  |  | yes |  |
| **Internal validity ‐ confounding (selection bias)** |  |  |  |  |
| *21. Were the patients in different intervention groups (trials and cohort studies) or were the cases and controls (case‐control studies) recruited from the same population?* For example, patients for all comparison groups should be selected from the same hospital. The question should be answered unable to determine for cohort and casecontrol studies where there is no information concerning the source of patients included in the study. | unable to determine |  |  |  |
| *22. Were study subjects in different intervention groups (trials and cohort studies) or were the cases and controls (case‐control studies) recruited over the same period of time?* For a study which does not specify the time period over which patients were recruited, the question should be answered as unable to determine. | unable to determine |  |  |  |
| *23. Were study subjects randomised to intervention groups?* Studies which state that subjects were randomized should be answered yes except where method of randomisation would not ensure random allocation. For example alternate allocation would score no because it is predictable. |  | no |  |  |
| *24. Was the randomised intervention assignment concealed from both patients and health care staff until recruitment was complete and irrevocable?* All non‐randomised studies should be answered no. If assignment was oncealed from patients but not from staff, it should be answered no. |  | no |  |  |
| *25. Was there adequate adjustment for confounding in the analyses from which the main findings were drawn?* This question should be answered no for trials if: the main conclusions of the study were based on analyses of treatment rather than intention to treat; the distribution of known confounders in the different treatment groups was not described; or the distribution of known confounders differed between the treatment groups but was not taken into account in the analyses. In nonrandomized studies if the effect of the main confounders was not investigated or confounding was demonstrated but no adjustment was made in the final analyses the question should be answered as no. |  | no |  |  |
| *26. Were losses of patients to follow‐up taken into account?* If the numbers of patients lost to follow‐up are not reported, the question should be answered as unable to determine. If the proportion lost to follow‐ up was too small to affect the main findings, the question should be answered yes. | unable to determine |  |  |  |
| **Power** |  |  |  |  |
| *27. Did the study have sufficient power to detect a clinically important effect where the probability value for a difference being due to chance is less than 5%?*Sample sizes have been calculated to detect a difference of x% and y%. |  |  | yes |  |
| **Total score:** |  |  | 15 |  |
|  |  |  |  |  |
| **Article Author & Name: Sauvet et al. 2010** | unable to determine | no | yes | **Notes/Justification** |
| **Reporting** | 0 | 0 | 1 |  |
| *1. Is the hypothesis/aim/objective of the study clearly described?* |  |  | yes |  |
| *2. Are the main outcomes to be measured clearly described in the Introduction or Methods section?* If the main outcomes are first mentioned in the Results section, the question should be answered no |  |  | yes |  |
| *3. Are the characteristics of the patients included in the study clearly described ?* In cohort studies and trials, inclusion and/or exclusion criteria should be given. In case‐control studies, a case‐definition and the source for controls should be given. |  |  | yes |  |
| *4. Are the interventions of interest clearly described?*Treatments and placebo (where relevant) that are to be compared should be clearly described. |  |  | yes |  |
| *5. Are the distributions of principal confounders in each group of subjects to be compared clearly described?* A list of principal confounders is provided. |  |  | yes (2) |  |
| *6. Are the main findings of the study clearly described?* Simple outcome data (including denominators and numerators) should be reported for all major findings so that the reader can check the major analyses and conclusions. (This question does not cover statistical tests which are considered below). |  |  | yes |  |
| *7. Does the study provide estimates of the random variability in the data for the main outcomes?* In non normally distributed data the inter‐quartile range of results should be reported. In normally distributed data the standard error, standard deviation or confidence intervals should be reported. If the distribution of the data is not described, it must be assumed that the estimates used were appropriate and the question should be answered yes. |  |  | yes |  |
| *8. Have all important adverse events that may be a consequence of the intervention been reported?* This should be answered yes if the study demonstrates that there was a comprehensive attempt to measure adverse events. (A list of possible adverse events is provided). |  | no |  |  |
| *9. Have the characteristics of patients lost to follow‐up been described?* This should be answered yes where there were no losses to follow‐up or where losses to follow‐up were so small that findings would be unaffected by their inclusion. This should be answered no where a study does not report the number of patients lost to follow‐up. |  |  | yes |  |
| *10. Have actual probability values been reported ( e.g. 0.035 rather than <0.05) for the main outcomes except where the probability value is less than 0.001?* |  |  | yes |  |
| **External Validity** All the following criteria attempt to address the representativeness of the findings of the study and whether they may be generalised to the population from which the study subjects were derived. |  |  |  |  |
| *11. Were the subjects asked to participate in the study representative of the entire population from which they were recruited?* The study must identify the source population for patients and describe how the patients were selected. Patients would be representative if they comprised the entire source population, an unselected sample of consecutive patients, or a random sample. Random sampling is only feasible where a list of all members of the relevant population exists. Where a study does not report the proportion of the source population from which the patients are derived, the question should be answered as unable to determine. | unable to determine |  |  |  |
| *12. Were those subjects who were prepared to participate representative of the entire population from which they were recruited?*The proportion of those asked who agreed should be stated. Validation that the sample was representative would include demonstrating that the distribution of the main confounding factors was the same in the study sample and the source population. | unable to determine |  |  |  |
| *13. Were the staff, places, and facilities where the patients were treated, representative of the treatment the majority of patients receive?* For the question to be answered yes the study should demonstrate that the intervention was representative of that in use in the source population. The question should be answered no if, for example, the intervention was undertaken in a specialist centre unrepresentative of the hospitals most of the source population would attend. | unable to determine |  |  |  |
| **Internal validity – bias** |  |  |  |  |
| *14. Was an attempt made to blind study subjects to the intervention they have received?* For studies where the patients would have no way of knowing which  intervention they received, this should be answered yes. |  | no |  |  |
| *15. Was an attempt made to blind those measuring the main outcomes of the intervention?* |  | no |  |  |
| *16. If any of the results of the study were based on “data dredging”, was this made clear?* Any analyses that had not been planned at the outset of the study should be clearly indicated. If no retrospective unplanned subgroup analyses were reported, then answer yes. |  |  | yes |  |
| *17. In trials and cohort studies, do the analyses adjust for different lengths of follow‐up of patients, or in case‐control studies, is the time period between the intervention and outcome the same for cases and controls?*Where follow‐up was the same for all study patients the answer should yes. If different lengths of follow‐up were adjusted for by, for example, survival analysis the answer should be yes. Studies where differences in follow‐up are ignored should be answered no. |  |  | yes |  |
| 1*8. Were the statistical tests used to assess the main outcomes appropriate?* The statistical techniques used must be appropriate to the data. For example nonparametric methods should be used for small sample sizes. Where little statistical analysis has been undertaken but where there is no evidence of bias, the question should be answered yes. If the distribution of the data (normal or not) is not described it must be assumed that the estimates used were appropriate and the question should be answered yes. |  |  | yes |  |
| *19. Was compliance with the intervention/s reliable*? Where there was non compliance with the allocated treatment or where there was contamination of one group, the question should be answered no. For studies where the effect of any misclassification was likely to bias any association to the null, the question should be answered yes |  |  | yes |  |
| *20. Were the main outcome measures used accurate (valid and reliable)?* For studies where the outcome measures are clearly described, the question should be answered yes. For studies which refer to other work or that demonstrates the outcome measures are accurate, the question should be answered as yes. |  |  | yes |  |
| **Internal validity ‐ confounding (selection bias)** |  |  |  |  |
| *21. Were the patients in different intervention groups (trials and cohort studies) or were the cases and controls (case‐control studies) recruited from the same population?* For example, patients for all comparison groups should be selected from the same hospital. The question should be answered unable to determine for cohort and casecontrol studies where there is no information concerning the source of patients included in the study. | unable to determine |  |  |  |
| *22. Were study subjects in different intervention groups (trials and cohort studies) or were the cases and controls (case‐control studies) recruited over the same period of time?* For a study which does not specify the time period over which patients were recruited, the question should be answered as unable to determine. | unable to determine |  |  |  |
| *23. Were study subjects randomised to intervention groups?* Studies which state that subjects were randomized should be answered yes except where method of randomisation would not ensure random allocation. For example alternate allocation would score no because it is predictable. |  | no |  |  |
| *24. Was the randomised intervention assignment concealed from both patients and health care staff until recruitment was complete and irrevocable?* All non‐randomised studies should be answered no. If assignment was oncealed from patients but not from staff, it should be answered no. |  | no |  |  |
| *25. Was there adequate adjustment for confounding in the analyses from which the main findings were drawn?* This question should be answered no for trials if: the main conclusions of the study were based on analyses of treatment rather than intention to treat; the distribution of known confounders in the different treatment groups was not described; or the distribution of known confounders differed between the treatment groups but was not taken into account in the analyses. In nonrandomized studies if the effect of the main confounders was not investigated or confounding was demonstrated but no adjustment was made in the final analyses the question should be answered as no. |  | no |  |  |
| *26. Were losses of patients to follow‐up taken into account?* If the numbers of patients lost to follow‐up are not reported, the question should be answered as unable to determine. If the proportion lost to follow‐ up was too small to affect the main findings, the question should be answered yes. |  |  | yes |  |
| **Power** |  |  |  |  |
| *27. Did the study have sufficient power to detect a clinically important effect where the probability value for a difference being due to chance is less than 5%?*Sample sizes have been calculated to detect a difference of x% and y%. |  | no |  | no a priori power analysis was conducted, unable to determine effect size of interested outcome |
| **Total score:** |  |  | 16 |  |
|  |  |  |  |  |
| **Article Author & Name: Sauvet et al. 2015** | unable to determine | no | yes | **Notes/Justification** |
| **Reporting** | 0 | 0 | 1 |  |
| *1. Is the hypothesis/aim/objective of the study clearly described?* |  |  | yes |  |
| *2. Are the main outcomes to be measured clearly described in the Introduction or Methods section?* If the main outcomes are first mentioned in the Results section, the question should be answered no |  |  | yes |  |
| *3. Are the characteristics of the patients included in the study clearly described ?* In cohort studies and trials, inclusion and/or exclusion criteria should be given. In case‐control studies, a case‐definition and the source for controls should be given. |  |  | yes |  |
| *4. Are the interventions of interest clearly described?*Treatments and placebo (where relevant) that are to be compared should be clearly described. |  |  | yes |  |
| *5. Are the distributions of principal confounders in each group of subjects to be compared clearly described?* A list of principal confounders is provided. |  |  | yes (2) |  |
| *6. Are the main findings of the study clearly described?* Simple outcome data (including denominators and numerators) should be reported for all major findings so that the reader can check the major analyses and conclusions. (This question does not cover statistical tests which are considered below). |  |  | yes |  |
| *7. Does the study provide estimates of the random variability in the data for the main outcomes?* In non normally distributed data the inter‐quartile range of results should be reported. In normally distributed data the standard error, standard deviation or confidence intervals should be reported. If the distribution of the data is not described, it must be assumed that the estimates used were appropriate and the question should be answered yes. |  |  | yes |  |
| *8. Have all important adverse events that may be a consequence of the intervention been reported?* This should be answered yes if the study demonstrates that there was a comprehensive attempt to measure adverse events. (A list of possible adverse events is provided). |  | no |  |  |
| *9. Have the characteristics of patients lost to follow‐up been described?* This should be answered yes where there were no losses to follow‐up or where losses to follow‐up were so small that findings would be unaffected by their inclusion. This should be answered no where a study does not report the number of patients lost to follow‐up. |  | no |  |  |
| *10. Have actual probability values been reported ( e.g. 0.035 rather than <0.05) for the main outcomes except where the probability value is less than 0.001?* |  | no |  |  |
| **External Validity** All the following criteria attempt to address the representativeness of the findings of the study and whether they may be generalised to the population from which the study subjects were derived. |  |  |  |  |
| *11. Were the subjects asked to participate in the study representative of the entire population from which they were recruited?* The study must identify the source population for patients and describe how the patients were selected. Patients would be representative if they comprised the entire source population, an unselected sample of consecutive patients, or a random sample. Random sampling is only feasible where a list of all members of the relevant population exists. Where a study does not report the proportion of the source population from which the patients are derived, the question should be answered as unable to determine. | unable to determine |  |  |  |
| *12. Were those subjects who were prepared to participate representative of the entire population from which they were recruited?*The proportion of those asked who agreed should be stated. Validation that the sample was representative would include demonstrating that the distribution of the main confounding factors was the same in the study sample and the source population. | unable to determine |  |  |  |
| *13. Were the staff, places, and facilities where the patients were treated, representative of the treatment the majority of patients receive?* For the question to be answered yes the study should demonstrate that the intervention was representative of that in use in the source population. The question should be answered no if, for example, the intervention was undertaken in a specialist centre unrepresentative of the hospitals most of the source population would attend. | unable to determine |  |  |  |
| **Internal validity – bias** |  |  |  |  |
| *14. Was an attempt made to blind study subjects to the intervention they have received?* For studies where the patients would have no way of knowing which  intervention they received, this should be answered yes. |  | no |  |  |
| *15. Was an attempt made to blind those measuring the main outcomes of the intervention?* |  | no |  |  |
| *16. If any of the results of the study were based on “data dredging”, was this made clear?* Any analyses that had not been planned at the outset of the study should be clearly indicated. If no retrospective unplanned subgroup analyses were reported, then answer yes. |  |  | yes |  |
| *17. In trials and cohort studies, do the analyses adjust for different lengths of follow‐up of patients, or in case‐control studies, is the time period between the intervention and outcome the same for cases and controls?*Where follow‐up was the same for all study patients the answer should yes. If different lengths of follow‐up were adjusted for by, for example, survival analysis the answer should be yes. Studies where differences in follow‐up are ignored should be answered no. |  |  | yes |  |
| 1*8. Were the statistical tests used to assess the main outcomes appropriate?* The statistical techniques used must be appropriate to the data. For example nonparametric methods should be used for small sample sizes. Where little statistical analysis has been undertaken but where there is no evidence of bias, the question should be answered yes. If the distribution of the data (normal or not) is not described it must be assumed that the estimates used were appropriate and the question should be answered yes. |  |  | yes |  |
| *19. Was compliance with the intervention/s reliable*? Where there was non compliance with the allocated treatment or where there was contamination of one group, the question should be answered no. For studies where the effect of any misclassification was likely to bias any association to the null, the question should be answered yes |  |  | yes |  |
| *20. Were the main outcome measures used accurate (valid and reliable)?* For studies where the outcome measures are clearly described, the question should be answered yes. For studies which refer to other work or that demonstrates the outcome measures are accurate, the question should be answered as yes. |  |  | yes |  |
| **Internal validity ‐ confounding (selection bias)** |  |  |  |  |
| *21. Were the patients in different intervention groups (trials and cohort studies) or were the cases and controls (case‐control studies) recruited from the same population?* For example, patients for all comparison groups should be selected from the same hospital. The question should be answered unable to determine for cohort and casecontrol studies where there is no information concerning the source of patients included in the study. | unable to determine |  |  |  |
| *22. Were study subjects in different intervention groups (trials and cohort studies) or were the cases and controls (case‐control studies) recruited over the same period of time?* For a study which does not specify the time period over which patients were recruited, the question should be answered as unable to determine. | unable to determine |  |  |  |
| *23. Were study subjects randomised to intervention groups?* Studies which state that subjects were randomized should be answered yes except where method of randomisation would not ensure random allocation. For example alternate allocation would score no because it is predictable. |  | no |  |  |
| *24. Was the randomised intervention assignment concealed from both patients and health care staff until recruitment was complete and irrevocable?* All non‐randomised studies should be answered no. If assignment was oncealed from patients but not from staff, it should be answered no. |  | no |  |  |
| *25. Was there adequate adjustment for confounding in the analyses from which the main findings were drawn?* This question should be answered no for trials if: the main conclusions of the study were based on analyses of treatment rather than intention to treat; the distribution of known confounders in the different treatment groups was not described; or the distribution of known confounders differed between the treatment groups but was not taken into account in the analyses. In nonrandomized studies if the effect of the main confounders was not investigated or confounding was demonstrated but no adjustment was made in the final analyses the question should be answered as no. |  | no |  |  |
| *26. Were losses of patients to follow‐up taken into account?* If the numbers of patients lost to follow‐up are not reported, the question should be answered as unable to determine. If the proportion lost to follow‐ up was too small to affect the main findings, the question should be answered yes. | unable to determine |  |  |  |
| **Power** |  |  |  |  |
| *27. Did the study have sufficient power to detect a clinically important effect where the probability value for a difference being due to chance is less than 5%?*Sample sizes have been calculated to detect a difference of x% and y%. |  | no |  |  |
| **Total score:** |  |  | 13 |  |
|  |  |  |  |  |
| **Article Author & Name: Schmid et al. 2011** | unable to determine | no | yes | **Notes/Justification** |
| **Reporting** | 0 | 0 | 1 |  |
| *1. Is the hypothesis/aim/objective of the study clearly described?* |  |  | yes |  |
| *2. Are the main outcomes to be measured clearly described in the Introduction or Methods section?* If the main outcomes are first mentioned in the Results section, the question should be answered no |  |  | yes |  |
| *3. Are the characteristics of the patients included in the study clearly described ?* In cohort studies and trials, inclusion and/or exclusion criteria should be given. In case‐control studies, a case‐definition and the source for controls should be given. |  |  | yes |  |
| *4. Are the interventions of interest clearly described?*Treatments and placebo (where relevant) that are to be compared should be clearly described. |  |  | yes |  |
| *5. Are the distributions of principal confounders in each group of subjects to be compared clearly described?* A list of principal confounders is provided. |  |  | yes (2) |  |
| *6. Are the main findings of the study clearly described?* Simple outcome data (including denominators and numerators) should be reported for all major findings so that the reader can check the major analyses and conclusions. (This question does not cover statistical tests which are considered below). |  |  | yes |  |
| *7. Does the study provide estimates of the random variability in the data for the main outcomes?* In non normally distributed data the inter‐quartile range of results should be reported. In normally distributed data the standard error, standard deviation or confidence intervals should be reported. If the distribution of the data is not described, it must be assumed that the estimates used were appropriate and the question should be answered yes. |  |  | yes |  |
| *8. Have all important adverse events that may be a consequence of the intervention been reported?* This should be answered yes if the study demonstrates that there was a comprehensive attempt to measure adverse events. (A list of possible adverse events is provided). |  | no |  |  |
| *9. Have the characteristics of patients lost to follow‐up been described?* This should be answered yes where there were no losses to follow‐up or where losses to follow‐up were so small that findings would be unaffected by their inclusion. This should be answered no where a study does not report the number of patients lost to follow‐up. |  | no |  |  |
| *10. Have actual probability values been reported ( e.g. 0.035 rather than <0.05) for the main outcomes except where the probability value is less than 0.001?* |  |  | yes |  |
| **External Validity** All the following criteria attempt to address the representativeness of the findings of the study and whether they may be generalised to the population from which the study subjects were derived. |  |  |  |  |
| *11. Were the subjects asked to participate in the study representative of the entire population from which they were recruited?* The study must identify the source population for patients and describe how the patients were selected. Patients would be representative if they comprised the entire source population, an unselected sample of consecutive patients, or a random sample. Random sampling is only feasible where a list of all members of the relevant population exists. Where a study does not report the proportion of the source population from which the patients are derived, the question should be answered as unable to determine. | unable to determine |  |  |  |
| *12. Were those subjects who were prepared to participate representative of the entire population from which they were recruited?*The proportion of those asked who agreed should be stated. Validation that the sample was representative would include demonstrating that the distribution of the main confounding factors was the same in the study sample and the source population. | unable to determine |  |  |  |
| *13. Were the staff, places, and facilities where the patients were treated, representative of the treatment the majority of patients receive?* For the question to be answered yes the study should demonstrate that the intervention was representative of that in use in the source population. The question should be answered no if, for example, the intervention was undertaken in a specialist centre unrepresentative of the hospitals most of the source population would attend. | unable to determine |  |  |  |
| **Internal validity – bias** |  |  |  |  |
| *14. Was an attempt made to blind study subjects to the intervention they have received?* For studies where the patients would have no way of knowing which  intervention they received, this should be answered yes. |  | no |  |  |
| *15. Was an attempt made to blind those measuring the main outcomes of the intervention?* |  | no |  |  |
| *16. If any of the results of the study were based on “data dredging”, was this made clear?* Any analyses that had not been planned at the outset of the study should be clearly indicated. If no retrospective unplanned subgroup analyses were reported, then answer yes. |  |  | yes |  |
| *17. In trials and cohort studies, do the analyses adjust for different lengths of follow‐up of patients, or in case‐control studies, is the time period between the intervention and outcome the same for cases and controls?*Where follow‐up was the same for all study patients the answer should yes. If different lengths of follow‐up were adjusted for by, for example, survival analysis the answer should be yes. Studies where differences in follow‐up are ignored should be answered no. |  |  | yes |  |
| 1*8. Were the statistical tests used to assess the main outcomes appropriate?* The statistical techniques used must be appropriate to the data. For example nonparametric methods should be used for small sample sizes. Where little statistical analysis has been undertaken but where there is no evidence of bias, the question should be answered yes. If the distribution of the data (normal or not) is not described it must be assumed that the estimates used were appropriate and the question should be answered yes. |  |  | yes |  |
| *19. Was compliance with the intervention/s reliable*? Where there was non compliance with the allocated treatment or where there was contamination of one group, the question should be answered no. For studies where the effect of any misclassification was likely to bias any association to the null, the question should be answered yes |  |  | yes |  |
| *20. Were the main outcome measures used accurate (valid and reliable)?* For studies where the outcome measures are clearly described, the question should be answered yes. For studies which refer to other work or that demonstrates the outcome measures are accurate, the question should be answered as yes. |  |  | yes |  |
| **Internal validity ‐ confounding (selection bias)** |  |  |  |  |
| *21. Were the patients in different intervention groups (trials and cohort studies) or were the cases and controls (case‐control studies) recruited from the same population?* For example, patients for all comparison groups should be selected from the same hospital. The question should be answered unable to determine for cohort and casecontrol studies where there is no information concerning the source of patients included in the study. | unable to determine |  |  |  |
| *22. Were study subjects in different intervention groups (trials and cohort studies) or were the cases and controls (case‐control studies) recruited over the same period of time?* For a study which does not specify the time period over which patients were recruited, the question should be answered as unable to determine. | unable to determine |  |  |  |
| *23. Were study subjects randomised to intervention groups?* Studies which state that subjects were randomized should be answered yes except where method of randomisation would not ensure random allocation. For example alternate allocation would score no because it is predictable. |  | no |  |  |
| *24. Was the randomised intervention assignment concealed from both patients and health care staff until recruitment was complete and irrevocable?* All non‐randomised studies should be answered no. If assignment was oncealed from patients but not from staff, it should be answered no. |  | no |  |  |
| *25. Was there adequate adjustment for confounding in the analyses from which the main findings were drawn?* This question should be answered no for trials if: the main conclusions of the study were based on analyses of treatment rather than intention to treat; the distribution of known confounders in the different treatment groups was not described; or the distribution of known confounders differed between the treatment groups but was not taken into account in the analyses. In nonrandomized studies if the effect of the main confounders was not investigated or confounding was demonstrated but no adjustment was made in the final analyses the question should be answered as no. |  | no |  |  |
| *26. Were losses of patients to follow‐up taken into account?* If the numbers of patients lost to follow‐up are not reported, the question should be answered as unable to determine. If the proportion lost to follow‐ up was too small to affect the main findings, the question should be answered yes. | unable to determine |  |  |  |
| **Power** |  |  |  |  |
| *27. Did the study have sufficient power to detect a clinically important effect where the probability value for a difference being due to chance is less than 5%?*Sample sizes have been calculated to detect a difference of x% and y%. |  |  | yes |  |
| **Total score:** |  |  | 15 |  |
|  |  |  |  |  |
| **Article Author & Name: Simpson et al. 2016** | unable to determine | no | yes | **Notes/Justification** |
| **Reporting** | 0 | 0 | 1 |  |
| *1. Is the hypothesis/aim/objective of the study clearly described?* |  |  | yes |  |
| *2. Are the main outcomes to be measured clearly described in the Introduction or Methods section?* If the main outcomes are first mentioned in the Results section, the question should be answered no |  |  | yes |  |
| *3. Are the characteristics of the patients included in the study clearly described ?* In cohort studies and trials, inclusion and/or exclusion criteria should be given. In case‐control studies, a case‐definition and the source for controls should be given. |  |  | yes |  |
| *4. Are the interventions of interest clearly described?*Treatments and placebo (where relevant) that are to be compared should be clearly described. |  |  | yes |  |
| *5. Are the distributions of principal confounders in each group of subjects to be compared clearly described?* A list of principal confounders is provided. |  |  | yes (2) |  |
| *6. Are the main findings of the study clearly described?* Simple outcome data (including denominators and numerators) should be reported for all major findings so that the reader can check the major analyses and conclusions. (This question does not cover statistical tests which are considered below). |  |  | yes |  |
| *7. Does the study provide estimates of the random variability in the data for the main outcomes?* In non normally distributed data the inter‐quartile range of results should be reported. In normally distributed data the standard error, standard deviation or confidence intervals should be reported. If the distribution of the data is not described, it must be assumed that the estimates used were appropriate and the question should be answered yes. |  |  | yes |  |
| *8. Have all important adverse events that may be a consequence of the intervention been reported?* This should be answered yes if the study demonstrates that there was a comprehensive attempt to measure adverse events. (A list of possible adverse events is provided). |  | no |  |  |
| *9. Have the characteristics of patients lost to follow‐up been described?* This should be answered yes where there were no losses to follow‐up or where losses to follow‐up were so small that findings would be unaffected by their inclusion. This should be answered no where a study does not report the number of patients lost to follow‐up. |  |  | yes |  |
| *10. Have actual probability values been reported ( e.g. 0.035 rather than <0.05) for the main outcomes except where the probability value is less than 0.001?* |  | no |  |  |
| **External Validity** All the following criteria attempt to address the representativeness of the findings of the study and whether they may be generalised to the population from which the study subjects were derived. |  |  |  |  |
| *11. Were the subjects asked to participate in the study representative of the entire population from which they were recruited?* The study must identify the source population for patients and describe how the patients were selected. Patients would be representative if they comprised the entire source population, an unselected sample of consecutive patients, or a random sample. Random sampling is only feasible where a list of all members of the relevant population exists. Where a study does not report the proportion of the source population from which the patients are derived, the question should be answered as unable to determine. | unable to determine |  |  |  |
| *12. Were those subjects who were prepared to participate representative of the entire population from which they were recruited?*The proportion of those asked who agreed should be stated. Validation that the sample was representative would include demonstrating that the distribution of the main confounding factors was the same in the study sample and the source population. | unable to determine |  |  |  |
| *13. Were the staff, places, and facilities where the patients were treated, representative of the treatment the majority of patients receive?* For the question to be answered yes the study should demonstrate that the intervention was representative of that in use in the source population. The question should be answered no if, for example, the intervention was undertaken in a specialist centre unrepresentative of the hospitals most of the source population would attend. | unable to determine |  |  |  |
| **Internal validity – bias** |  |  |  |  |
| *14. Was an attempt made to blind study subjects to the intervention they have received?* For studies where the patients would have no way of knowing which  intervention they received, this should be answered yes. |  | no |  |  |
| *15. Was an attempt made to blind those measuring the main outcomes of the intervention?* |  | no |  |  |
| *16. If any of the results of the study were based on “data dredging”, was this made clear?* Any analyses that had not been planned at the outset of the study should be clearly indicated. If no retrospective unplanned subgroup analyses were reported, then answer yes. |  |  | yes |  |
| *17. In trials and cohort studies, do the analyses adjust for different lengths of follow‐up of patients, or in case‐control studies, is the time period between the intervention and outcome the same for cases and controls?*Where follow‐up was the same for all study patients the answer should yes. If different lengths of follow‐up were adjusted for by, for example, survival analysis the answer should be yes. Studies where differences in follow‐up are ignored should be answered no. |  |  | yes |  |
| 1*8. Were the statistical tests used to assess the main outcomes appropriate?* The statistical techniques used must be appropriate to the data. For example nonparametric methods should be used for small sample sizes. Where little statistical analysis has been undertaken but where there is no evidence of bias, the question should be answered yes. If the distribution of the data (normal or not) is not described it must be assumed that the estimates used were appropriate and the question should be answered yes. |  |  | yes |  |
| *19. Was compliance with the intervention/s reliable*? Where there was non compliance with the allocated treatment or where there was contamination of one group, the question should be answered no. For studies where the effect of any misclassification was likely to bias any association to the null, the question should be answered yes |  |  | yes |  |
| *20. Were the main outcome measures used accurate (valid and reliable)?* For studies where the outcome measures are clearly described, the question should be answered yes. For studies which refer to other work or that demonstrates the outcome measures are accurate, the question should be answered as yes. |  |  | yes |  |
| **Internal validity ‐ confounding (selection bias)** |  |  |  |  |
| *21. Were the patients in different intervention groups (trials and cohort studies) or were the cases and controls (case‐control studies) recruited from the same population?* For example, patients for all comparison groups should be selected from the same hospital. The question should be answered unable to determine for cohort and casecontrol studies where there is no information concerning the source of patients included in the study. | unable to determine |  |  |  |
| *22. Were study subjects in different intervention groups (trials and cohort studies) or were the cases and controls (case‐control studies) recruited over the same period of time?* For a study which does not specify the time period over which patients were recruited, the question should be answered as unable to determine. | unable to determine |  |  |  |
| *23. Were study subjects randomised to intervention groups?* Studies which state that subjects were randomized should be answered yes except where method of randomisation would not ensure random allocation. For example alternate allocation would score no because it is predictable. |  |  | yes |  |
| *24. Was the randomised intervention assignment concealed from both patients and health care staff until recruitment was complete and irrevocable?* All non‐randomised studies should be answered no. If assignment was oncealed from patients but not from staff, it should be answered no. | unable to determine |  |  |  |
| *25. Was there adequate adjustment for confounding in the analyses from which the main findings were drawn?* This question should be answered no for trials if: the main conclusions of the study were based on analyses of treatment rather than intention to treat; the distribution of known confounders in the different treatment groups was not described; or the distribution of known confounders differed between the treatment groups but was not taken into account in the analyses. In nonrandomized studies if the effect of the main confounders was not investigated or confounding was demonstrated but no adjustment was made in the final analyses the question should be answered as no. |  | no |  |  |
| *26. Were losses of patients to follow‐up taken into account?* If the numbers of patients lost to follow‐up are not reported, the question should be answered as unable to determine. If the proportion lost to follow‐ up was too small to affect the main findings, the question should be answered yes. |  |  | yes |  |
| **Power** |  |  |  |  |
| *27. Did the study have sufficient power to detect a clinically important effect where the probability value for a difference being due to chance is less than 5%?*Sample sizes have been calculated to detect a difference of x% and y%. |  | no |  | no a priori power analysis was conducted, unable to determine effect size of interested outcome |
| **Total score:** |  |  | 16 |  |
|  |  |  |  |  |
| **Article Author & Name: Thompson et al. 2022** | unable to determine | no | yes | **Notes/Justification** |
| **Reporting** | 0 | 0 | 1 |  |
| *1. Is the hypothesis/aim/objective of the study clearly described?* |  |  | yes |  |
| *2. Are the main outcomes to be measured clearly described in the Introduction or Methods section?* If the main outcomes are first mentioned in the Results section, the question should be answered no |  |  | yes |  |
| *3. Are the characteristics of the patients included in the study clearly described ?* In cohort studies and trials, inclusion and/or exclusion criteria should be given. In case‐control studies, a case‐definition and the source for controls should be given. |  |  | yes |  |
| *4. Are the interventions of interest clearly described?*Treatments and placebo (where relevant) that are to be compared should be clearly described. |  |  | yes |  |
| *5. Are the distributions of principal confounders in each group of subjects to be compared clearly described?* A list of principal confounders is provided. |  |  | yes (2) |  |
| *6. Are the main findings of the study clearly described?* Simple outcome data (including denominators and numerators) should be reported for all major findings so that the reader can check the major analyses and conclusions. (This question does not cover statistical tests which are considered below). |  |  | yes |  |
| *7. Does the study provide estimates of the random variability in the data for the main outcomes?* In non normally distributed data the inter‐quartile range of results should be reported. In normally distributed data the standard error, standard deviation or confidence intervals should be reported. If the distribution of the data is not described, it must be assumed that the estimates used were appropriate and the question should be answered yes. |  |  | yes |  |
| *8. Have all important adverse events that may be a consequence of the intervention been reported?* This should be answered yes if the study demonstrates that there was a comprehensive attempt to measure adverse events. (A list of possible adverse events is provided). |  | no |  |  |
| *9. Have the characteristics of patients lost to follow‐up been described?* This should be answered yes where there were no losses to follow‐up or where losses to follow‐up were so small that findings would be unaffected by their inclusion. This should be answered no where a study does not report the number of patients lost to follow‐up. |  | no |  |  |
| *10. Have actual probability values been reported ( e.g. 0.035 rather than <0.05) for the main outcomes except where the probability value is less than 0.001?* |  |  | yes |  |
| **External Validity** All the following criteria attempt to address the representativeness of the findings of the study and whether they may be generalised to the population from which the study subjects were derived. |  |  |  |  |
| *11. Were the subjects asked to participate in the study representative of the entire population from which they were recruited?* The study must identify the source population for patients and describe how the patients were selected. Patients would be representative if they comprised the entire source population, an unselected sample of consecutive patients, or a random sample. Random sampling is only feasible where a list of all members of the relevant population exists. Where a study does not report the proportion of the source population from which the patients are derived, the question should be answered as unable to determine. | unable to determine |  |  |  |
| *12. Were those subjects who were prepared to participate representative of the entire population from which they were recruited?*The proportion of those asked who agreed should be stated. Validation that the sample was representative would include demonstrating that the distribution of the main confounding factors was the same in the study sample and the source population. | unable to determine |  |  |  |
| *13. Were the staff, places, and facilities where the patients were treated, representative of the treatment the majority of patients receive?* For the question to be answered yes the study should demonstrate that the intervention was representative of that in use in the source population. The question should be answered no if, for example, the intervention was undertaken in a specialist centre unrepresentative of the hospitals most of the source population would attend. | unable to determine |  |  |  |
| **Internal validity – bias** |  |  |  |  |
| *14. Was an attempt made to blind study subjects to the intervention they have received?* For studies where the patients would have no way of knowing which  intervention they received, this should be answered yes. |  | no |  |  |
| *15. Was an attempt made to blind those measuring the main outcomes of the intervention?* |  | no |  |  |
| *16. If any of the results of the study were based on “data dredging”, was this made clear?* Any analyses that had not been planned at the outset of the study should be clearly indicated. If no retrospective unplanned subgroup analyses were reported, then answer yes. |  |  | yes |  |
| *17. In trials and cohort studies, do the analyses adjust for different lengths of follow‐up of patients, or in case‐control studies, is the time period between the intervention and outcome the same for cases and controls?*Where follow‐up was the same for all study patients the answer should yes. If different lengths of follow‐up were adjusted for by, for example, survival analysis the answer should be yes. Studies where differences in follow‐up are ignored should be answered no. |  |  | yes |  |
| 1*8. Were the statistical tests used to assess the main outcomes appropriate?* The statistical techniques used must be appropriate to the data. For example nonparametric methods should be used for small sample sizes. Where little statistical analysis has been undertaken but where there is no evidence of bias, the question should be answered yes. If the distribution of the data (normal or not) is not described it must be assumed that the estimates used were appropriate and the question should be answered yes. |  |  | yes |  |
| *19. Was compliance with the intervention/s reliable*? Where there was non compliance with the allocated treatment or where there was contamination of one group, the question should be answered no. For studies where the effect of any misclassification was likely to bias any association to the null, the question should be answered yes |  |  | yes |  |
| *20. Were the main outcome measures used accurate (valid and reliable)?* For studies where the outcome measures are clearly described, the question should be answered yes. For studies which refer to other work or that demonstrates the outcome measures are accurate, the question should be answered as yes. |  |  | yes |  |
| **Internal validity ‐ confounding (selection bias)** |  |  |  |  |
| *21. Were the patients in different intervention groups (trials and cohort studies) or were the cases and controls (case‐control studies) recruited from the same population?* For example, patients for all comparison groups should be selected from the same hospital. The question should be answered unable to determine for cohort and casecontrol studies where there is no information concerning the source of patients included in the study. | unable to determine |  |  |  |
| *22. Were study subjects in different intervention groups (trials and cohort studies) or were the cases and controls (case‐control studies) recruited over the same period of time?* For a study which does not specify the time period over which patients were recruited, the question should be answered as unable to determine. | unable to determine |  |  |  |
| *23. Were study subjects randomised to intervention groups?* Studies which state that subjects were randomized should be answered yes except where method of randomisation would not ensure random allocation. For example alternate allocation would score no because it is predictable. |  | no |  |  |
| *24. Was the randomised intervention assignment concealed from both patients and health care staff until recruitment was complete and irrevocable?* All non‐randomised studies should be answered no. If assignment was oncealed from patients but not from staff, it should be answered no. |  | no |  |  |
| *25. Was there adequate adjustment for confounding in the analyses from which the main findings were drawn?* This question should be answered no for trials if: the main conclusions of the study were based on analyses of treatment rather than intention to treat; the distribution of known confounders in the different treatment groups was not described; or the distribution of known confounders differed between the treatment groups but was not taken into account in the analyses. In nonrandomized studies if the effect of the main confounders was not investigated or confounding was demonstrated but no adjustment was made in the final analyses the question should be answered as no. |  | no |  |  |
| *26. Were losses of patients to follow‐up taken into account?* If the numbers of patients lost to follow‐up are not reported, the question should be answered as unable to determine. If the proportion lost to follow‐ up was too small to affect the main findings, the question should be answered yes. | unable to determine |  |  |  |
| **Power** |  |  |  |  |
| *27. Did the study have sufficient power to detect a clinically important effect where the probability value for a difference being due to chance is less than 5%?*Sample sizes have been calculated to detect a difference of x% and y%. |  |  | yes | no a priori power analysis was conducted, adequate effect size |
| **Total score:** |  |  | 15 |  |
|  |  |  |  |  |
| **Article Author & Name: van Leeuwen et al. 2009** | unable to determine | no | yes | **Notes/Justification** |
| **Reporting** | 0 | 0 | 1 |  |
| *1. Is the hypothesis/aim/objective of the study clearly described?* |  |  | yes |  |
| *2. Are the main outcomes to be measured clearly described in the Introduction or Methods section?* If the main outcomes are first mentioned in the Results section, the question should be answered no |  |  | yes |  |
| *3. Are the characteristics of the patients included in the study clearly described ?* In cohort studies and trials, inclusion and/or exclusion criteria should be given. In case‐control studies, a case‐definition and the source for controls should be given. |  |  | yes |  |
| *4. Are the interventions of interest clearly described?*Treatments and placebo (where relevant) that are to be compared should be clearly described. |  |  | yes |  |
| *5. Are the distributions of principal confounders in each group of subjects to be compared clearly described?* A list of principal confounders is provided. |  |  | yes (2) |  |
| *6. Are the main findings of the study clearly described?* Simple outcome data (including denominators and numerators) should be reported for all major findings so that the reader can check the major analyses and conclusions. (This question does not cover statistical tests which are considered below). |  |  | yes |  |
| *7. Does the study provide estimates of the random variability in the data for the main outcomes?* In non normally distributed data the inter‐quartile range of results should be reported. In normally distributed data the standard error, standard deviation or confidence intervals should be reported. If the distribution of the data is not described, it must be assumed that the estimates used were appropriate and the question should be answered yes. |  |  | yes |  |
| *8. Have all important adverse events that may be a consequence of the intervention been reported?* This should be answered yes if the study demonstrates that there was a comprehensive attempt to measure adverse events. (A list of possible adverse events is provided). |  | no |  |  |
| *9. Have the characteristics of patients lost to follow‐up been described?* This should be answered yes where there were no losses to follow‐up or where losses to follow‐up were so small that findings would be unaffected by their inclusion. This should be answered no where a study does not report the number of patients lost to follow‐up. |  | no |  |  |
| *10. Have actual probability values been reported ( e.g. 0.035 rather than <0.05) for the main outcomes except where the probability value is less than 0.001?* |  |  | yes |  |
| **External Validity** All the following criteria attempt to address the representativeness of the findings of the study and whether they may be generalised to the population from which the study subjects were derived. |  |  |  |  |
| *11. Were the subjects asked to participate in the study representative of the entire population from which they were recruited?* The study must identify the source population for patients and describe how the patients were selected. Patients would be representative if they comprised the entire source population, an unselected sample of consecutive patients, or a random sample. Random sampling is only feasible where a list of all members of the relevant population exists. Where a study does not report the proportion of the source population from which the patients are derived, the question should be answered as unable to determine. | unable to determine |  |  |  |
| *12. Were those subjects who were prepared to participate representative of the entire population from which they were recruited?*The proportion of those asked who agreed should be stated. Validation that the sample was representative would include demonstrating that the distribution of the main confounding factors was the same in the study sample and the source population. | unable to determine |  |  |  |
| *13. Were the staff, places, and facilities where the patients were treated, representative of the treatment the majority of patients receive?* For the question to be answered yes the study should demonstrate that the intervention was representative of that in use in the source population. The question should be answered no if, for example, the intervention was undertaken in a specialist centre unrepresentative of the hospitals most of the source population would attend. |  |  | yes |  |
| **Internal validity – bias** |  |  |  |  |
| *14. Was an attempt made to blind study subjects to the intervention they have received?* For studies where the patients would have no way of knowing which  intervention they received, this should be answered yes. |  |  | no |  |
| *15. Was an attempt made to blind those measuring the main outcomes of the intervention?* |  |  | no |  |
| *16. If any of the results of the study were based on “data dredging”, was this made clear?* Any analyses that had not been planned at the outset of the study should be clearly indicated. If no retrospective unplanned subgroup analyses were reported, then answer yes. |  |  | yes |  |
| *17. In trials and cohort studies, do the analyses adjust for different lengths of follow‐up of patients, or in case‐control studies, is the time period between the intervention and outcome the same for cases and controls?*Where follow‐up was the same for all study patients the answer should yes. If different lengths of follow‐up were adjusted for by, for example, survival analysis the answer should be yes. Studies where differences in follow‐up are ignored should be answered no. | unable to determine |  |  |  |
| 1*8. Were the statistical tests used to assess the main outcomes appropriate?* The statistical techniques used must be appropriate to the data. For example nonparametric methods should be used for small sample sizes. Where little statistical analysis has been undertaken but where there is no evidence of bias, the question should be answered yes. If the distribution of the data (normal or not) is not described it must be assumed that the estimates used were appropriate and the question should be answered yes. |  |  | yes |  |
| *19. Was compliance with the intervention/s reliable*? Where there was non compliance with the allocated treatment or where there was contamination of one group, the question should be answered no. For studies where the effect of any misclassification was likely to bias any association to the null, the question should be answered yes |  |  | yes |  |
| *20. Were the main outcome measures used accurate (valid and reliable)?* For studies where the outcome measures are clearly described, the question should be answered yes. For studies which refer to other work or that demonstrates the outcome measures are accurate, the question should be answered as yes. |  |  | yes |  |
| **Internal validity ‐ confounding (selection bias)** |  |  |  |  |
| *21. Were the patients in different intervention groups (trials and cohort studies) or were the cases and controls (case‐control studies) recruited from the same population?* For example, patients for all comparison groups should be selected from the same hospital. The question should be answered unable to determine for cohort and casecontrol studies where there is no information concerning the source of patients included in the study. | unable to determine |  |  |  |
| *22. Were study subjects in different intervention groups (trials and cohort studies) or were the cases and controls (case‐control studies) recruited over the same period of time?* For a study which does not specify the time period over which patients were recruited, the question should be answered as unable to determine. | unable to determine |  |  |  |
| *23. Were study subjects randomised to intervention groups?* Studies which state that subjects were randomized should be answered yes except where method of randomisation would not ensure random allocation. For example alternate allocation would score no because it is predictable. |  | no |  |  |
| *24. Was the randomised intervention assignment concealed from both patients and health care staff until recruitment was complete and irrevocable?* All non‐randomised studies should be answered no. If assignment was oncealed from patients but not from staff, it should be answered no. |  | no |  |  |
| *25. Was there adequate adjustment for confounding in the analyses from which the main findings were drawn?* This question should be answered no for trials if: the main conclusions of the study were based on analyses of treatment rather than intention to treat; the distribution of known confounders in the different treatment groups was not described; or the distribution of known confounders differed between the treatment groups but was not taken into account in the analyses. In nonrandomized studies if the effect of the main confounders was not investigated or confounding was demonstrated but no adjustment was made in the final analyses the question should be answered as no. |  | no |  |  |
| *26. Were losses of patients to follow‐up taken into account?* If the numbers of patients lost to follow‐up are not reported, the question should be answered as unable to determine. If the proportion lost to follow‐ up was too small to affect the main findings, the question should be answered yes. | unable to determine |  |  |  |
| **Power** |  |  |  |  |
| *27. Did the study have sufficient power to detect a clinically important effect where the probability value for a difference being due to chance is less than 5%?*Sample sizes have been calculated to detect a difference of x% and y%. |  | no |  |  |
| **Total score:** |  |  | 14 |  |
|  |  |  |  |  |
| **Article Author & Name: Vgontzas et al. 1999** | unable to determine | no | yes | **Notes/Justification** |
| **Reporting** | 0 | 0 | 1 |  |
| *1. Is the hypothesis/aim/objective of the study clearly described?* |  |  | yes |  |
| *2. Are the main outcomes to be measured clearly described in the Introduction or Methods section?* If the main outcomes are first mentioned in the Results section, the question should be answered no |  |  | yes |  |
| *3. Are the characteristics of the patients included in the study clearly described ?* In cohort studies and trials, inclusion and/or exclusion criteria should be given. In case‐control studies, a case‐definition and the source for controls should be given. |  |  | yes |  |
| *4. Are the interventions of interest clearly described?*Treatments and placebo (where relevant) that are to be compared should be clearly described. |  |  | yes |  |
| *5. Are the distributions of principal confounders in each group of subjects to be compared clearly described?* A list of principal confounders is provided. |  |  | yes (2) |  |
| *6. Are the main findings of the study clearly described?* Simple outcome data (including denominators and numerators) should be reported for all major findings so that the reader can check the major analyses and conclusions. (This question does not cover statistical tests which are considered below). |  |  | yes |  |
| *7. Does the study provide estimates of the random variability in the data for the main outcomes?* In non normally distributed data the inter‐quartile range of results should be reported. In normally distributed data the standard error, standard deviation or confidence intervals should be reported. If the distribution of the data is not described, it must be assumed that the estimates used were appropriate and the question should be answered yes. |  |  | yes |  |
| *8. Have all important adverse events that may be a consequence of the intervention been reported?* This should be answered yes if the study demonstrates that there was a comprehensive attempt to measure adverse events. (A list of possible adverse events is provided). |  | no |  |  |
| *9. Have the characteristics of patients lost to follow‐up been described?* This should be answered yes where there were no losses to follow‐up or where losses to follow‐up were so small that findings would be unaffected by their inclusion. This should be answered no where a study does not report the number of patients lost to follow‐up. |  | no |  |  |
| *10. Have actual probability values been reported ( e.g. 0.035 rather than <0.05) for the main outcomes except where the probability value is less than 0.001?* |  | no |  |  |
| **External Validity** All the following criteria attempt to address the representativeness of the findings of the study and whether they may be generalised to the population from which the study subjects were derived. |  |  |  |  |
| *11. Were the subjects asked to participate in the study representative of the entire population from which they were recruited?* The study must identify the source population for patients and describe how the patients were selected. Patients would be representative if they comprised the entire source population, an unselected sample of consecutive patients, or a random sample. Random sampling is only feasible where a list of all members of the relevant population exists. Where a study does not report the proportion of the source population from which the patients are derived, the question should be answered as unable to determine. |  |  | yes |  |
| *12. Were those subjects who were prepared to participate representative of the entire population from which they were recruited?*The proportion of those asked who agreed should be stated. Validation that the sample was representative would include demonstrating that the distribution of the main confounding factors was the same in the study sample and the source population. | unable to determine |  |  |  |
| *13. Were the staff, places, and facilities where the patients were treated, representative of the treatment the majority of patients receive?* For the question to be answered yes the study should demonstrate that the intervention was representative of that in use in the source population. The question should be answered no if, for example, the intervention was undertaken in a specialist centre unrepresentative of the hospitals most of the source population would attend. | unable to determine |  |  |  |
| **Internal validity – bias** |  |  |  |  |
| *14. Was an attempt made to blind study subjects to the intervention they have received?* For studies where the patients would have no way of knowing which  intervention they received, this should be answered yes. |  | no |  |  |
| *15. Was an attempt made to blind those measuring the main outcomes of the intervention?* |  | no |  |  |
| *16. If any of the results of the study were based on “data dredging”, was this made clear?* Any analyses that had not been planned at the outset of the study should be clearly indicated. If no retrospective unplanned subgroup analyses were reported, then answer yes. |  |  | yes |  |
| *17. In trials and cohort studies, do the analyses adjust for different lengths of follow‐up of patients, or in case‐control studies, is the time period between the intervention and outcome the same for cases and controls?*Where follow‐up was the same for all study patients the answer should yes. If different lengths of follow‐up were adjusted for by, for example, survival analysis the answer should be yes. Studies where differences in follow‐up are ignored should be answered no. |  |  | yes |  |
| 1*8. Were the statistical tests used to assess the main outcomes appropriate?* The statistical techniques used must be appropriate to the data. For example nonparametric methods should be used for small sample sizes. Where little statistical analysis has been undertaken but where there is no evidence of bias, the question should be answered yes. If the distribution of the data (normal or not) is not described it must be assumed that the estimates used were appropriate and the question should be answered yes. |  |  | yes |  |
| *19. Was compliance with the intervention/s reliable*? Where there was non compliance with the allocated treatment or where there was contamination of one group, the question should be answered no. For studies where the effect of any misclassification was likely to bias any association to the null, the question should be answered yes |  |  | yes |  |
| *20. Were the main outcome measures used accurate (valid and reliable)?* For studies where the outcome measures are clearly described, the question should be answered yes. For studies which refer to other work or that demonstrates the outcome measures are accurate, the question should be answered as yes. |  |  | yes |  |
| **Internal validity ‐ confounding (selection bias)** |  |  |  |  |
| *21. Were the patients in different intervention groups (trials and cohort studies) or were the cases and controls (case‐control studies) recruited from the same population?* For example, patients for all comparison groups should be selected from the same hospital. The question should be answered unable to determine for cohort and casecontrol studies where there is no information concerning the source of patients included in the study. |  |  | yes |  |
| *22. Were study subjects in different intervention groups (trials and cohort studies) or were the cases and controls (case‐control studies) recruited over the same period of time?* For a study which does not specify the time period over which patients were recruited, the question should be answered as unable to determine. | unable to determine |  |  |  |
| *23. Were study subjects randomised to intervention groups?* Studies which state that subjects were randomized should be answered yes except where method of randomisation would not ensure random allocation. For example alternate allocation would score no because it is predictable. |  | no |  |  |
| *24. Was the randomised intervention assignment concealed from both patients and health care staff until recruitment was complete and irrevocable?* All non‐randomised studies should be answered no. If assignment was oncealed from patients but not from staff, it should be answered no. |  | no |  |  |
| *25. Was there adequate adjustment for confounding in the analyses from which the main findings were drawn?* This question should be answered no for trials if: the main conclusions of the study were based on analyses of treatment rather than intention to treat; the distribution of known confounders in the different treatment groups was not described; or the distribution of known confounders differed between the treatment groups but was not taken into account in the analyses. In nonrandomized studies if the effect of the main confounders was not investigated or confounding was demonstrated but no adjustment was made in the final analyses the question should be answered as no. |  | no |  |  |
| *26. Were losses of patients to follow‐up taken into account?* If the numbers of patients lost to follow‐up are not reported, the question should be answered as unable to determine. If the proportion lost to follow‐ up was too small to affect the main findings, the question should be answered yes. | unable to determine |  |  |  |
| **Power** |  |  |  |  |
| *27. Did the study have sufficient power to detect a clinically important effect where the probability value for a difference being due to chance is less than 5%?*Sample sizes have been calculated to detect a difference of x% and y%. |  | no |  | no a priori power analysis was conducted, unable to determine effect size of interested outcome |
| **Total score:** |  |  | 15 |  |
|  |  |  |  |  |
| **Article Author & Name: Vgontzas et al. 2004** | unable to determine | no | yes | **Notes/Justification** |
| **Reporting** | 0 | 0 | 1 |  |
| *1. Is the hypothesis/aim/objective of the study clearly described?* |  |  | yes |  |
| *2. Are the main outcomes to be measured clearly described in the Introduction or Methods section?* If the main outcomes are first mentioned in the Results section, the question should be answered no |  |  | yes |  |
| *3. Are the characteristics of the patients included in the study clearly described ?* In cohort studies and trials, inclusion and/or exclusion criteria should be given. In case‐control studies, a case‐definition and the source for controls should be given. |  |  | yes |  |
| *4. Are the interventions of interest clearly described?*Treatments and placebo (where relevant) that are to be compared should be clearly described. |  |  | yes |  |
| *5. Are the distributions of principal confounders in each group of subjects to be compared clearly described?* A list of principal confounders is provided. |  |  | yes (2) |  |
| *6. Are the main findings of the study clearly described?* Simple outcome data (including denominators and numerators) should be reported for all major findings so that the reader can check the major analyses and conclusions. (This question does not cover statistical tests which are considered below). |  |  | yes |  |
| *7. Does the study provide estimates of the random variability in the data for the main outcomes?* In non normally distributed data the inter‐quartile range of results should be reported. In normally distributed data the standard error, standard deviation or confidence intervals should be reported. If the distribution of the data is not described, it must be assumed that the estimates used were appropriate and the question should be answered yes. |  |  | yes |  |
| *8. Have all important adverse events that may be a consequence of the intervention been reported?* This should be answered yes if the study demonstrates that there was a comprehensive attempt to measure adverse events. (A list of possible adverse events is provided). |  | no |  |  |
| *9. Have the characteristics of patients lost to follow‐up been described?* This should be answered yes where there were no losses to follow‐up or where losses to follow‐up were so small that findings would be unaffected by their inclusion. This should be answered no where a study does not report the number of patients lost to follow‐up. |  | no |  |  |
| *10. Have actual probability values been reported ( e.g. 0.035 rather than <0.05) for the main outcomes except where the probability value is less than 0.001?* |  |  | yes |  |
| **External Validity** All the following criteria attempt to address the representativeness of the findings of the study and whether they may be generalised to the population from which the study subjects were derived. |  |  |  |  |
| *11. Were the subjects asked to participate in the study representative of the entire population from which they were recruited?* The study must identify the source population for patients and describe how the patients were selected. Patients would be representative if they comprised the entire source population, an unselected sample of consecutive patients, or a random sample. Random sampling is only feasible where a list of all members of the relevant population exists. Where a study does not report the proportion of the source population from which the patients are derived, the question should be answered as unable to determine. | unable to determine |  |  |  |
| *12. Were those subjects who were prepared to participate representative of the entire population from which they were recruited?*The proportion of those asked who agreed should be stated. Validation that the sample was representative would include demonstrating that the distribution of the main confounding factors was the same in the study sample and the source population. | unable to determine |  |  |  |
| *13. Were the staff, places, and facilities where the patients were treated, representative of the treatment the majority of patients receive?* For the question to be answered yes the study should demonstrate that the intervention was representative of that in use in the source population. The question should be answered no if, for example, the intervention was undertaken in a specialist centre unrepresentative of the hospitals most of the source population would attend. | unable to determine |  |  |  |
| **Internal validity – bias** |  |  |  |  |
| *14. Was an attempt made to blind study subjects to the intervention they have received?* For studies where the patients would have no way of knowing which  intervention they received, this should be answered yes. |  | no |  |  |
| *15. Was an attempt made to blind those measuring the main outcomes of the intervention?* |  | no |  |  |
| *16. If any of the results of the study were based on “data dredging”, was this made clear?* Any analyses that had not been planned at the outset of the study should be clearly indicated. If no retrospective unplanned subgroup analyses were reported, then answer yes. |  |  | yes |  |
| *17. In trials and cohort studies, do the analyses adjust for different lengths of follow‐up of patients, or in case‐control studies, is the time period between the intervention and outcome the same for cases and controls?*Where follow‐up was the same for all study patients the answer should yes. If different lengths of follow‐up were adjusted for by, for example, survival analysis the answer should be yes. Studies where differences in follow‐up are ignored should be answered no. |  |  | yes |  |
| 1*8. Were the statistical tests used to assess the main outcomes appropriate?* The statistical techniques used must be appropriate to the data. For example nonparametric methods should be used for small sample sizes. Where little statistical analysis has been undertaken but where there is no evidence of bias, the question should be answered yes. If the distribution of the data (normal or not) is not described it must be assumed that the estimates used were appropriate and the question should be answered yes. |  |  | yes |  |
| *19. Was compliance with the intervention/s reliable*? Where there was non compliance with the allocated treatment or where there was contamination of one group, the question should be answered no. For studies where the effect of any misclassification was likely to bias any association to the null, the question should be answered yes |  |  | yes |  |
| *20. Were the main outcome measures used accurate (valid and reliable)?* For studies where the outcome measures are clearly described, the question should be answered yes. For studies which refer to other work or that demonstrates the outcome measures are accurate, the question should be answered as yes. |  |  | yes |  |
| **Internal validity ‐ confounding (selection bias)** |  |  |  |  |
| *21. Were the patients in different intervention groups (trials and cohort studies) or were the cases and controls (case‐control studies) recruited from the same population?* For example, patients for all comparison groups should be selected from the same hospital. The question should be answered unable to determine for cohort and casecontrol studies where there is no information concerning the source of patients included in the study. |  |  | yes |  |
| *22. Were study subjects in different intervention groups (trials and cohort studies) or were the cases and controls (case‐control studies) recruited over the same period of time?* For a study which does not specify the time period over which patients were recruited, the question should be answered as unable to determine. | unable to determine |  |  |  |
| *23. Were study subjects randomised to intervention groups?* Studies which state that subjects were randomized should be answered yes except where method of randomisation would not ensure random allocation. For example alternate allocation would score no because it is predictable. |  | no |  |  |
| *24. Was the randomised intervention assignment concealed from both patients and health care staff until recruitment was complete and irrevocable?* All non‐randomised studies should be answered no. If assignment was oncealed from patients but not from staff, it should be answered no. |  | no |  |  |
| *25. Was there adequate adjustment for confounding in the analyses from which the main findings were drawn?* This question should be answered no for trials if: the main conclusions of the study were based on analyses of treatment rather than intention to treat; the distribution of known confounders in the different treatment groups was not described; or the distribution of known confounders differed between the treatment groups but was not taken into account in the analyses. In nonrandomized studies if the effect of the main confounders was not investigated or confounding was demonstrated but no adjustment was made in the final analyses the question should be answered as no. |  | no |  |  |
| *26. Were losses of patients to follow‐up taken into account?* If the numbers of patients lost to follow‐up are not reported, the question should be answered as unable to determine. If the proportion lost to follow‐ up was too small to affect the main findings, the question should be answered yes. | unable to determine |  |  |  |
| **Power** |  |  |  |  |
| *27. Did the study have sufficient power to detect a clinically important effect where the probability value for a difference being due to chance is less than 5%?*Sample sizes have been calculated to detect a difference of x% and y%. |  | no |  | no a priori power analysis was conducted, unable to determine effect size of interested outcome |
| **Total score:** |  |  | 15 |  |
|  |  |  |  |  |
| **Article Author & Name: Vgontzas et al. 2007** | unable to determine | no | yes | **Notes/Justification** |
| **Reporting** | 0 | 0 | 1 |  |
| *1. Is the hypothesis/aim/objective of the study clearly described?* |  |  | yes |  |
| *2. Are the main outcomes to be measured clearly described in the Introduction or Methods section?* If the main outcomes are first mentioned in the Results section, the question should be answered no |  |  | yes |  |
| *3. Are the characteristics of the patients included in the study clearly described ?* In cohort studies and trials, inclusion and/or exclusion criteria should be given. In case‐control studies, a case‐definition and the source for controls should be given. |  |  | yes |  |
| *4. Are the interventions of interest clearly described?*Treatments and placebo (where relevant) that are to be compared should be clearly described. |  |  | yes |  |
| *5. Are the distributions of principal confounders in each group of subjects to be compared clearly described?* A list of principal confounders is provided. |  |  | yes (2) |  |
| *6. Are the main findings of the study clearly described?* Simple outcome data (including denominators and numerators) should be reported for all major findings so that the reader can check the major analyses and conclusions. (This question does not cover statistical tests which are considered below). |  |  | yes |  |
| *7. Does the study provide estimates of the random variability in the data for the main outcomes?* In non normally distributed data the inter‐quartile range of results should be reported. In normally distributed data the standard error, standard deviation or confidence intervals should be reported. If the distribution of the data is not described, it must be assumed that the estimates used were appropriate and the question should be answered yes. |  |  | yes |  |
| *8. Have all important adverse events that may be a consequence of the intervention been reported?* This should be answered yes if the study demonstrates that there was a comprehensive attempt to measure adverse events. (A list of possible adverse events is provided). |  | no |  |  |
| *9. Have the characteristics of patients lost to follow‐up been described?* This should be answered yes where there were no losses to follow‐up or where losses to follow‐up were so small that findings would be unaffected by their inclusion. This should be answered no where a study does not report the number of patients lost to follow‐up. |  | no |  |  |
| *10. Have actual probability values been reported ( e.g. 0.035 rather than <0.05) for the main outcomes except where the probability value is less than 0.001?* |  | no |  |  |
| **External Validity** All the following criteria attempt to address the representativeness of the findings of the study and whether they may be generalised to the population from which the study subjects were derived. |  |  |  |  |
| *11. Were the subjects asked to participate in the study representative of the entire population from which they were recruited?* The study must identify the source population for patients and describe how the patients were selected. Patients would be representative if they comprised the entire source population, an unselected sample of consecutive patients, or a random sample. Random sampling is only feasible where a list of all members of the relevant population exists. Where a study does not report the proportion of the source population from which the patients are derived, the question should be answered as unable to determine. |  |  | yes |  |
| *12. Were those subjects who were prepared to participate representative of the entire population from which they were recruited?*The proportion of those asked who agreed should be stated. Validation that the sample was representative would include demonstrating that the distribution of the main confounding factors was the same in the study sample and the source population. | unable to determine |  |  |  |
| *13. Were the staff, places, and facilities where the patients were treated, representative of the treatment the majority of patients receive?* For the question to be answered yes the study should demonstrate that the intervention was representative of that in use in the source population. The question should be answered no if, for example, the intervention was undertaken in a specialist centre unrepresentative of the hospitals most of the source population would attend. | unable to determine |  |  |  |
| **Internal validity – bias** |  |  |  |  |
| *14. Was an attempt made to blind study subjects to the intervention they have received?* For studies where the patients would have no way of knowing which  intervention they received, this should be answered yes. |  | no |  |  |
| *15. Was an attempt made to blind those measuring the main outcomes of the intervention?* |  | no |  |  |
| *16. If any of the results of the study were based on “data dredging”, was this made clear?* Any analyses that had not been planned at the outset of the study should be clearly indicated. If no retrospective unplanned subgroup analyses were reported, then answer yes. |  |  | yes |  |
| *17. In trials and cohort studies, do the analyses adjust for different lengths of follow‐up of patients, or in case‐control studies, is the time period between the intervention and outcome the same for cases and controls?*Where follow‐up was the same for all study patients the answer should yes. If different lengths of follow‐up were adjusted for by, for example, survival analysis the answer should be yes. Studies where differences in follow‐up are ignored should be answered no. |  |  | yes |  |
| 1*8. Were the statistical tests used to assess the main outcomes appropriate?* The statistical techniques used must be appropriate to the data. For example nonparametric methods should be used for small sample sizes. Where little statistical analysis has been undertaken but where there is no evidence of bias, the question should be answered yes. If the distribution of the data (normal or not) is not described it must be assumed that the estimates used were appropriate and the question should be answered yes. |  |  | yes |  |
| *19. Was compliance with the intervention/s reliable*? Where there was non compliance with the allocated treatment or where there was contamination of one group, the question should be answered no. For studies where the effect of any misclassification was likely to bias any association to the null, the question should be answered yes |  |  | yes |  |
| *20. Were the main outcome measures used accurate (valid and reliable)?* For studies where the outcome measures are clearly described, the question should be answered yes. For studies which refer to other work or that demonstrates the outcome measures are accurate, the question should be answered as yes. |  |  | yes |  |
| **Internal validity ‐ confounding (selection bias)** |  |  |  |  |
| *21. Were the patients in different intervention groups (trials and cohort studies) or were the cases and controls (case‐control studies) recruited from the same population?* For example, patients for all comparison groups should be selected from the same hospital. The question should be answered unable to determine for cohort and casecontrol studies where there is no information concerning the source of patients included in the study. |  |  | yes |  |
| *22. Were study subjects in different intervention groups (trials and cohort studies) or were the cases and controls (case‐control studies) recruited over the same period of time?* For a study which does not specify the time period over which patients were recruited, the question should be answered as unable to determine. | unable to determine |  |  |  |
| *23. Were study subjects randomised to intervention groups?* Studies which state that subjects were randomized should be answered yes except where method of randomisation would not ensure random allocation. For example alternate allocation would score no because it is predictable. |  | no |  |  |
| *24. Was the randomised intervention assignment concealed from both patients and health care staff until recruitment was complete and irrevocable?* All non‐randomised studies should be answered no. If assignment was oncealed from patients but not from staff, it should be answered no. |  | no |  |  |
| *25. Was there adequate adjustment for confounding in the analyses from which the main findings were drawn?* This question should be answered no for trials if: the main conclusions of the study were based on analyses of treatment rather than intention to treat; the distribution of known confounders in the different treatment groups was not described; or the distribution of known confounders differed between the treatment groups but was not taken into account in the analyses. In nonrandomized studies if the effect of the main confounders was not investigated or confounding was demonstrated but no adjustment was made in the final analyses the question should be answered as no. |  | no |  |  |
| *26. Were losses of patients to follow‐up taken into account?* If the numbers of patients lost to follow‐up are not reported, the question should be answered as unable to determine. If the proportion lost to follow‐ up was too small to affect the main findings, the question should be answered yes. | unable to determine |  |  |  |
| **Power** |  |  |  |  |
| *27. Did the study have sufficient power to detect a clinically important effect where the probability value for a difference being due to chance is less than 5%?*Sample sizes have been calculated to detect a difference of x% and y%. |  | no |  | no a priori power analysis was conducted, unable to determine effect size of interested outcome |
| **Total score:** |  |  | 15 |  |
|  |  |  |  |  |
| **Article Author & Name: Wolkow et al. 2015 b** | unable to determine | no | yes | **Notes/Justification** |
| **Reporting** | 0 | 0 | 1 |  |
| *1. Is the hypothesis/aim/objective of the study clearly described?* |  |  | yes |  |
| *2. Are the main outcomes to be measured clearly described in the Introduction or Methods section?* If the main outcomes are first mentioned in the Results section, the question should be answered no |  |  | yes |  |
| *3. Are the characteristics of the patients included in the study clearly described ?* In cohort studies and trials, inclusion and/or exclusion criteria should be given. In case‐control studies, a case‐definition and the source for controls should be given. |  |  | yes |  |
| *4. Are the interventions of interest clearly described?*Treatments and placebo (where relevant) that are to be compared should be clearly described. |  |  | yes |  |
| *5. Are the distributions of principal confounders in each group of subjects to be compared clearly described?* A list of principal confounders is provided. |  |  | yes (2) |  |
| *6. Are the main findings of the study clearly described?* Simple outcome data (including denominators and numerators) should be reported for all major findings so that the reader can check the major analyses and conclusions. (This question does not cover statistical tests which are considered below). |  |  | yes |  |
| *7. Does the study provide estimates of the random variability in the data for the main outcomes?* In non normally distributed data the inter‐quartile range of results should be reported. In normally distributed data the standard error, standard deviation or confidence intervals should be reported. If the distribution of the data is not described, it must be assumed that the estimates used were appropriate and the question should be answered yes. |  |  | yes |  |
| *8. Have all important adverse events that may be a consequence of the intervention been reported?* This should be answered yes if the study demonstrates that there was a comprehensive attempt to measure adverse events. (A list of possible adverse events is provided). |  | no |  |  |
| *9. Have the characteristics of patients lost to follow‐up been described?* This should be answered yes where there were no losses to follow‐up or where losses to follow‐up were so small that findings would be unaffected by their inclusion. This should be answered no where a study does not report the number of patients lost to follow‐up. |  | no |  |  |
| *10. Have actual probability values been reported ( e.g. 0.035 rather than <0.05) for the main outcomes except where the probability value is less than 0.001?* |  |  | yes |  |
| **External Validity** All the following criteria attempt to address the representativeness of the findings of the study and whether they may be generalised to the population from which the study subjects were derived. |  |  |  |  |
| *11. Were the subjects asked to participate in the study representative of the entire population from which they were recruited?* The study must identify the source population for patients and describe how the patients were selected. Patients would be representative if they comprised the entire source population, an unselected sample of consecutive patients, or a random sample. Random sampling is only feasible where a list of all members of the relevant population exists. Where a study does not report the proportion of the source population from which the patients are derived, the question should be answered as unable to determine. |  |  | yes |  |
| *12. Were those subjects who were prepared to participate representative of the entire population from which they were recruited?*The proportion of those asked who agreed should be stated. Validation that the sample was representative would include demonstrating that the distribution of the main confounding factors was the same in the study sample and the source population. | unable to determine |  |  |  |
| *13. Were the staff, places, and facilities where the patients were treated, representative of the treatment the majority of patients receive?* For the question to be answered yes the study should demonstrate that the intervention was representative of that in use in the source population. The question should be answered no if, for example, the intervention was undertaken in a specialist centre unrepresentative of the hospitals most of the source population would attend. | unable to determine |  |  |  |
| **Internal validity – bias** |  |  |  |  |
| *14. Was an attempt made to blind study subjects to the intervention they have received?* For studies where the patients would have no way of knowing which  intervention they received, this should be answered yes. |  | no |  |  |
| *15. Was an attempt made to blind those measuring the main outcomes of the intervention?* |  | no |  |  |
| *16. If any of the results of the study were based on “data dredging”, was this made clear?* Any analyses that had not been planned at the outset of the study should be clearly indicated. If no retrospective unplanned subgroup analyses were reported, then answer yes. |  |  | yes |  |
| *17. In trials and cohort studies, do the analyses adjust for different lengths of follow‐up of patients, or in case‐control studies, is the time period between the intervention and outcome the same for cases and controls?*Where follow‐up was the same for all study patients the answer should yes. If different lengths of follow‐up were adjusted for by, for example, survival analysis the answer should be yes. Studies where differences in follow‐up are ignored should be answered no. |  |  | yes |  |
| 1*8. Were the statistical tests used to assess the main outcomes appropriate?* The statistical techniques used must be appropriate to the data. For example nonparametric methods should be used for small sample sizes. Where little statistical analysis has been undertaken but where there is no evidence of bias, the question should be answered yes. If the distribution of the data (normal or not) is not described it must be assumed that the estimates used were appropriate and the question should be answered yes. |  |  | yes |  |
| *19. Was compliance with the intervention/s reliable*? Where there was non compliance with the allocated treatment or where there was contamination of one group, the question should be answered no. For studies where the effect of any misclassification was likely to bias any association to the null, the question should be answered yes |  |  | yes |  |
| *20. Were the main outcome measures used accurate (valid and reliable)?* For studies where the outcome measures are clearly described, the question should be answered yes. For studies which refer to other work or that demonstrates the outcome measures are accurate, the question should be answered as yes. |  |  | yes |  |
| **Internal validity ‐ confounding (selection bias)** |  |  |  |  |
| *21. Were the patients in different intervention groups (trials and cohort studies) or were the cases and controls (case‐control studies) recruited from the same population?* For example, patients for all comparison groups should be selected from the same hospital. The question should be answered unable to determine for cohort and casecontrol studies where there is no information concerning the source of patients included in the study. |  |  | yes |  |
| *22. Were study subjects in different intervention groups (trials and cohort studies) or were the cases and controls (case‐control studies) recruited over the same period of time?* For a study which does not specify the time period over which patients were recruited, the question should be answered as unable to determine. | unable to determine |  |  |  |
| *23. Were study subjects randomised to intervention groups?* Studies which state that subjects were randomized should be answered yes except where method of randomisation would not ensure random allocation. For example alternate allocation would score no because it is predictable. |  |  | yes |  |
| *24. Was the randomised intervention assignment concealed from both patients and health care staff until recruitment was complete and irrevocable?* All non‐randomised studies should be answered no. If assignment was oncealed from patients but not from staff, it should be answered no. | unable to determine |  |  |  |
| *25. Was there adequate adjustment for confounding in the analyses from which the main findings were drawn?* This question should be answered no for trials if: the main conclusions of the study were based on analyses of treatment rather than intention to treat; the distribution of known confounders in the different treatment groups was not described; or the distribution of known confounders differed between the treatment groups but was not taken into account in the analyses. In nonrandomized studies if the effect of the main confounders was not investigated or confounding was demonstrated but no adjustment was made in the final analyses the question should be answered as no. |  |  | yes |  |
| *26. Were losses of patients to follow‐up taken into account?* If the numbers of patients lost to follow‐up are not reported, the question should be answered as unable to determine. If the proportion lost to follow‐ up was too small to affect the main findings, the question should be answered yes. | unable to determine |  |  |  |
| **Power** |  |  |  |  |
| *27. Did the study have sufficient power to detect a clinically important effect where the probability value for a difference being due to chance is less than 5%?*Sample sizes have been calculated to detect a difference of x% and y%. |  | no |  | no a priori power analysis was conducted, unable to determine effect size of interested outcome |
| **Total score:** |  |  | 17 |  |
|  |  |  |  |  |
| **Article Author & Name: Yamazaki et al. 2021** | unable to determine | no | yes | **Notes/Justification** |
| **Reporting** | 0 | 0 | 1 |  |
| *1. Is the hypothesis/aim/objective of the study clearly described?* |  |  | yes |  |
| *2. Are the main outcomes to be measured clearly described in the Introduction or Methods section?* If the main outcomes are first mentioned in the Results section, the question should be answered no |  |  | yes |  |
| *3. Are the characteristics of the patients included in the study clearly described ?* In cohort studies and trials, inclusion and/or exclusion criteria should be given. In case‐control studies, a case‐definition and the source for controls should be given. |  |  | yes |  |
| *4. Are the interventions of interest clearly described?*Treatments and placebo (where relevant) that are to be compared should be clearly described. |  |  | yes |  |
| *5. Are the distributions of principal confounders in each group of subjects to be compared clearly described?* A list of principal confounders is provided. |  |  | yes (2) |  |
| *6. Are the main findings of the study clearly described?* Simple outcome data (including denominators and numerators) should be reported for all major findings so that the reader can check the major analyses and conclusions. (This question does not cover statistical tests which are considered below). |  |  | yes |  |
| *7. Does the study provide estimates of the random variability in the data for the main outcomes?* In non normally distributed data the inter‐quartile range of results should be reported. In normally distributed data the standard error, standard deviation or confidence intervals should be reported. If the distribution of the data is not described, it must be assumed that the estimates used were appropriate and the question should be answered yes. |  |  | yes |  |
| *8. Have all important adverse events that may be a consequence of the intervention been reported?* This should be answered yes if the study demonstrates that there was a comprehensive attempt to measure adverse events. (A list of possible adverse events is provided). |  | no |  |  |
| *9. Have the characteristics of patients lost to follow‐up been described?* This should be answered yes where there were no losses to follow‐up or where losses to follow‐up were so small that findings would be unaffected by their inclusion. This should be answered no where a study does not report the number of patients lost to follow‐up. |  |  | yes |  |
| *10. Have actual probability values been reported ( e.g. 0.035 rather than <0.05) for the main outcomes except where the probability value is less than 0.001?* |  |  | yes |  |
| **External Validity** All the following criteria attempt to address the representativeness of the findings of the study and whether they may be generalised to the population from which the study subjects were derived. |  |  |  |  |
| *11. Were the subjects asked to participate in the study representative of the entire population from which they were recruited?* The study must identify the source population for patients and describe how the patients were selected. Patients would be representative if they comprised the entire source population, an unselected sample of consecutive patients, or a random sample. Random sampling is only feasible where a list of all members of the relevant population exists. Where a study does not report the proportion of the source population from which the patients are derived, the question should be answered as unable to determine. |  |  | yes |  |
| *12. Were those subjects who were prepared to participate representative of the entire population from which they were recruited?*The proportion of those asked who agreed should be stated. Validation that the sample was representative would include demonstrating that the distribution of the main confounding factors was the same in the study sample and the source population. |  |  | yes |  |
| *13. Were the staff, places, and facilities where the patients were treated, representative of the treatment the majority of patients receive?* For the question to be answered yes the study should demonstrate that the intervention was representative of that in use in the source population. The question should be answered no if, for example, the intervention was undertaken in a specialist centre unrepresentative of the hospitals most of the source population would attend. | unable to determine |  |  |  |
| **Internal validity – bias** |  |  |  |  |
| *14. Was an attempt made to blind study subjects to the intervention they have received?* For studies where the patients would have no way of knowing which  intervention they received, this should be answered yes. |  | no |  |  |
| *15. Was an attempt made to blind those measuring the main outcomes of the intervention?* |  | no |  |  |
| *16. If any of the results of the study were based on “data dredging”, was this made clear?* Any analyses that had not been planned at the outset of the study should be clearly indicated. If no retrospective unplanned subgroup analyses were reported, then answer yes. |  |  | yes |  |
| *17. In trials and cohort studies, do the analyses adjust for different lengths of follow‐up of patients, or in case‐control studies, is the time period between the intervention and outcome the same for cases and controls?*Where follow‐up was the same for all study patients the answer should yes. If different lengths of follow‐up were adjusted for by, for example, survival analysis the answer should be yes. Studies where differences in follow‐up are ignored should be answered no. |  |  | yes |  |
| 1*8. Were the statistical tests used to assess the main outcomes appropriate?* The statistical techniques used must be appropriate to the data. For example nonparametric methods should be used for small sample sizes. Where little statistical analysis has been undertaken but where there is no evidence of bias, the question should be answered yes. If the distribution of the data (normal or not) is not described it must be assumed that the estimates used were appropriate and the question should be answered yes. |  |  | yes |  |
| *19. Was compliance with the intervention/s reliable*? Where there was non compliance with the allocated treatment or where there was contamination of one group, the question should be answered no. For studies where the effect of any misclassification was likely to bias any association to the null, the question should be answered yes |  |  | yes |  |
| *20. Were the main outcome measures used accurate (valid and reliable)?* For studies where the outcome measures are clearly described, the question should be answered yes. For studies which refer to other work or that demonstrates the outcome measures are accurate, the question should be answered as yes. |  |  | yes |  |
| **Internal validity ‐ confounding (selection bias)** |  |  |  |  |
| *21. Were the patients in different intervention groups (trials and cohort studies) or were the cases and controls (case‐control studies) recruited from the same population?* For example, patients for all comparison groups should be selected from the same hospital. The question should be answered unable to determine for cohort and casecontrol studies where there is no information concerning the source of patients included in the study. |  |  | yes |  |
| *22. Were study subjects in different intervention groups (trials and cohort studies) or were the cases and controls (case‐control studies) recruited over the same period of time?* For a study which does not specify the time period over which patients were recruited, the question should be answered as unable to determine. | unable to determine |  |  |  |
| *23. Were study subjects randomised to intervention groups?* Studies which state that subjects were randomized should be answered yes except where method of randomisation would not ensure random allocation. For example alternate allocation would score no because it is predictable. |  | no |  |  |
| *24. Was the randomised intervention assignment concealed from both patients and health care staff until recruitment was complete and irrevocable?* All non‐randomised studies should be answered no. If assignment was oncealed from patients but not from staff, it should be answered no. |  | no |  |  |
| *25. Was there adequate adjustment for confounding in the analyses from which the main findings were drawn?* This question should be answered no for trials if: the main conclusions of the study were based on analyses of treatment rather than intention to treat; the distribution of known confounders in the different treatment groups was not described; or the distribution of known confounders differed between the treatment groups but was not taken into account in the analyses. In nonrandomized studies if the effect of the main confounders was not investigated or confounding was demonstrated but no adjustment was made in the final analyses the question should be answered as no. |  |  | yes |  |
| *26. Were losses of patients to follow‐up taken into account?* If the numbers of patients lost to follow‐up are not reported, the question should be answered as unable to determine. If the proportion lost to follow‐ up was too small to affect the main findings, the question should be answered yes. |  |  | yes |  |
| **Power** |  |  |  |  |
| *27. Did the study have sufficient power to detect a clinically important effect where the probability value for a difference being due to chance is less than 5%?*Sample sizes have been calculated to detect a difference of x% and y%. |  | no |  | no a priori power analysis was conducted, small effect size |
| **Total score:** |  |  | 20 |  |
|  |  |  |  |  |
| **Article Author & Name: Yang et al. 2021** | unable to determine | no | yes | **Notes/Justification** |
| **Reporting** | 0 | 0 | 1 |  |
| *1. Is the hypothesis/aim/objective of the study clearly described?* |  |  | yes |  |
| *2. Are the main outcomes to be measured clearly described in the Introduction or Methods section?* If the main outcomes are first mentioned in the Results section, the question should be answered no |  |  | yes |  |
| *3. Are the characteristics of the patients included in the study clearly described ?* In cohort studies and trials, inclusion and/or exclusion criteria should be given. In case‐control studies, a case‐definition and the source for controls should be given. |  |  | yes |  |
| *4. Are the interventions of interest clearly described?*Treatments and placebo (where relevant) that are to be compared should be clearly described. |  |  | yes |  |
| *5. Are the distributions of principal confounders in each group of subjects to be compared clearly described?* A list of principal confounders is provided. |  |  | yes (2) |  |
| *6. Are the main findings of the study clearly described?* Simple outcome data (including denominators and numerators) should be reported for all major findings so that the reader can check the major analyses and conclusions. (This question does not cover statistical tests which are considered below). |  |  | yes |  |
| *7. Does the study provide estimates of the random variability in the data for the main outcomes?* In non normally distributed data the inter‐quartile range of results should be reported. In normally distributed data the standard error, standard deviation or confidence intervals should be reported. If the distribution of the data is not described, it must be assumed that the estimates used were appropriate and the question should be answered yes. |  |  | yes |  |
| *8. Have all important adverse events that may be a consequence of the intervention been reported?* This should be answered yes if the study demonstrates that there was a comprehensive attempt to measure adverse events. (A list of possible adverse events is provided). |  | no |  |  |
| *9. Have the characteristics of patients lost to follow‐up been described?* This should be answered yes where there were no losses to follow‐up or where losses to follow‐up were so small that findings would be unaffected by their inclusion. This should be answered no where a study does not report the number of patients lost to follow‐up. |  |  | yes |  |
| *10. Have actual probability values been reported ( e.g. 0.035 rather than <0.05) for the main outcomes except where the probability value is less than 0.001?* |  |  | yes |  |
| **External Validity** All the following criteria attempt to address the representativeness of the findings of the study and whether they may be generalised to the population from which the study subjects were derived. |  |  |  |  |
| *11. Were the subjects asked to participate in the study representative of the entire population from which they were recruited?* The study must identify the source population for patients and describe how the patients were selected. Patients would be representative if they comprised the entire source population, an unselected sample of consecutive patients, or a random sample. Random sampling is only feasible where a list of all members of the relevant population exists. Where a study does not report the proportion of the source population from which the patients are derived, the question should be answered as unable to determine. |  |  | yes |  |
| *12. Were those subjects who were prepared to participate representative of the entire population from which they were recruited?*The proportion of those asked who agreed should be stated. Validation that the sample was representative would include demonstrating that the distribution of the main confounding factors was the same in the study sample and the source population. |  |  | yes |  |
| *13. Were the staff, places, and facilities where the patients were treated, representative of the treatment the majority of patients receive?* For the question to be answered yes the study should demonstrate that the intervention was representative of that in use in the source population. The question should be answered no if, for example, the intervention was undertaken in a specialist centre unrepresentative of the hospitals most of the source population would attend. | unable to determine |  |  |  |
| **Internal validity – bias** |  |  |  |  |
| *14. Was an attempt made to blind study subjects to the intervention they have received?* For studies where the patients would have no way of knowing which  intervention they received, this should be answered yes. |  | no |  |  |
| *15. Was an attempt made to blind those measuring the main outcomes of the intervention?* |  | no |  |  |
| *16. If any of the results of the study were based on “data dredging”, was this made clear?* Any analyses that had not been planned at the outset of the study should be clearly indicated. If no retrospective unplanned subgroup analyses were reported, then answer yes. |  |  | yes |  |
| *17. In trials and cohort studies, do the analyses adjust for different lengths of follow‐up of patients, or in case‐control studies, is the time period between the intervention and outcome the same for cases and controls?*Where follow‐up was the same for all study patients the answer should yes. If different lengths of follow‐up were adjusted for by, for example, survival analysis the answer should be yes. Studies where differences in follow‐up are ignored should be answered no. |  |  | yes |  |
| 1*8. Were the statistical tests used to assess the main outcomes appropriate?* The statistical techniques used must be appropriate to the data. For example nonparametric methods should be used for small sample sizes. Where little statistical analysis has been undertaken but where there is no evidence of bias, the question should be answered yes. If the distribution of the data (normal or not) is not described it must be assumed that the estimates used were appropriate and the question should be answered yes. |  |  | yes |  |
| *19. Was compliance with the intervention/s reliable*? Where there was non compliance with the allocated treatment or where there was contamination of one group, the question should be answered no. For studies where the effect of any misclassification was likely to bias any association to the null, the question should be answered yes |  |  | yes |  |
| *20. Were the main outcome measures used accurate (valid and reliable)?* For studies where the outcome measures are clearly described, the question should be answered yes. For studies which refer to other work or that demonstrates the outcome measures are accurate, the question should be answered as yes. |  |  |  |  |
| **Internal validity ‐ confounding (selection bias)** |  |  |  |  |
| *21. Were the patients in different intervention groups (trials and cohort studies) or were the cases and controls (case‐control studies) recruited from the same population?* For example, patients for all comparison groups should be selected from the same hospital. The question should be answered unable to determine for cohort and casecontrol studies where there is no information concerning the source of patients included in the study. |  |  | yes |  |
| *22. Were study subjects in different intervention groups (trials and cohort studies) or were the cases and controls (case‐control studies) recruited over the same period of time?* For a study which does not specify the time period over which patients were recruited, the question should be answered as unable to determine. | unable to determine |  |  |  |
| *23. Were study subjects randomised to intervention groups?* Studies which state that subjects were randomized should be answered yes except where method of randomisation would not ensure random allocation. For example alternate allocation would score no because it is predictable. |  |  | yes |  |
| *24. Was the randomised intervention assignment concealed from both patients and health care staff until recruitment was complete and irrevocable?* All non‐randomised studies should be answered no. If assignment was oncealed from patients but not from staff, it should be answered no. | unable to determine |  |  |  |
| *25. Was there adequate adjustment for confounding in the analyses from which the main findings were drawn?* This question should be answered no for trials if: the main conclusions of the study were based on analyses of treatment rather than intention to treat; the distribution of known confounders in the different treatment groups was not described; or the distribution of known confounders differed between the treatment groups but was not taken into account in the analyses. In nonrandomized studies if the effect of the main confounders was not investigated or confounding was demonstrated but no adjustment was made in the final analyses the question should be answered as no. |  |  | yes |  |
| *26. Were losses of patients to follow‐up taken into account?* If the numbers of patients lost to follow‐up are not reported, the question should be answered as unable to determine. If the proportion lost to follow‐ up was too small to affect the main findings, the question should be answered yes. |  |  | yes |  |
| **Power** |  |  |  |  |
| *27. Did the study have sufficient power to detect a clinically important effect where the probability value for a difference being due to chance is less than 5%?*Sample sizes have been calculated to detect a difference of x% and y%. |  | no |  | no a priori power analysis was conducted, unable to determine effect size of interested outcome |
| **Total score:** |  |  | 20 |  |

1. **PRISMA Checklist**

| **Section and Topic** | **Item #** | **Checklist item** | **Location where item is reported** |
| --- | --- | --- | --- |
| **TITLE** | | |  |
| Title | 1 | Identify the report as a systematic review. | 1 |
| **ABSTRACT** | | |  |
| Abstract | 2 | See the PRISMA 2020 for Abstracts checklist. | 3 |
| **INTRODUCTION** | | |  |
| Rationale | 3 | Describe the rationale for the review in the context of existing knowledge. | 4-5 |
| Objectives | 4 | Provide an explicit statement of the objective(s) or question(s) the review addresses. | 5 |
| **METHODS** | | |  |
| Eligibility criteria | 5 | Specify the inclusion and exclusion criteria for the review and how studies were grouped for the syntheses. | 6 |
| Information sources | 6 | Specify all databases, registers, websites, organisations, reference lists and other sources searched or consulted to identify studies. Specify the date when each source was last searched or consulted. | 5 |
| Search strategy | 7 | Present the full search strategies for all databases, registers and websites, including any filters and limits used. | 5 |
| Selection process | 8 | Specify the methods used to decide whether a study met the inclusion criteria of the review, including how many reviewers screened each record and each report retrieved, whether they worked independently, and if applicable, details of automation tools used in the process. | 5-6 |
| Data collection process | 9 | Specify the methods used to collect data from reports, including how many reviewers collected data from each report, whether they worked independently, any processes for obtaining or confirming data from study investigators, and if applicable, details of automation tools used in the process. | 6-7 |
| Data items | 10a | List and define all outcomes for which data were sought. Specify whether all results that were compatible with each outcome domain in each study were sought (e.g. for all measures, time points, analyses), and if not, the methods used to decide which results to collect. | 5-7 |
|  | 10b | List and define all other variables for which data were sought (e.g. participant and intervention characteristics, funding sources). Describe any assumptions made about any missing or unclear information. | 6-7 |
| Study risk of bias assessment | 11 | Specify the methods used to assess risk of bias in the included studies, including details of the tool(s) used, how many reviewers assessed each study and whether they worked independently, and if applicable, details of automation tools used in the process. | 6-7 |
| Effect measures | 12 | Specify for each outcome the effect measure(s) (e.g. risk ratio, mean difference) used in the synthesis or presentation of results. | 7 |
| Synthesis methods | 13a | Describe the processes used to decide which studies were eligible for each synthesis (e.g. tabulating the study intervention characteristics and comparing against the planned groups for each synthesis (item #5)). | 5-7 |
|  | 13b | Describe any methods required to prepare the data for presentation or synthesis, such as handling of missing summary statistics, or data conversions. | 7 |
|  | 13c | Describe any methods used to tabulate or visually display results of individual studies and syntheses. | 7 |
|  | 13d | Describe any methods used to synthesize results and provide a rationale for the choice(s). If meta-analysis was performed, describe the model(s), method(s) to identify the presence and extent of statistical heterogeneity, and software package(s) used. | 7 |
|  | 13e | Describe any methods used to explore possible causes of heterogeneity among study results (e.g. subgroup analysis, meta-regression). | 7 |
|  | 13f | Describe any sensitivity analyses conducted to assess robustness of the synthesized results. | 7 |
| Reporting bias assessment | 14 | Describe any methods used to assess risk of bias due to missing results in a synthesis (arising from reporting biases). | 7 |
| Certainty assessment | 15 | Describe any methods used to assess certainty (or confidence) in the body of evidence for an outcome. | 7 |
| **RESULTS** | | |  |
| Study selection | 16a | Describe the results of the search and selection process, from the number of records identified in the search to the number of studies included in the review, ideally using a flow diagram. | 8-10 |
|  | 16b | Cite studies that might appear to meet the inclusion criteria, but which were excluded, and explain why they were excluded. | 8 |
| Study characteristics | 17 | Cite each included study and present its characteristics. | 8-9 |
| Risk of bias in studies | 18 | Present assessments of risk of bias for each included study. | 10 |
| Results of individual studies | 19 | For all outcomes, present, for each study: (a) summary statistics for each group (where appropriate) and (b) an effect estimate and its precision (e.g. confidence/credible interval), ideally using structured tables or plots. | 10-13 |
| Results of syntheses | 20a | For each synthesis, briefly summarise the characteristics and risk of bias among contributing studies. | 10-13 |
|  | 20b | Present results of all statistical syntheses conducted. If meta-analysis was done, present for each the summary estimate and its precision (e.g. confidence/credible interval) and measures of statistical heterogeneity. If comparing groups, describe the direction of the effect. | 10-13 |
|  | 20c | Present results of all investigations of possible causes of heterogeneity among study results. | 10-13 |
|  | 20d | Present results of all sensitivity analyses conducted to assess the robustness of the synthesized results. | 10-13 |
| Reporting biases | 21 | Present assessments of risk of bias due to missing results (arising from reporting biases) for each synthesis assessed. | 10-13 |
| Certainty of evidence | 22 | Present assessments of certainty (or confidence) in the body of evidence for each outcome assessed. | 10-13 |
| **DISCUSSION** | | |  |
| Discussion | 23a | Provide a general interpretation of the results in the context of other evidence. | 13-17 |
|  | 23b | Discuss any limitations of the evidence included in the review. | 13-17 |
|  | 23c | Discuss any limitations of the review processes used. | 13-17 |
|  | 23d | Discuss implications of the results for practice, policy, and future research. | 13-17 |
| **OTHER INFORMATION** | | |  |
| Registration and protocol | 24a | Provide registration information for the review, including register name and registration number, or state that the review was not registered. | 5 |
|  | 24b | Indicate where the review protocol can be accessed, or state that a protocol was not prepared. | 5 |
|  | 24c | Describe and explain any amendments to information provided at registration or in the protocol. | 5 |
| Support | 25 | Describe sources of financial or non-financial support for the review, and the role of the funders or sponsors in the review. | 1 |
| Competing interests | 26 | Declare any competing interests of review authors. | 1 |
| Availability of data, code and other materials | 27 | Report which of the following are publicly available and where they can be found: template data collection forms; data extracted from included studies; data used for all analyses; analytic code; any other materials used in the review. | 1 |

*From:*  Page MJ, McKenzie JE, Bossuyt PM, Boutron I, Hoffmann TC, Mulrow CD, et al. The PRISMA 2020 statement: an updated guideline for reporting systematic reviews. BMJ 2021;372:n71. doi: 10.1136/bmj.n71. This work is licensed under CC BY 4.0. To view a copy of this license, visit <https://creativecommons.org/licenses/by/4.0/>
